# The FDA-approved anti-cancer drugs, streptozotocin and floxuridine, reduce the virulence of *Staphylococcus aureus*

**DOI:** 10.1038/s41598-018-20617-5

**Published:** 2018-02-06

**Authors:** Won-Sik Yeo, Rekha Arya, Kyeong Kyu Kim, Hyunyoung Jeong, Kyu Hong Cho, Taeok Bae

**Affiliations:** 1 Department of Microbiology and Immunology, Indiana University School of Medicine-Northwest, Gary, Indiana 46408 USA; 2 Department of Molecular Cell Biology, Sungkyunkwan University School of Medicine, Samsung Medical Center, Suwon, 16419 Korea; 3 Departments of Pharmacy Practice and Biopharmaceutical Sciences, University of Illinois at Chicago, Chicago, Illinois 60607 USA; 4 Department of Biology, Indiana State University, Terre Haute, IN 47809 USA

## Abstract

In *Staphylococcus aureus*, an important Gram-positive human pathogen, the SaeRS two-component system is essential for the virulence and a good target for the development of anti-virulence drugs. In this study, we screened 12,200 small molecules for Sae inhibitors and identified two anti-cancer drugs, streptozotocin (STZ) and floxuridine (FU), as lead candidates for anti-virulence drug development against staphylococcal infections. As compared with STZ, FU was more efficient in repressing Sae-regulated promoters and protecting human neutrophils from *S. aureus*-mediated killing. FU inhibited *S. aureus* growth effectively whereas STZ did not. Intriguingly, RNA-seq analysis suggests that both compounds inhibit other virulence-regulatory systems such as Agr, ArlRS, and SarA more efficiently than they inhibit the Sae system. Both compounds induced prophages from *S. aureus*, indicating that they cause DNA damages. Surprisingly, a single administration of the drugs was sufficient to protect mice from staphylococcal intraperitoneal infection. Both compounds showed *in vivo* efficacy in a murine model of blood infection too. Finally, at the experimental dosage, neither compound showed any noticeable side effects on blood glucose level or blood cell counts. Based on these results, we concluded that STZ and FU are promising candidates for anti-virulence drug development against *S. aureus* infection.

## Introduction

The emergence of multi-drug resistant bacterial pathogens is a huge medical problem^1^. The problem is compounded by the fact that the number of new antibiotics entering markets is decreasing^2^. Also, the growth inhibition activity of antibiotics inevitably selects for resistant strains^3,4^. Therefore, to tackle the problem, along with the development of new class of antibiotics, we need to take a broad range of alternative approaches such as phage therapy and development of anti-quorum sensing drugs or anti-virulence drugs^5,6^.

*S. aureus* is a Gram-positive human pathogen colonizing skin, anterior nares and other mucosal surface and causes a variety of diseases ranging from skin and soft-tissue infections to life-threatening diseases such as endocarditis, toxic shock syndrome, and necrotizing pneumonia^7,8^. The success of *S. aureus* as a human pathogen is mainly due to the production of a large number of virulence factors. In *S. aureus*, the production of virulence factors is coordinately controlled by multiple regulators such as SarA, Agr, ArlRS, and the SaeRS two-component system (TCS)^9–12^. Of those, the SaeRS TCS is an excellent target for anti-virulence drug development^13–15^. Conserved in all clinical *S. aureus* strains, the SaeRS TCS controls the production of more than 20 important virulence factors including toxins (e.g., alpha-hemolysin, gamma hemolysin, and leukocidins), coagulases, adhesins, and enzymes (e.g., nucleases and proteases)^12^. More importantly, the SaeS’s kinase activity correlates with the bacterial virulence in mice^16^, suggesting that the SaeRS system is a viable target for the development of anti-virulence drugs against staphylococcal infections. Since no structural information is available for SaeS, however, a rational design of Sae inhibitors is not feasible yet.

In this study, by taking a high-throughput approach with a GFP-reporter system for the SaeRS TCS, we screened small molecule libraries for Sae-inhibitors and found that two anti-cancer drugs have excellent *in vivo* efficacy in a murine model of staphylococcal infection. To understand their *in vivo* efficacy, we further studied the effect of the compounds on *S. aureus*.

## Results

### Screening small compound libraries for Sae inhibitors

**Figure 1:**
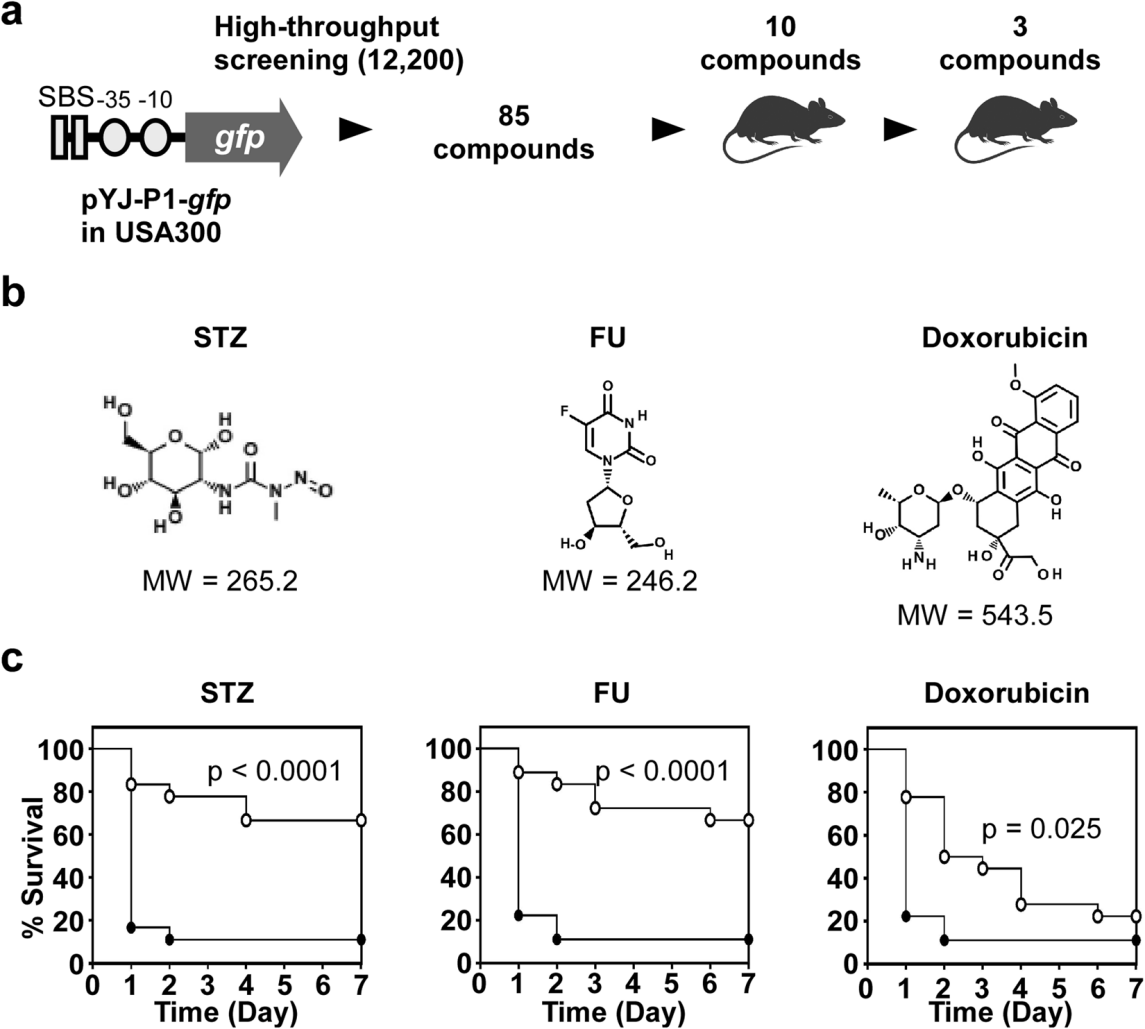
Identification of three anti-cancer agents with *in vivo* efficacy. (**a**) Overall procedure of the screening process. The number in parenthesis is the total number of compounds screened. The promoter sequences, −35 and −10, in the P1 promoter are indicated. SBS, the SaeR binding site. Parts of images were adapted from Motifolio Drawing Toolkits (www.motifolio.com). (**b**) Chemical structures and molecular weight of the identified compounds. (**c**) *In vivo* efficacy of the identified compounds. *S. aureus* (2 × 10^8^ CFU) was i.p. injected into 18 mice. At 1 h post-infection, the corresponding compounds (100 μg, 5 mg/kg body weight) were i.p. injected once every day for 7 days. Statistical significance was assessed by Log-rank test. STZ, streptozotocin; FU, floxuridine.

With two SaeR binding sites, the P1 promoter of the *sae* operon is an excellent reporter for the Sae activity^17^ (Fig. 1a). The P1-*gfp* fusion was cloned in the multi-copy plasmid pYJ335, and the resulting plasmid pYJ-P1-*gfp* was inserted into *S. aureus* strain USA300, the predominant CA-MRSA (community associated-methicillin resistant *S. aureus*) in the USA^18^. Using the reporter strain, we screened 12,200 small compounds for Sae-inhibition activity and identified 85 compounds that suppress the GFP expression by more than 50% at 10 μM. Subsequently, all 85 compounds were studied in a murine model of intraperitoneal infection, and, out of the initial tests, 10 compounds showed *in vivo* efficacy to some extent. When the *in vivo* experiment was repeated for these 10 compounds, the following three FDA-approved anti-cancer drugs consistently showed statistically significant *in vivo* efficacy: streptozotocin (STZ), floxuridine (FU) and doxorubicin (Fig. 1b and c). The structures of the compounds are different from other reported TCS-inhibitors^19–22^. Due to their excellent *in vivo* efficacy, STZ and FU were further studied.

### Repression of the SaeRS system by STZ and FU

**Figure 2:**
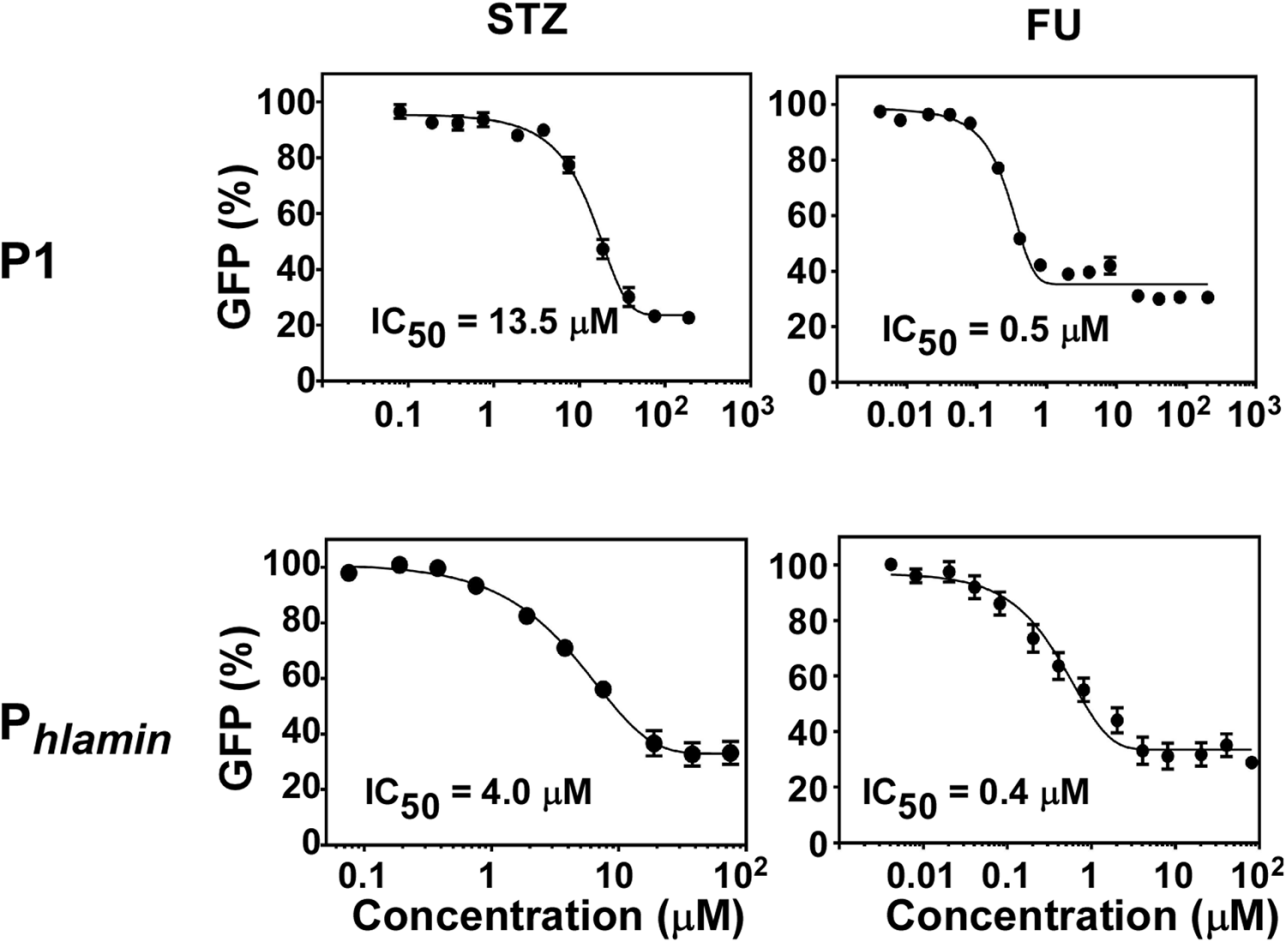
Repression of the SaeRS system by the anti-cancer agents. *S. aureus* USA300 carrying either pYJ-P1-*gfp* or pCL-P_hlamin_-*gfp* was grown to exponential growth phase in TSB; then a varying concentration of the anti-cancer agents was added. At 3 h post-incubation, GFP expression was measured and normalized by OD_600_.

Along with the P1 promoter of the *sae* operon, the alpha-hemolysin promoter (P_*hla*_) is another well-characterized target of the SaeRS TCS^17,23^. In particular, the minimal P_*hla*_ (P_*hlamin*_) has only the SaeR binding sites and the −35 and −10 promoter sequence of P_*hla*_. To compare their Sae-inhibitory activity, we measured IC_50_ (50% inhibitory concentration) of STZ and FU for P1 and P_*hlamin*_. As shown in Fig. 2, FU repressed the Sae-target promoters more efficiently than STZ did (0.5 μM vs. 13.5 μM for P1; 0.4 μM vs. 4.0 μM for P_*hlamin*_). Neither compound repressed the promoters of non-Sae-targets such as *mecA* and *hrtB* (Supplementary Fig. 1), indicating that the repression is target-specific.

### Protection of neutrophils from *S. aureus*-mediated killing

**Figure 3:**
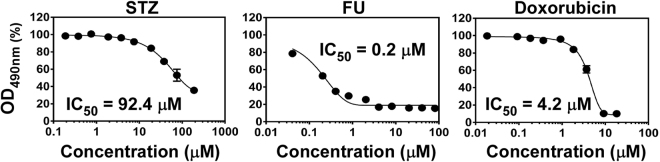
Protection of human neutrophils by the anti-cancer drugs. *S. aureus* USA300 (10^6^ CFU) and human neutrophils (10^5^ cells) were mixed, and the test compounds were added to the concentration indicated for 4 h. The viability of human neutrophils was measured by CellTiter assay (Promega). In the graph, the OD_490_ in the absence of compound was set to 100%.

Neutrophils are the most abundant white blood cells in human blood and critical for the defense against staphylococcal infection^24^. However, it is also well established that *S. aureus* can kill human neutrophils^25^. To understand the protective effect of STZ and FU on the host, we assessed whether the compounds could protect human neutrophils from killing by *S. aureus*. As with the Sae inhibition assay, FU protected human neutrophils from *S. aureus*-mediated killing much more efficiently than STZ did (IC_50_, 0.2 μM vs. 92.4 μM) (Fig. 3). Since STZ and FU showed similar *in vivo* efficacy (Fig. 1), these results might indicate that the neutrophil protection activity of a compound is not a good indicator for its *in vivo* efficacy. To examine this notion further, we measured IC_50_ for doxorubicin, which showed the least *in vivo* efficacy among them (Fig. 1). Again, doxorubicin protected human neutrophil more efficiently than STZ did (IC_50_, 4.2 μM vs. 92.4 μM) (Fig. 3), showing that the neutrophil-protection activity of a compound does not correlate well with its *in vivo* efficacy in a murine model of intraperitoneal infection.

### Bacterial growth inhibition by STZ and FU

STZ and FU are known to have not only anti-cancer activity but also antibacterial activity^26,27^. Therefore, it is possible that the excellent *in vivo* efficacy of the compounds is due to their antibacterial activity. To examine this possibility, we determined the MIC (minimum inhibitory concentration) of the compounds using *S. aureus* USA300 in two different growth media: Mueller Hinton broth and tryptic soy broth. In the condition employed, STZ showed almost negligible antibacterial activity whereas FU inhibited staphylococcal growth efficiently (Table 1). As compared with STZ, even doxorubicin showed more than 10 times higher activity of growth inhibition. Therefore, it is possible that the *in vivo* efficacy of FU might be, in part, due to its growth inhibitory activity, whereas the *in vivo* efficacy of STZ cannot be explained by its antibacterial activity.

**Table 1:** MIC of the anti-cancer agents against *S. aureus*.

Compounds	Mueller Hinton Broth	Tryptic Soy Broth
Streptozotocin	>256 μg/mL (965 μM)	>256 μg/mL (965 μM)
Floxuridine	0.0625 μg/mL (0.25 μM)	0.2 μg/mL (0.81 μM)
Doxorubicin	32 μg/mL (58.9 μM)	10 μg/mL (18.4 μM)

### Transcriptional alteration in *S. aureus* by STZ and FU

**Figure 4:**
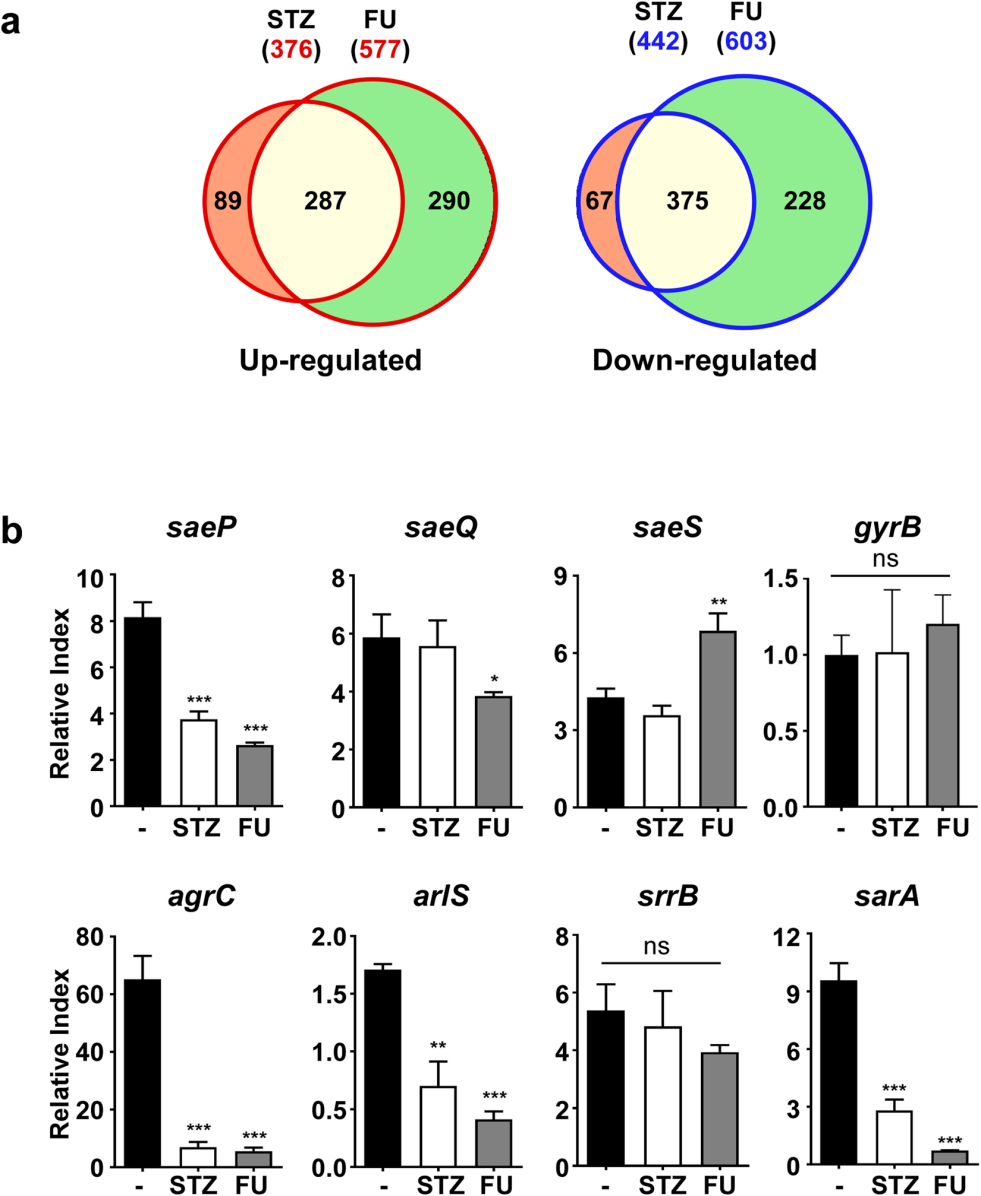
The effect of streptozotocin (STZ) and floxuridine (FU) on the transcription in *S. aureus* USA300. (**a**) Summary of RNA-seq analysis. (**b**) Confirmation of the repression of other virulence regulatory systems by qRT-PCR. Y-axis indicates the mRNA level relative to that of *gyrB*, whose transcription was not significantly affected by the compounds. -, no treatment. Statistical significance was measured by unpaired, two-tailed Student’s t-test. *p < 0.05; **p < 0.01; ***p < 0.001; ns, not significant.

To understand the molecular basis of the *in vivo* efficacy of STZ and FU, we analyzed genome-wide transcriptional changes caused by the compounds. *S. aureus* USA300 at exponential growth phase was treated with 1 μg/mL (~4 μM) of either compound at 37 °C for 3 h (Supplementary Fig. 2); then total RNA was purified and subjected to RNA-seq. Both STZ and FU caused significant transcriptional changes in *S. aureus* (STZ, 818 genes; FU 1180 genes, Fig. 4a, and Supplementary Table 1–4). The majority of STZ-affected genes (76% up-regulated and 85% of down-regulated) were also similarly affected by FU, indicating that most anti-virulence mechanisms of STZ are shared by FU. At 4 μM, STZ is not expected to inhibit the SaeRS TCS (Fig. 2). Indeed, in the qRT-PCR analysis, STZ did not reduce the transcription of *saeQ* and *saeS* (Fig. 4b), although it did reduce the transcription of *saeP*. On the other hand, FU reduced the transcription of *saeP* and *saeQ*, but it increased the transcription of *saeS* (Fig. 4b). The molecular mechanism of the differential *sae-*repression is not clear. Intriguingly, both compounds appeared to inhibit the transcription of other virulence-regulatory systems such as Agr, ArlRS, and SarA more effectively than they inhibit the Sae system (Fig. 4b). The repression also appears to be specific because the transcription of the control gene *gyrB* and most other staphylococcal two-component systems including the SrrAB TCS was not significantly affected (*gyrB* and *srrB* in Fig. 4b and Supplementary Tables 1–4). Taken altogether, these data suggest that the highly effective *in vivo* efficacy of STZ and FU could be due to their repression of multiple regulatory systems.

### Prophage induction by STZ and FU

**Figure 5:**
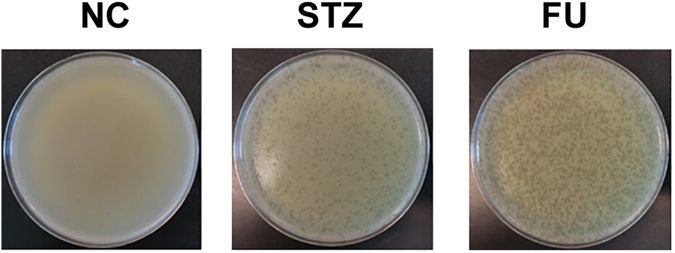
Prophage induction by streptozotocin (STZ) and floxuridine (FU). NC, No compound.

Most clinical isolates of *S. aureus* carry prophages. Since induction of prophages kills the *S. aureus* host, it can be an effective antibacterial mechanism. The genome of the strain USA300 contains two prophages (ΦSa2usa and ΦSa3usa), of which ΦSa2usa is replication-defective^28^. The RNA-seq analysis showed that the treatment with the anti-cancer drugs increased the transcription of ΦSa3usa genes (Supplementary Tables S1 and S3), indicating induction of the prophage. To confirm the results, we grew *S. aureus* USA300 in TSB containing either STZ or FU (0.2 μg/mL) for 18 h and analyzed the culture supernatant by the soft-agar method. As shown, the culture supernatants produced a large number of plaques whereas the non-treated culture supernatant did not (Fig. 5), confirming the prophage induction by the compounds. These results also indicate that both compounds probably cause damages in the staphylococcal chromosome and that the prophage induction likely contributes to *in vivo* efficacy of the compounds.

### Dose-dependent *in vivo* efficacy of STZ and FU

**Figure 6:**
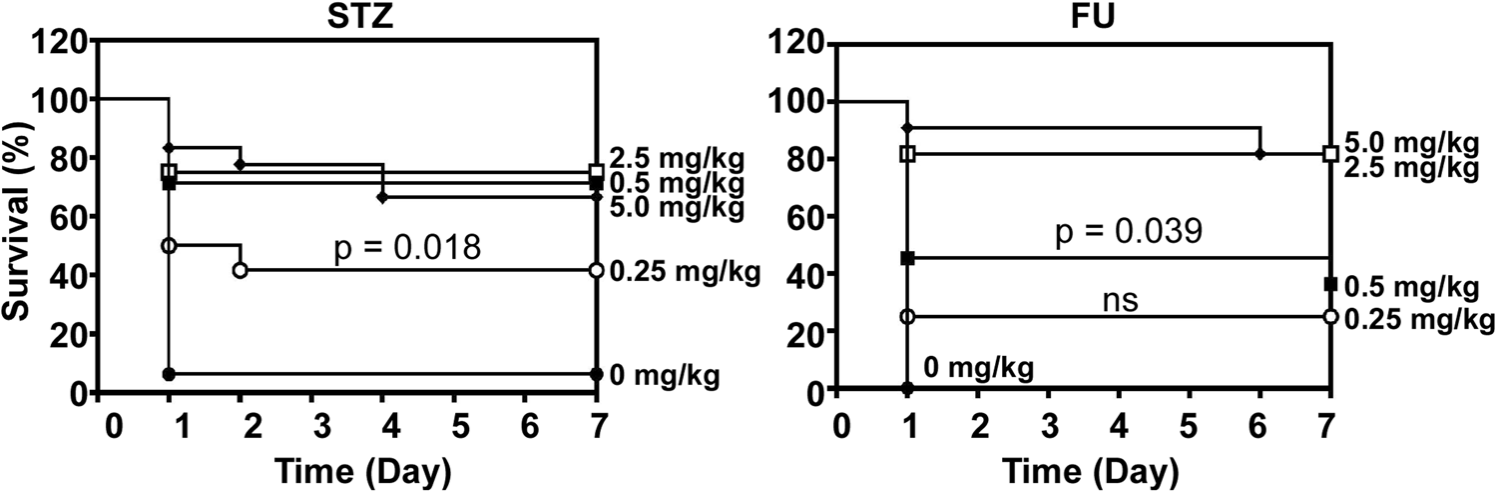
Dose-dependent efficacy of streptozotocin (STZ) and floxuridine (FU). Mice (11–17 e.a.) were i.p. injected with *S. aureus* USA300 (2 × 10^8^ CFU). At 1 h post-infection, a various amount of the compounds was i.p. injected. The compounds were administered once per day for 7 days. ns, not significant. The statistical significance was measured by Log-rank test.

Next, we tested the dose-dependency of STZ and FU in their protective effect on the mice. A daily dose of 0.25 mg/kg (STZ) or 0.5 mg/kg (FU) was sufficient to show statistically significant protection against *S. aureus* infection (Fig. 6).

### Effect of administration frequency on *in vivo* efficacy of STZ and FU

**Figure 7:**
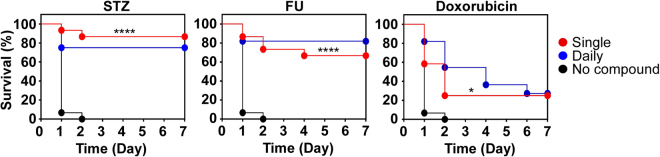
Effect of single administration of the compounds on murine survival. Fifteen mice were used for streptozotocin (STZ, 50 μg) and floxuridine (FU, 50 μg) while ten mice were used for doxorubicin (50 μg). For a comparison purpose, the daily administration results were superimposed. Statistical significance was measured against the no compound control (black circle) by Log-rank test. ****p < 0.0001; *p < 0.05.

The biological half-life of STZ is 35–40 min^29^. The half-life of FU is also rather short (t_1/2_β = 2 h in human)^30^. Although in the previous experiments, the compounds were administered every day, due to their short half-lives, it is likely that *S. aureus* cells were exposed to the compounds for a brief period. Therefore, we hypothesized that a brief exposure to the compounds is sufficient to give significant *in vivo* efficacy. To examine the hypothesis, at 1 h post-infection with *S. aureus*, we administered the compounds only once (1.25 mg/kg body weight for STZ and FU; 2.5 mg/kg body weight for doxorubicin) and watched the infected mice for 7 days. As shown in Fig. 7, the single administration of the compounds showed *in vivo* efficacy similar to that of daily administration.

### *In vivo* efficacy of STZ and FU in a murine model of blood infection

**Figure 8:**
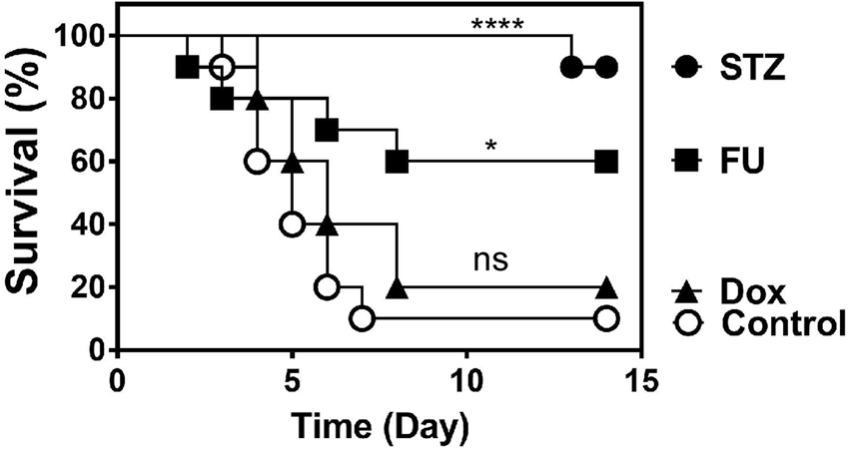
Efficacy of the anti-cancer drugs (50 μg) on staphylococcal blood infection. Ten mice were used for each condition. STZ, streptozotocin; FU, floxuridine; Dox, doxorubicin. Statistical significance was measured by Log-rank test against no drug control. ****p < 0.0001; *p < 0.05; ns, not significant. The survival difference between the STZ-treated mice and the FU-treated mice is not statistically significant (p = 0.105).

In our drug screening and subsequent animal studies, we used a murine model of intraperitoneal infection. However, the peritoneum is not a typical site of *S. aureus* infection. Therefore, STZ and FU were further tested in a murine model of blood infection, a common type of staphylococcal infections. *S. aureus* USA300 was administered into mice via retro-orbital injection; then, at 1 h post-infection, the compounds (2.5 mg/kg body weight) were injected into the mice via the intraperitoneal route. The drugs were administered once every day, and the mice were watched for 2 weeks. As shown in Fig. 8, both STZ and FU showed a statistically significant protective effect on murine survival whereas doxorubicin did not, confirming *in vivo* efficacy of STZ and FU in a clinically relevant animal model.

### Effects of STZ and FU on murine blood glucose and bone marrow

**Figure 9:**
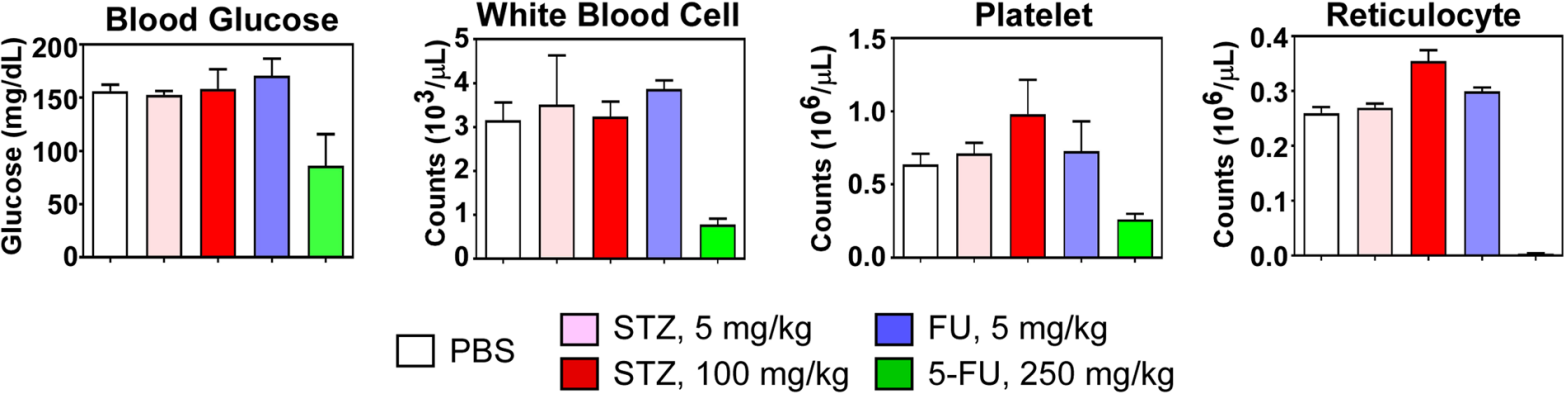
Effect of streptozotocin (STZ) and floxuridine (FU) on murine blood glucose and bone marrow depression. STZ and FU of the indicated amount were i.p. injected into three female mice. Seven days later, mice were sacrificed, and blood glucose concentration and blood cell counts were measured. PBS, phosphate buffered saline; 5-FU, 5′-fluorouracil. PBS and 5-FU were used as a negative and positive control, respectively. The normal blood glucose level was reported to be 153–171 mg/dL^49^. The normal values of cell counts are as follows: WBC, 3.2–12.7 (10^3^/μL); Platelet, 0.766–1.657 (10^6^/μL); Reticulocyte, 0.1258–0.469 (10^6^/μL).

At high doses, STZ is known to induce diabetes in rodents by killing β cells in the pancreas^31,32^. Besides, a high dose of 5-fluorouracil (5-FU), a metabolite of FU, is known to cause bone-marrow depression^33^. Therefore, we examined whether the dosage used in our animal experiment can cause those side effects. As expected, the 5-FU-treated mice showed a lower level of blood cell counts along with a sign of hypoglycemia, whereas the mice treated with either STZ or FU showed no such changes (Fig. 9). Even at 100 mg/kg, 20 times higher dose than the highest dose we used, STZ did not cause any significant changes in either blood glucose level or blood cell counts. These results indicate that both STZ and FU can reduce staphylococcal virulence at a safe dosage.

## Discussion

In this study, through a small molecule screening with a reporter for the SaeRS TCS, we identified two anti-cancer drugs, STZ and FU, as attractive lead candidates for novel anti-virulence drugs against staphylococcal infections. For lead candidates, these two compounds have the following promising properties: (1) simple molecular structure with low molecular weight (Fig. 1); (2) excellent *in vivo* efficacy even at low dosage and with single administration (Figs 1 and 6); (3) repression of multiple virulence regulators (Fig. 4); and (4) no overt adverse effect at the administration dosage (Fig. 9). In particular, with its excellent anti-growth and anti-virulence activities against *S. aureus*, FU has a potential to be developed into a dual agent, which inhibits not only the growth but also the virulence of *S. aureus*.

Although three compounds identified in the screening are all anti-cancer drugs, not all anti-cancer drugs have anti-staphylococcal activity. In the 12,200 small compounds screened in this study, 89 anti-cancer drugs were included. However, only three drugs, STZ, FU, and doxorubicin, repressed the Sae system and showed *in vivo* efficacy against *S. aureus* infection (Fig. 1c), suggesting that the anti-cancer activity itself is not sufficient to inhibit the SaeRS system and to protect the host from *S. aureus*-mediated killing. Intriguingly, of the 85 compounds showing anti-Sae activity in the initial screening (Fig. 1a), only three compounds showed significant *in vivo* activity, demonstrating that the *in vitro* Sae-inhibition activity does not guarantee *in vivo* efficacy of the compound. Since the *sae*-deletion mutant completely lost its virulence at the bacterial dosage used in the experiment^16^, these results imply that a partial repression of the Sae system is not sufficient to protect the host from staphylococcal infections. The repression of multiple regulatory systems (Fig. 4) and phage induction (Fig. 5) likely contribute to the *in vivo* efficacy of STZ and FU. Also, other pharmacological characteristics including *in vivo* half-life, plasma protein binding, and toxicity will also affect the overall *in vivo* efficacy of a compound. Therefore, in a drug development process, it might be prudent to employ an *in vivo* efficacy assay at an early stage as possible.

Both STZ and FU are known to have antibacterial activity. In fact, STZ was initially identified as an antibiotic that inhibits the growth of both Gram-negative and Gram-positive bacteria including *S. aureus*^26^. In the original study, STZ was reported to inhibit *S. aureus* growth by 50% at 0.75 μg/mL^26^. FU was also reported to be a very potent inhibitor of staphylococcal growth (MIC, 0.025–0.00313 μM)^34^. However, in our study, STZ showed a negligible anti-growth effect on *S. aureus* USA300 (Table 1). Although much more potent than STZ, the anti-growth effect of FU was about 10 times lower than that reported previously (Table 1). These discrepancies might be due to the differences in the test strain and the assay conditions employed. Interestingly, despite its low growth-inhibition activity, STZ showed a robust efficacy *in vivo* similar to that of FU (Figs 1, 6–8), signifying the importance of the anti-virulence activity of the compounds. Since 76–85% of the transcriptional effect of STZ are also observed in *S. aureus* treated with FU (Fig. 4), it is likely that most of the anti-virulence effects of STZ are also shared by FU.

Quinolone antibiotics such as ciprofloxacin are known to induce prophages via SOS-response^35–38^. Indeed, the 2 h-treatment of *S. aureus* 8325 with ciprofloxacin induced 16 prophage genes^39^. Analysis of non-prophage genes affected by STZ, FU, or ciprofloxacin showed that only a total of 22 genes (11 up-regulated, and 11 down-regulated) were commonly regulated by the three compounds (Supplementary Fig. 3 and Supplementary Table 5), suggesting that the effect of ciprofloxacin on *S. aureus* is largely different from that of STZ or FU. Of the 11 up-regulated genes, eight are involved in DNA repair, showing the induction of SOS-response is the primary effect shared by the three compounds. Intriguingly, along with alpha-hemolysin (*hla*), the virulence regulatory systems, Agr and SarA, were also commonly repressed by all three compounds. Although the molecular mechanism of the repression is not known, it is tempting to hypothesize that the induction of SOS-response gives negative impact on the virulence gene expression. Also, since STZ and FU appear to inhibit Agr more effectively than they inhibit the Sae system (Fig. 4b), it is possible that the repression of *hla* is through the repression of Agr, not the Sae system^40^.

As with cancer cells, bacteria can replicate indefinitely as long as a permissible growth condition is provided. Indeed, similarities between cancer cells and bacterial pathogens have been noted: high replication rates, damages to the host, spreading and dissemination within the host, and rapid development of resistance against therapeutic agents^41^. Even the immune response against bacterial pathogens has been used to treat certain types of tumor^42–44^. Although those similarities might be purely superficial without any biological connections, it is surprising to see that, of the 12,200 compounds screened, three compounds with *in vivo* efficacy are all anti-cancer agents. It is likely that the rapidly replicating nature of both types of cells makes them vulnerable to the anti-replication activity of the anti-cancer agents. Nonetheless, it remains to be seen whether there are other similarities between the pathogenic bacteria and cancer cells that can be targeted by the same therapeutic agent.

## Methods

### Ethics Statement

The human subject experiment (i.e., purification of human neutrophils) was approved by the Indiana University Institutional Review Board (Study number: 1010002390). Before taking blood, informed written consent was obtained from each human subject. All experiments were performed in accordance with relevant guidelines and regulations. The animal protocol was approved by the Committee on the Ethics of Animal Experiments of the Indiana University School of Medicine-Northwest (Protocol Number: NW-43). The animal experiment was performed by following the Guide for the Care and Use of Laboratory Animals of the National Institutes of Health. Every effort was made to minimize the suffering of the animals.

### Bacterial strains and growth conditions

The *S. aureus* strains and plasmids used in this study are described in Supplementary Tables 6. *S. aureus* was grown in tryptic soy broth (TSB). When necessary, antibiotics were added to the growth media at the following concentrations: erythromycin, 10 μg/mL; and chloramphenicol, 5 μg/mL.

### Construction of a GFP-promoter fusion

The reporter plasmids, pYJ-P1-*gfp*, pCL-P_hlamin_-*gfp*, pCL-P_*mecA*_-*gfp*, and pCL-P_*hrtB*_-*gfp* were generated by a ligation independent cloning method^45^. First, vectors were PCR-amplified from pYJ-*gfp*^16^ with the primers P1969/P1747 or from pCL55-*gfp*^14^ with the primers P1990/P1991 (for pCL-P_*hlamin*_-*gfp*), P3016/P3017 (for pCL-P_*mecA*_-*gfp*), or P3286/P3287 (for pCL-P_*hrtB*_-*gfp*) (Supplementary Table 7). The insert DNA fragment containing the promoter sequence was amplified with primer pairs P1971/P1972 for P1, P1992/P1993 (for P_hlamin_), P3014/P3015 (for P_*mecA*_), or P3284/P3285 (for P_*hrtB*_) (Supplementary Table 7). The PCR products were treated with T4 DNA polymerase in the presence of dCTP (vector) or dGTP (insert DNA) and mixed. DNA mixture was used to transform *E. coli* DH5α. Once verified, all plasmids were electroporated into *S. aureus* strain RN4220 and subsequently transduced into *S. aureus* strain USA300 with ϕ85.

### High-throughput screening of small molecule libraries

*S. aureus* USA300 harboring pYJ-P1-*gfp* was used to screen 12,200 compounds (3,000 compounds from Spectrum Library [MicroSource Discovery Systems], 6,000 from the discovery collection library [DCL, MicroSource Discovery Systems], and 3,200 natural compounds [Indiana University]) in DMSO (stock concentration 10 mM). The compounds were dry transferred into 384 plates using an Echo 550 Acoustic Liquid Transfer System. *S. aureus* USA300 (pYJ-P1-*gfp*) was grown in TSB overnight. The overnight culture was diluted 1:100 into fresh TSB and grown until OD_600_ = 0.5. Then 30 μL of the culture was transferred into the plate, resulting in 10 μM of the compounds. A negative control (DMSO blank) was included in each plate. Initial cell density and GFP fluorescence were measured after filling the plates. Plates were then sealed and incubated on a plate shaker-incubator at 37 °C for 3 h. The GFP signal (excitation 485 nm, emission 515 nm) and OD_600_ were measured with a Tecan Infinite M1000 Microplate Reader. The GFP signal was normalized by OD_600_, and the normalized value was used to identify the Sae inhibitor candidates.

### Animal test

An overnight culture of *S. aureus* was 100-fold diluted into fresh TSB and further incubated at 37 °C for 4 h. Cells were collected by centrifugation, washed with sterile PBS, and suspended in sterile PBS to OD_600_ = 4 (i.e., 1 × 10^9^ CFU mL^−1^). Sex-matched 8-week-old C57BL/6 mice were i.p. injected with *S. aureus* (2 × 10^8^ CFU); then, at 1 h post-infection, a varying amount of compound was administered through i.p. injection. DMSO was used as a negative control. For daily dose experiment, the compound was i.p. injected every 24 h for seven days.

For retro-orbital studies for the compounds, the bacterial suspension (10^7^ CFU in 100 μL) was administered into 10 sex-matched 8-week-old C57BL/6 mice via retro-orbital injection. At 1 h post-infection, test compounds (50 μg, 2.5 mg/kg) was administered by i.p. injection. The compounds were i.p. injected every 24 h for 14 days.

Statistical significance in murine survival was measured by Log-rank (Mantel-Cox) test with Prism 6 (GraphPad).

### Determination of MIC

Minimum inhibitory concentration (MIC) for streptozotocin, floxuridine, and doxorubicin was determined according to the recommendations proposed by the Clinical and Laboratory Standards Institute (CLSI) using microdilution method^46^.

### RNA-seq analysis

An overnight culture of *S. aureus* in TSB was diluted 100 times in a fresh TSB (2 mL in a 15 mL test tube) and incubated in a shaking incubator at 37 °C for 2 h. Then 1 μg/mL of streptozotocin or floxuridine were added to the culture and further incubated for 3 h. After immediate stabilization of RNA in all samples by RNAprotect Bacteria Reagent (Qiagen), total RNA was isolated with RNeasy Mini Kit (Qiagen) according to the manufacturer’s recommendations. For each condition, three RNA samples were isolated from three independent bacterial cultures. The isolated RNAs were sent to the Center for Genomics and Bioinformatics at Indiana University. Sequencing libraries were constructed using the ScriptSeq Complete Kit for Bacteria (Epicentre). The statistical analysis of the RNA-seq results was done with DeSeq. 2 as described before^47^.

### qRT-PCR analysis

The total RNA used for RNA-seq was also used for qRT-PCR analysis. cDNA was synthesized using High Capacity cDNA Reverse Transcription Kit (Applied Biosystems). Quantification of transcripts was carried out by real-time PCR using SYBR Green PCR Master Mix (Applied Biosystems) in a QuantStudio 7 Flex Real-Time PCR System (Applied Biosystems). Primers used to detect specific transcripts are shown in Supplementary Table 6. The relative amount of cDNA was determined using a standard curve obtained from PCR with serially diluted genomic DNA, and results were normalized to the levels of *gyrB* RNA. Statistical significance was measured by unpaired, two-tailed Student’s t-test with Prism 6 (GraphPad).

### GFP assay

An overnight culture of *S. aureus* harboring pYJ-P1-*gfp*, pCL-_Phla min_-*gfp*, pCL-P_*mecA*_-*gfp*, or pCL-P_*hrtB*_-*gfp* was diluted 100 times in a fresh TSB and incubated in a shaking incubator at 37 °C for 2 h. Then various concentrations of streptozotocin and floxuridine were added to cells, and the cells were incubated for 3 h. The GFP signal (excitation 485 nm, emission 515 nm) and OD_600_ were measured with Enspire Plate Reader (Perkin Elmer). All GFP expressions were normalized by OD_600_. The half maximal inhibitory concentration (IC_50_) was calculated from at least three independent experiments via nonlinear regression analysis (sigmoidal dose-response with variable slope) using Prism 6 (GraphPad).

### Neutrophil killing assay

Human neutrophils were purified from healthy adult blood donors by Ficoll-Paque gradient method^48^. The purified neutrophils were added to a 96-well tissue culture plate (2 × 10^5^/well) in 100 μL RPMI (Gibco) supplemented with 10 mM HEPES and 10% human serum. *S. aureus* cells (1 × 10^6^ CFU) and various concentrations of streptozotocin, floxuridine, and doxorubicin were added to each well. Then, 10 μL of CellTiter 96® AQ_ueous_ One Solution (Promega) was added to each well and incubated at 37 °C in 5% CO_2_ for 3–5 h. Neutrophil viability was assessed with the Enspire plate reader (Perkin Elmer).

### Prophage induction by streptozotocin and floxuridine

*S. aureus* USA300 was grown in TSB containing 0.2 μg/mL of the compounds (STZ or FU) or no compound (a negative control) at 37 °C for 18 h; then the supernatant was collected by filtration (0.22 μm). The supernatant (100 μL) was mixed with the overnight culture (100 μL) of *S. aureus* RN4220 suspended in HIB (heart infusion broth) containing 5 mM of CaCl_2_. The mixture was incubated at 37 °C for 10 min, mixed with soft TSA (0.8%), spread on TSA, and incubated at 37 °C for 18 h.

### Blood glucose and bone marrow suppression tests in mice

Streptozotocin (0.1 mg [=5 mg/kg] and 2 mg [=100 mg/kg]) and floxuridine (0.1 mg, 5 mg/kg) were i.p. injected into 8-week-old C57BL/6 mice. PBS and 5-fluorouracil (5 mg, 250 mg/kg) were used a negative and positive control, respectively. Seven days later, mice were sacrificed, and blood was collected. The collected blood was sent to Biologic Resources Laboratory at the University of Illinois at Chicago, where blood cells were counted. Blood glucose concentration was measured by Contour blood glucose monitoring system (Asensia).

### Accession number

The RNA-seq data were deposited in GEO (Gene Expression Omnibus) with the accession number GSE104069.

### Data Availability

All processed RNA-seq results were included in Supplementary Tables 1–4. The raw data deposited to GEO (the accession number GSE104069) will be released on September 19, 2019.

## Electronic supplementary material

Supplementary Information
