# Supplementary material for: The FDA-approved anti-cancer drugs, streptozotocin and floxuridine, reduce the virulence of Staphylococcus aureus
Source: Sci Rep. 2018 Feb 6;8:2521. doi: 10.1038/s41598-018-20617-5 (PMC5802796; doi:10.1038/s41598-018-20617-5)
Supplement: Supplementary file 1 — Supplementary Information [file 41598_2018_20617_MOESM1_ESM.doc]

**Supplementary Information**

**The FDA-approved anti-cancer drugs, streptozotocin and floxuridine, reduce the virulence of *Staphylococcus aureus***

Won-Sik Yeo^1^, Rekha Arya^2^, Kyeong Kyu Kim^2^, Hyunyoung Jeong^3^, Kyu Hong Cho^4^,and Taeok Bae ^1*^

1. Department of Microbiology and Immunology, Indiana University School of Medicine-Northwest, Gary, Indiana, 46408, USA
2. Department of Molecular Cell Biology, Sungkyunkwan University School of Medicine, SMC, Suwon, 16322, Korea
3. Department of Pharmacy Practice and Biopharmaceutical Sciences, the University of Illinois at Chicago, Chicago, Illinois, 60607, USA
4. Department of Biology, Indiana State University, Terre Haute, IN 47809, USA

^*^ Corresponding author

Email: [tbae@iun.edu](mailto:tbae@iun.edu)


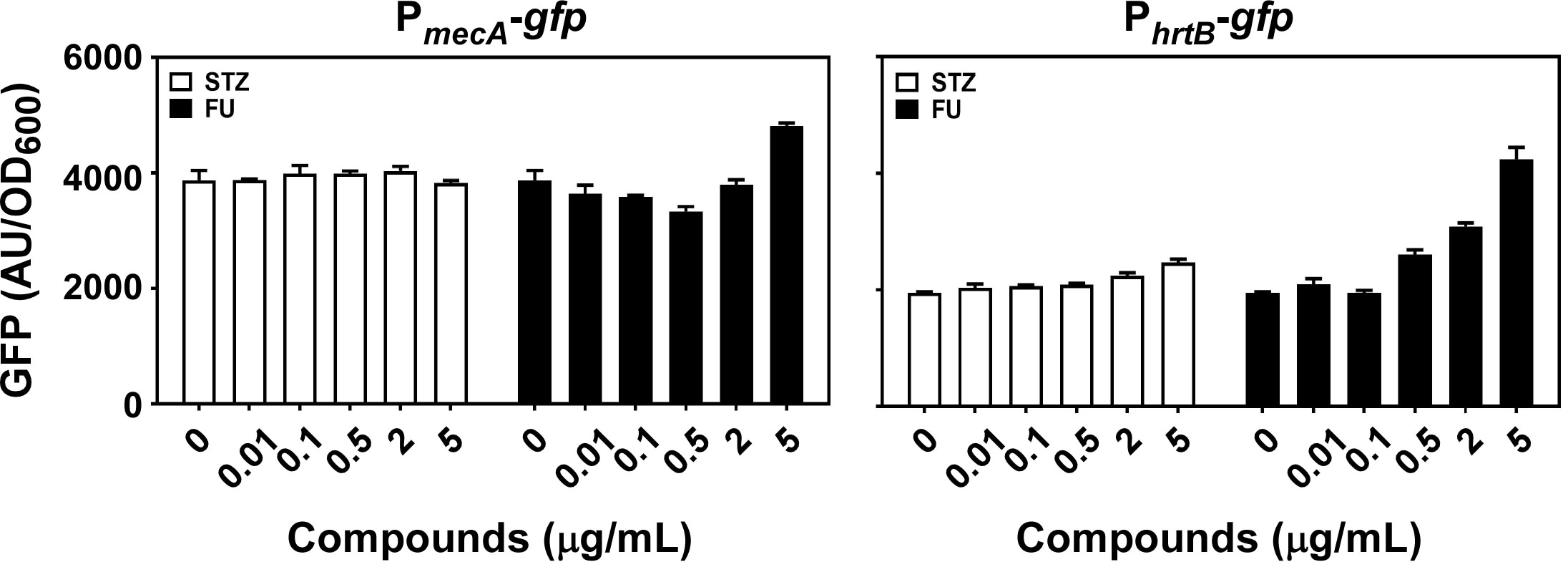


**Supplementary Fig. 1. The effect of streptozotocin (STZ) and floxuridine (FU) on the transcription from the promoters of *mecA* and *hrtB*.** AU, arbitrary unit.


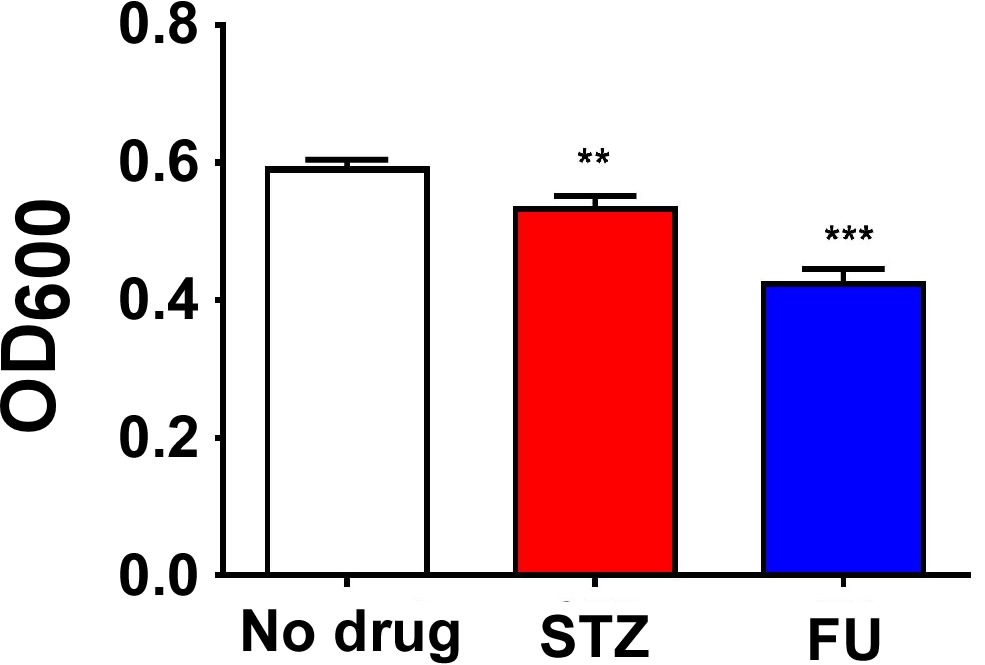


**Supplementary Fig. 2. The comparison of the bacterial growth in the condition for RNA-seq analysis.** Detail procedures are presented in Methods. From the final culture, 100 μL was used to measure OD600 with a multimode plate reader (EnSpire, PerkinElmer). The statistical analysis was carried out by two-tailed, unpaired t-test (Prism 7.03, GraphPad). **, p < 0.005; ***, p < 0.0001.

**
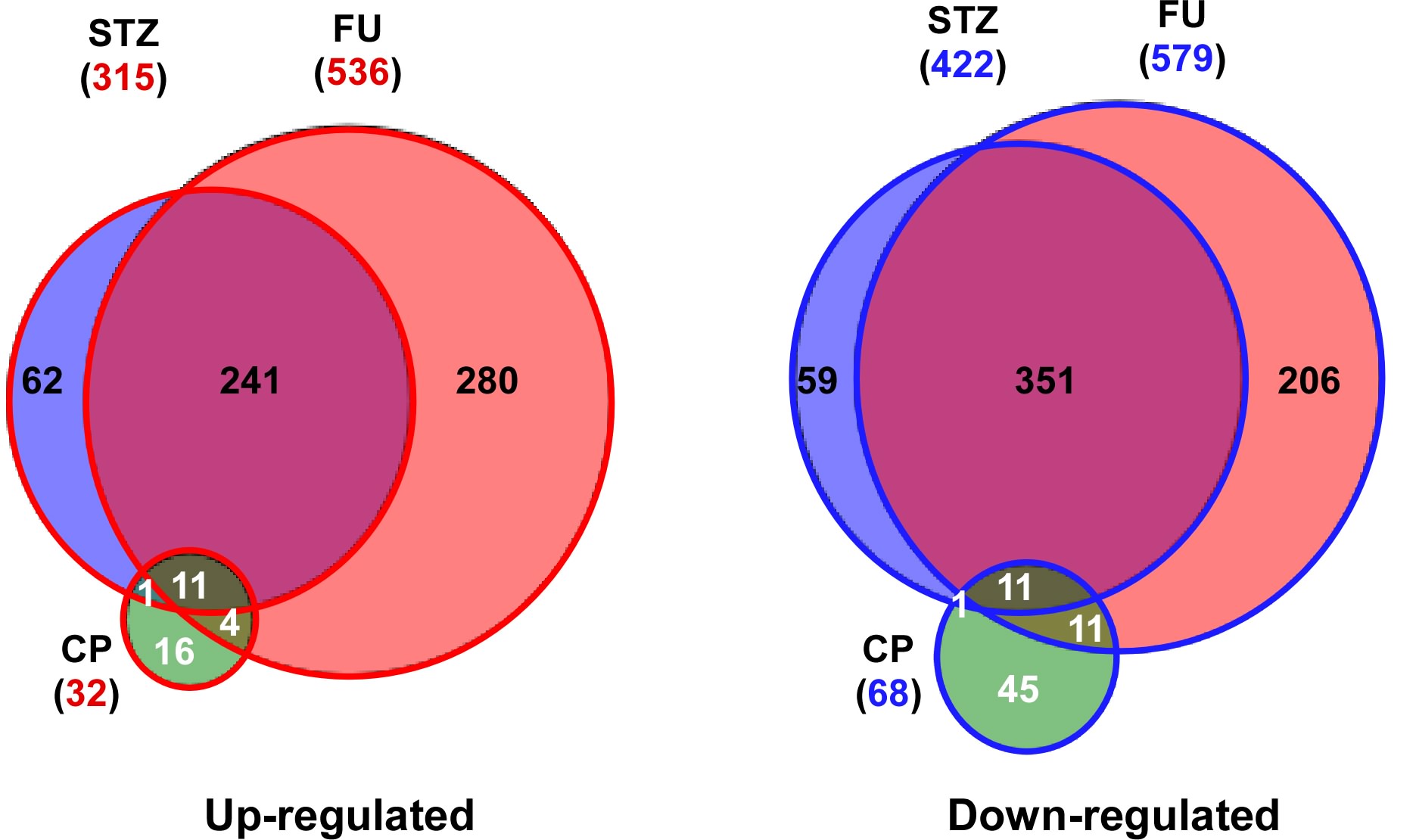
**

**Supplementary Fig. 3. Comparison of the genes affected by STZ, FU and ciprofloxacin (CP).** For STZ and FU, the USA300 strain was used whereas, for CP, the strain 8325 was used. Due to the strain differences, in this figure, all phages genes were excluded, which explains for the different gene numbers, as compared with those in Fig. 4a.

**Supplementary Table 1. Genes up-regulated by streptozotocin** (Pink color, the genes up-regulated by floxuridine)

| **Gene ID** | **Name** | **Product** | **p-value** | **Ratio** |
| --- | --- | --- | --- | --- |
| SAUSA300_0007 | . | conserved hypothetical protein | 0.000000 | 3.6 |
| SAUSA300_0011 | . | conserved hypothetical protein | 0.000000 | 2.7 |
| SAUSA300_0017 | purA | adenylosuccinate synthetase | 0.000042 | 3.3 |
| SAUSA300_0019 | . | tRNA-Asp | 0.000017 | 4.2 |
| SAUSA300_0024 | . | metallo-beta-lactamase family protein | 0.000000 | 2.2 |
| SAUSA300_0025 | . | 5-nucleotidase family protein | 0.000000 | 3.0 |
| SAUSA300_0036 | . | conserved hypothetical protein | 0.057796 | 6.3 |
| SAUSA300_0037 | ccrB | cassette chromosome recombinase B | 0.000000 | 3.7 |
| SAUSA300_0038 | ccrA | cassette chromosome recombinase A | 0.000045 | 2.1 |
| SAUSA300_0040 | . | conserved hypothetical protein | 0.078749 | 2.1 |
| SAUSA300_0047 | . | conserved hypothetical protein | 0.000006 | 2.2 |
| SAUSA300_0048 | . | hypothetical protein | 0.023644 | 2.0 |
| SAUSA300_0052 | . | hypothetical protein | 0.305522 | 2.1 |
| SAUSA300_0057 | . | conserved hypothetical protein | 0.017729 | 2.8 |
| SAUSA300_0059 | . | conserved hypothetical protein | 0.065864 | 3.6 |
| SAUSA300_0060 | . | putative transposase | 0.005073 | 2.0 |
| SAUSA300_0076 | . | ABC transporter, ATP-binding protein | 0.000015 | 2.2 |
| SAUSA300_0077 | . | ABC transporter, ATP-binding protein | 0.000221 | 2.4 |
| SAUSA300_0084 | . | conserved hypothetical protein | 0.003520 | 2.5 |
| SAUSA300_0092 | . | conserved hypothetical protein | 0.025184 | 2.2 |
| SAUSA300_0093 | . | transcriptional regulator, LysR family domain protein | 0.000000 | 2.7 |
| SAUSA300_0096 | . | conserved hypothetical protein | 0.000000 | 2.0 |
| SAUSA300_0113 | . | immunoglobulin G binding protein A precursor | 0.000000 | 7.3 |
| SAUSA300_0114 | . | staphylococcal accessory regulator | 0.000092 | 2.2 |
| SAUSA300_0116 | sirB | iron compound ABC transporter, permease protein SirB | 0.000592 | 2.2 |
| SAUSA300_0130 | . | NAD-dependent epimerase/dehydratase family protein | 0.007808 | 2.2 |
| SAUSA300_0132 | . | glycosyl transferase, group 1 family protein | 0.010294 | 2.4 |
| SAUSA300_0133 | . | putative membrane protein | 0.024676 | 2.2 |
| SAUSA300_0135 | . | Superoxide dismutase (Mn/Fe family) | 0.000000 | 2.0 |
| SAUSA300_0145 | . | phosphonate ABC transporter, phosphonate-binding protein | 0.000005 | 2.4 |
| SAUSA300_0148 | . | conserved hypothetical protein | 0.315646 | 2.8 |
| SAUSA300_0173 | . | conserved hypothetical protein | 0.000000 | 5.1 |
| SAUSA300_0174 | . | conserved hypothetical protein | 0.000000 | 3.9 |
| SAUSA300_0175 | . | putative lipoprotein | 0.000000 | 4.1 |
| SAUSA300_0176 | . | ABC transporter, permease protein | 0.000000 | 4.4 |
| SAUSA300_0177 | . | conserved hypothetical protein | 0.000000 | 2.6 |
| SAUSA300_0179 | . | putative D-isomer specific 2-hydroxyacid dehydrogenase | 0.000002 | 2.0 |
| SAUSA300_0187 | rocD | ornithine--oxo-acid transaminase | 0.000000 | 2.8 |
| SAUSA300_0191 | ptsG | PTS system, glucose-specific IIBC component domain protein | 0.000000 | 6.7 |
| SAUSA300_0201 | . | peptide ABC transporter, permease protein | 0.003213 | 2.1 |
| SAUSA300_0207 | . | conserved hypothetical protein | 0.000000 | 2.4 |
| SAUSA300_0208 | . | putative maltose ABC transporter, ATP-binding protein | 0.000000 | 3.0 |
| SAUSA300_0216 | uhpT | hexose phosphate transport protein | 0.001105 | 3.6 |
| SAUSA300_0230 | . | putative membrane protein | 0.000000 | 2.4 |
| SAUSA300_0236 | . | PTS system, IIBC components | 0.000000 | 2.8 |
| SAUSA300_0240 | . | PTS system, galactitol-specific enzyme II, B component | 0.277564 | 3.7 |
| SAUSA300_0254 | . | sensor histidine kinase | 0.000000 | 2.8 |
| SAUSA300_0255 | . | sensory transduction protein LytR | 0.000000 | 4.6 |
| SAUSA300_0261 | . | conserved hypothetical protein | 0.000000 | 3.4 |
| SAUSA300_0267 | . | transposase | 0.005001 | 3.3 |
| SAUSA300_0270 | lytM | peptidoglycan hydrolase | 0.000000 | 2.4 |
| SAUSA300_0302 | . | conserved hypothetical protein | 0.000013 | 2.1 |
| SAUSA300_0307 | . | 5-nucleotidase, lipoprotein e(P4) family | 0.000000 | 5.3 |
| SAUSA300_0360 | . | Cys/Met metabolism PLP-dependent enzyme | 0.001045 | 2.2 |
| SAUSA300_0365 | . | conserved hypothetical protein | 0.000000 | 71.4 |
| SAUSA300_0386 | xpt | xanthine phosphoribosyltransferase | 0.000000 | 3.1 |
| SAUSA300_0387 | pbuX | xanthine permease | 0.000000 | 2.3 |
| SAUSA300_0388 | guaB | inosine-5-monophosphate dehydrogenase | 0.000000 | 2.3 |
| SAUSA300_0389 | guaA | GMP synthase | 0.000000 | 2.2 |
| SAUSA300_0406 | . | putative restriction/modification system specificity protein | 0.000000 | 2.1 |
| SAUSA300_0428 | . | conserved hypothetical protein | 0.012413 | 2.6 |
| SAUSA300_0431 | . | conserved hypothetical protein | 0.250337 | 2.2 |
| SAUSA300_0432 | . | sodium dependent transporter | 0.000000 | 5.6 |
| SAUSA300_0433 | cysM | cysteine synthase/cystathionine beta-synthase | 0.000000 | 2.5 |
| SAUSA300_0434 | metB | cystathionine gamma-synthase | 0.000000 | 2.8 |
| SAUSA300_0438 | . | CHAP domain family | 0.000000 | 2.7 |
| SAUSA300_0443 | . | YibE/F-like protein | 0.000000 | 2.4 |
| SAUSA300_0445 | gltB | glutamate synthase, large subunit | 0.000000 | 2.1 |
| SAUSA300_0447 | . | tRNA-Ser | 0.000009 | 2.1 |
| SAUSA300_0448 | treP | PTS system, trehalose-specific IIBC component | 0.000000 | 4.8 |
| SAUSA300_0449 | treC | alpha,alpha-phosphotrehalase | 0.000000 | 3.2 |
| SAUSA300_0450 | treR | trehalose operon repressor | 0.000000 | 2.3 |
| SAUSA300_0457 | rrfA | 5S ribosomal RNA | 0.109910 | 2.9 |
| SAUSA300_0465 | . | conserved hypothetical protein | 0.001239 | 2.2 |
| SAUSA300_0466 | . | conserved hypothetical protein | 0.000000 | 2.1 |
| SAUSA300_0497 | rrfG | 5S ribosomal RNA | 0.000284 | 5.1 |
| SAUSA300_0502 | rrfB | 5S ribosomal RNA | 0.109910 | 2.9 |
| SAUSA300_0523 | rplA | ribosomal protein L1 | 0.000529 | 2.0 |
| SAUSA300_0542 | . | deoxynucleoside kinase family protein | 0.000001 | 2.5 |
| SAUSA300_0548 | sdrE | sdrE protein | 0.000000 | 2.1 |
| SAUSA300_0577 | . | putative transcriptional regulator | 0.000010 | 2.8 |
| SAUSA300_0578 | . | conserved hypothetical protein | 0.149223 | 3.2 |
| SAUSA300_0640 | . | putative membrane protein | 0.000000 | 2.1 |
| SAUSA300_0651 | . | CHAP domain family | 0.000000 | 3.9 |
| SAUSA300_0683 | . | transcriptional regulator, DeoR family | 0.000000 | 2.6 |
| SAUSA300_0697 | . | exsB protein | 0.000000 | 2.7 |
| SAUSA300_0713 | folE | GTP cyclohydrolase I | 0.000000 | 2.4 |
| SAUSA300_0714 | . | Integral membrane protein | 0.000000 | 2.1 |
| SAUSA300_0715 | nrdI | nrdI protein | 0.000000 | 2.7 |
| SAUSA300_0716 | . | ribonucleoside-diphosphate reductase, alpha subunit | 0.000000 | 3.2 |
| SAUSA300_0717 | . | ribonucleoside-diphosphate reductase, beta subunit | 0.000000 | 3.9 |
| SAUSA300_0718 | . | iron compound ABC transporter, permease | 0.000000 | 3.0 |
| SAUSA300_0719 | . | iron compound ABC transporter, permease protein | 0.000013 | 3.0 |
| SAUSA300_0720 | . | putative iron compound ABC transporter, ATP-binding protein | 0.000109 | 2.8 |
| SAUSA300_0721 | . | transferrin receptor | 0.000116 | 2.6 |
| SAUSA300_0734 | . | putative comf operon protein 1 | 0.001582 | 2.2 |
| SAUSA300_0739 | . | LysM domain protein | 0.000000 | 3.6 |
| SAUSA300_0741 | uvrB | excinuclease ABC, B subunit | 0.000000 | 5.9 |
| SAUSA300_0742 | uvrA | excinuclease ABC, A subunit | 0.000000 | 4.7 |
| SAUSA300_0752 | clpP | ATP-dependent Clp protease, proteolytic subunit ClpP | 0.000000 | 2.3 |
| SAUSA300_0756 | gap | glyceraldehyde-3-phosphate dehydrogenase, type I | 0.000000 | 2.1 |
| SAUSA300_0776 | nuc | thermonuclease precursor | 0.000000 | 9.9 |
| SAUSA300_0777 | . | cold shock protein | 0.000032 | 2.1 |
| SAUSA300_0782 | . | conserved hypothetical protein | 0.000183 | 2.6 |
| SAUSA300_0793 | . | conserved hypothetical protein | 0.000000 | 2.0 |
| SAUSA300_0796 | . | ABC transporter, ATP-binding protein | 0.000000 | 2.7 |
| SAUSA300_0797 | . | ABC transporter permease protein | 0.000000 | 3.1 |
| SAUSA300_0798 | . | ABC transporter, substrate-binding protein | 0.000000 | 4.0 |
| SAUSA300_0799 | int | integrase | 0.000000 | 13.0 |
| SAUSA300_0800 | sek | staphylococcal enterotoxin K | 0.000000 | 5.5 |
| SAUSA300_0801 | seq | staphylococcal enterotoxin Q | 0.000000 | 6.3 |
| SAUSA300_0802 | . | conserved hypothetical protein | 0.000000 | 6.6 |
| SAUSA300_0803 | . | transcriptional regulator, Cro/CI family | 0.000000 | 9.6 |
| SAUSA300_0804 | . | putative transcriptional regulator | 0.000000 | 186.3 |
| SAUSA300_0805 | . | pathogenicity island protein | 0.000000 | 174.5 |
| SAUSA300_0806 | . | conserved hypothetical protein | 0.000000 | 185.6 |
| SAUSA300_0807 | . | conserved hypothetical protein | 0.000000 | 167.6 |
| SAUSA300_0808 | . | conserved hypothetical protein | 0.000000 | 146.5 |
| SAUSA300_0809 | . | putative DNA primase | 0.000000 | 147.2 |
| SAUSA300_0810 | . | conserved hypothetical protein | 0.000000 | 98.0 |
| SAUSA300_0811 | . | conserved hypothetical protein | 0.000000 | 117.4 |
| SAUSA300_0812 | . | conserved hypothetical protein | 0.000000 | 108.9 |
| SAUSA300_0813 | . | conserved hypothetical protein | 0.000000 | 103.2 |
| SAUSA300_0814 | . | conserved hypothetical protein | 0.000000 | 7.8 |
| SAUSA300_0815 | ear | Ear protein | 0.000000 | 18.9 |
| SAUSA300_0830 | . | conserved hypothetical protein | 0.000000 | 2.3 |
| SAUSA300_0831 | . | conserved hypothetical protein | 0.000000 | 2.8 |
| SAUSA300_0836 | dltB | dltB protein | 0.000001 | 2.3 |
| SAUSA300_0837 | dltC | D-alanine-activating enzyme/D-alanine-D-alanyl, dltC protein | 0.000346 | 2.0 |
| SAUSA300_0838 | dltD | D-alanine-activating enzyme/D-alanine-D-alanyl, dltD protein | 0.000000 | 2.2 |
| SAUSA300_0843 | . | conserved hypothetical protein | 0.000000 | 2.3 |
| SAUSA300_0846 | . | Na+/H+ antiporter family protein | 0.000000 | 5.2 |
| SAUSA300_0848 | . | conserved hypothetical protein | 0.000000 | 4.0 |
| SAUSA300_0859 | . | NADH-dependent flavin oxidoreductase | 0.000000 | 2.6 |
| SAUSA300_0863 | argH | argininosuccinate lyase | 0.000000 | 7.1 |
| SAUSA300_0864 | argG | argininosuccinate synthase | 0.000000 | 5.6 |
| SAUSA300_0865 | pgi | glucose-6-phosphate isomerase | 0.000000 | 2.2 |
| SAUSA300_0867 | spsA | signal peptidase IA | 0.000000 | 2.2 |
| SAUSA300_0868 | spsB | signal peptidase IB | 0.000000 | 2.7 |
| SAUSA300_0875 | . | conserved hypothetical protein | 0.000000 | 2.1 |
| SAUSA300_0878 | . | transcriptional regulator, LysR family | 0.000014 | 2.9 |
| SAUSA300_0879 | . | isopropylmalate synthase-related protein | 0.057988 | 2.8 |
| SAUSA300_0899 | . | putative negative regulator of genetic competence | 0.000000 | 2.3 |
| SAUSA300_0900 | . | putative competence protein | 0.000000 | 33.7 |
| SAUSA300_0901 | . | putative competence protein | 0.000000 | 21.7 |
| SAUSA300_0902 | pepF | oligoendopeptidase F | 0.000000 | 2.2 |
| SAUSA300_0909 | . | pseudouridine synthases, RluA subfamily | 0.000000 | 2.0 |
| SAUSA300_0910 | mgtE | magnesium transporter | 0.000000 | 2.3 |
| SAUSA300_0914 | . | sodium:alanine symporter family protein | 0.000000 | 2.5 |
| SAUSA300_0917 | . | putative membrane protein | 0.000000 | 2.1 |
| SAUSA300_0922 | . | membrane protein, TerC family | 0.000000 | 2.6 |
| SAUSA300_0926 | . | tRNA-Ser | 0.000366 | 11.5 |
| SAUSA300_0932 | . | putative membrane protein | 0.000000 | 2.4 |
| SAUSA300_0942 | . | conserved hypothetical protein | 0.126085 | 2.0 |
| SAUSA300_0957 | . | conserved hypothetical protein | 0.000000 | 2.7 |
| SAUSA300_0986 | . | cytochrome D ubiquinol oxidase, subunit I | 0.000000 | 3.3 |
| SAUSA300_0987 | . | cytochrome D ubiquinol oxidase, subunit II | 0.000000 | 7.1 |
| SAUSA300_0991 | def | peptide deformylase | 0.000000 | 2.6 |
| SAUSA300_1009 | typA | GTP-binding protein | 0.000000 | 2.3 |
| SAUSA300_1065 | . | exfoliative toxin A | 0.000011 | 2.0 |
| SAUSA300_1066 | . | tRNA-Arg | 0.000044 | 4.5 |
| SAUSA300_1091 | pyrR | PyrR bifunctional protein | 0.000000 | 4.1 |
| SAUSA300_1092 | pyrP | uracil permease | 0.000000 | 17.0 |
| SAUSA300_1093 | pyrB | aspartate carbamoyltransferase | 0.000000 | 14.4 |
| SAUSA300_1094 | pyrC | dihydroorotase | 0.000000 | 14.6 |
| SAUSA300_1095 | carA | carbamoyl-phosphate synthase, small subunit | 0.000000 | 10.9 |
| SAUSA300_1096 | carB | carbamoyl-phosphate synthase, large subunit | 0.000000 | 5.9 |
| SAUSA300_1097 | pyrF | orotidine 5-phosphate decarboxylase | 0.000000 | 3.4 |
| SAUSA300_1098 | pyrE | orotate phosphoribosyltransferase | 0.000000 | 2.9 |
| SAUSA300_1178 | recA | recombinase A protein | 0.000000 | 8.4 |
| SAUSA300_1202 | . | conserved hypothetical protein | 0.138521 | 2.3 |
| SAUSA300_1237 | lexA | LexA repressor | 0.000000 | 3.4 |
| SAUSA300_1238 | . | conserved hypothetical protein | 0.000000 | 5.3 |
| SAUSA300_1242 | sbcD | exonuclease SbcD | 0.000000 | 6.9 |
| SAUSA300_1243 | sbcC | exonuclease SbcC | 0.000000 | 5.1 |
| SAUSA300_1250 | parE | DNA topoisomerase IV, subunit B | 0.000000 | 2.3 |
| SAUSA300_1251 | parC | DNA topoisomerase IV, subunit A | 0.000000 | 2.5 |
| SAUSA300_1259 | . | ImpB/MucB/SamB family protein | 0.000000 | 169.3 |
| SAUSA300_1261 | . | putative glutamyl aminopeptidase | 0.000000 | 6.4 |
| SAUSA300_1262 | trpE | anthranilate synthase component I | 0.000212 | 2.8 |
| SAUSA300_1282 | pstC | phosphate ABC transporter, permease protein PstC | 0.000006 | 6.5 |
| SAUSA300_1283 | pstS | phosphate ABC transporter, phosphate-binding protein PstS | 0.000000 | 9.9 |
| SAUSA300_1294 | . | conserved hypothetical protein | 0.079220 | 2.4 |
| SAUSA300_1300 | brnQ | branched-chain amino acid transport system II carrier protein | 0.000000 | 2.1 |
| SAUSA300_1328 | . | putative drug transporter | 0.000000 | 2.5 |
| SAUSA300_1329 | . | amino acid permease | 0.000000 | 2.4 |
| SAUSA300_1330 | ilvA | threonine dehydratase | 0.000000 | 2.8 |
| SAUSA300_1331 | ald | alanine dehydrogenase | 0.000000 | 4.7 |
| SAUSA300_1334 | . | putative membrane protein | 0.000000 | 2.3 |
| SAUSA300_1399 | . | phiSLT ORF110-like protein | 0.160655 | 2.5 |
| SAUSA300_1400 | . | phiSLT ORF92-like protein, uncharacterized phage protein | 0.052051 | 2.7 |
| SAUSA300_1405 | . | phiSLT ORF 101-like protein, terminase, small subunit | 0.369398 | 2.5 |
| SAUSA300_1406 | . | phiSLT ORF 104b-like protein | 0.363052 | 3.0 |
| SAUSA300_1422 | . | phiSLT ORF65-like protein | 0.717241 | 2.1 |
| SAUSA300_1427 | . | phiSLT ORF86-like protein | 0.254628 | 3.2 |
| SAUSA300_1428 | . | conserved hypothetical phage protein | 0.019330 | 13.9 |
| SAUSA300_1429 | . | phiSLT ORF53-like protein | 0.000000 | 420.2 |
| SAUSA300_1430 | . | phiSLT ORF 87-like protein, putative DNA-binding protein | 0.025884 | 5.3 |
| SAUSA300_1431 | . | phiSLT ORF71-like protein | 0.001281 | 17.5 |
| SAUSA300_1591 | apt | adenine phosphoribosyltransferase | 0.000000 | 2.1 |
| SAUSA300_1592 | recJ | single-stranded-DNA-specific exonuclease RecJ | 0.000000 | 2.2 |
| SAUSA300_1707 | . | conserved hypothetical protein | 0.000000 | 2.5 |
| SAUSA300_1709 | . | pseudogene | 0.000060 | 2.9 |
| SAUSA300_1726 | . | crcB family protein | 0.000040 | 2.1 |
| SAUSA300_1778 | . | tRNA-Asp | 0.000101 | 3.1 |
| SAUSA300_1779 | . | tRNA-Met | 0.000063 | 3.3 |
| SAUSA300_1794 | . | conserved hypothetical protein | 0.000194 | 7.4 |
| SAUSA300_1811 | . | tRNA-Leu | 0.005140 | 2.2 |
| SAUSA300_1814 | . | tRNA-Cys | 0.002291 | 3.2 |
| SAUSA300_1815 | . | tRNA-Gln | 0.000238 | 2.9 |
| SAUSA300_1816 | . | tRNA-His | 0.000001 | 2.6 |
| SAUSA300_1817 | . | tRNA-Trp | 0.000166 | 3.2 |
| SAUSA300_1821 | . | tRNA-Asp | 0.000255 | 2.7 |
| SAUSA300_1822 | . | tRNA-Met | 0.000171 | 2.4 |
| SAUSA300_1825 | . | tRNA-Ser | 0.001795 | 2.0 |
| SAUSA300_1833 | . | tRNA-Leu | 0.008973 | 2.2 |
| SAUSA300_1835 | . | tRNA-Thr | 0.000002 | 5.5 |
| SAUSA300_1836 | . | tRNA-Val | 0.231914 | 3.7 |
| SAUSA300_1840 | . | tRNA-Ile | 0.175364 | 4.2 |
| SAUSA300_1890 | . | staphopain A | 0.000000 | 2.4 |
| SAUSA300_1903 | . | conserved hypothetical protein | 0.000000 | 91.1 |
| SAUSA300_1915 | . | conserved hypothetical protein | 0.023763 | 3.0 |
| SAUSA300_1921 | . | truncated amidase | 0.000000 | 9.2 |
| SAUSA300_1922 | sak | staphylokinase precursor | 0.000000 | 8.6 |
| SAUSA300_1923 | . | autolysin | 0.000000 | 75.6 |
| SAUSA300_1924 | . | holin | 0.000000 | 90.0 |
| SAUSA300_1925 | . | phiPVL ORF17-like protein | 0.000000 | 56.5 |
| SAUSA300_1926 | . | phi77 ORF044-like protein | 0.000000 | 47.8 |
| SAUSA300_1927 | . | phi77 ORF109-like protein | 0.000000 | 77.8 |
| SAUSA300_1928 | . | phi77 ORF002-like protein, phage minor structural protein | 0.000000 | 40.8 |
| SAUSA300_1929 | . | phi77 ORF004-like protein, putative phage tail component | 0.000000 | 68.9 |
| SAUSA300_1930 | . | phi77 ORF001-like protein, phage tail tape measure protein | 0.000000 | 48.2 |
| SAUSA300_1931 | . | phi77 ORF100-like protein | 0.000000 | 146.5 |
| SAUSA300_1932 | . | conserved hypothetical phage protein | 0.000000 | 104.7 |
| SAUSA300_1933 | . | hypothetical phage protein | 0.000000 | 167.2 |
| SAUSA300_1934 | . | phi77 ORF020-like protein, phage major tail protein | 0.000000 | 154.8 |
| SAUSA300_1935 | . | phi77 ORF029-like protein | 0.000000 | 140.4 |
| SAUSA300_1936 | . | conserved hypothetical phage protein | 0.000000 | 146.9 |
| SAUSA300_1937 | . | phi77 ORF045-like protein | 0.000000 | 140.1 |
| SAUSA300_1938 | . | phi77 ORF006-like protein, putative capsid protein | 0.000000 | 152.7 |
| SAUSA300_1939 | . | phi77 ORF015-like protein, putative protease | 0.000000 | 173.1 |
| SAUSA300_1940 | . | phage portal protein | 0.000000 | 162.9 |
| SAUSA300_1941 | . | phi77 ORF003-like protein, phage terminase, large subunit | 0.000000 | 175.5 |
| SAUSA300_1942 | . | conserved hypothetical phage protein | 0.000000 | 251.6 |
| SAUSA300_1943 | . | phi77 ORF040-like protein | 0.000000 | 398.0 |
| SAUSA300_1944 | . | phi77 ORF026-like protein, putative phage transcriptional activator | 0.000000 | 89.0 |
| SAUSA300_1945 | . | phi77 ORF071-like protein | 0.000000 | 95.0 |
| SAUSA300_1946 | . | phiPVL ORF057-like protein, transcriptional activator RinB | 0.000000 | 104.7 |
| SAUSA300_1947 | . | phi77 ORF031-like protein | 0.000000 | 76.9 |
| SAUSA300_1948 | . | phi77 ORF069-like protein | 0.000000 | 99.9 |
| SAUSA300_1949 | dut | dUTP diphosphatase | 0.000000 | 101.8 |
| SAUSA300_1950 | . | conserved hypothetical phage protein | 0.000002 | 36.0 |
| SAUSA300_1951 | . | phiPVL ORF052-like protein | 0.000000 | 111.0 |
| SAUSA300_1952 | . | phiPV083 ORF027-like protein | 0.000000 | 190.6 |
| SAUSA300_1953 | . | phiPVL ORF051-like protein | 0.000000 | 119.3 |
| SAUSA300_1954 | . | phiPVL ORF050-like protein | 0.000000 | 171.3 |
| SAUSA300_1955 | . | putative endodeoxyribonuclease RusA | 0.000000 | 205.9 |
| SAUSA300_1956 | . | conserved hypothetical phage protein | 0.000000 | 201.6 |
| SAUSA300_1957 | . | phiPVL ORF046-like protein | 0.000000 | 180.5 |
| SAUSA300_1958 | . | Single-strand binding protein | 0.000000 | 251.9 |
| SAUSA300_1959 | . | phiPVL ORF044-like protein | 0.000000 | 195.2 |
| SAUSA300_1960 | . | putative phage-related DNA recombination protein | 0.000000 | 261.6 |
| SAUSA300_1961 | . | phiPVL ORF41-like protein | 0.000000 | 296.7 |
| SAUSA300_1962 | . | phiPVL ORF39-like protein | 0.000000 | 342.0 |
| SAUSA300_1963 | . | conserved hypothetical phage protein | 0.000000 | 430.8 |
| SAUSA300_1964 | . | conserved hypothetical phage protein | 0.000000 | 432.8 |
| SAUSA300_1966 | . | phi77 ORF014-like protein, phage anti-repressor protein | 0.000000 | 413.6 |
| SAUSA300_1967 | . | conserved hypothetical phage protein | 0.000000 | 4.1 |
| SAUSA300_1968 | . | putative phage transcriptional regulator | 0.000000 | 407.5 |
| SAUSA300_1969 | . | phi77 ORF011-like protein, phage transcriptional repressor | 0.000000 | 17.9 |
| SAUSA300_1970 | . | putative exonuclease | 0.000000 | 6.1 |
| SAUSA300_1971 | . | phi77 ORF017-like protein | 0.000000 | 3.1 |
| SAUSA300_1972 | int | integrase | 0.000000 | 5.1 |
| SAUSA300_1973 | . | truncated beta-hemolysin | 0.000000 | 12.9 |
| SAUSA300_1981 | . | phage terminase family protein | 0.034986 | 2.2 |
| SAUSA300_1982 | groL | 60 kDa chaperonin | 0.000000 | 2.4 |
| SAUSA300_1983 | groES | 10 kDa chaperonin | 0.000000 | 2.2 |
| SAUSA300_1997 | . | conserved hypothetical protein | 0.000000 | 4.6 |
| SAUSA300_1998 | . | putative membrane protein | 0.000000 | 4.1 |
| SAUSA300_2015 | rrfD | 5S ribosomal RNA | 0.213370 | 2.4 |
| SAUSA300_2019 | . | tRNA-Leu | 0.000000 | 4.5 |
| SAUSA300_2051 | . | conserved hypothetical protein | 0.000000 | 14.2 |
| SAUSA300_2070 | . | conserved hypothetical protein | 0.000000 | 2.2 |
| SAUSA300_2092 | dps | general stress protein 20U | 0.000000 | 2.9 |
| SAUSA300_2098 | arsR | transcriptional repressor, ArsR family | 0.000000 | 2.3 |
| SAUSA300_2099 | . | cation efflux family protein | 0.000000 | 2.2 |
| SAUSA300_2116 | . | tRNA-Lys | 0.001626 | 2.1 |
| SAUSA300_2117 | . | tRNA-Gln | 0.000171 | 3.6 |
| SAUSA300_2128 | . | putative drug transporter | 0.000000 | 3.4 |
| SAUSA300_2129 | . | putative hemolysin III | 0.000000 | 2.9 |
| SAUSA300_2130 | . | UTP-glucose-1-phosphate uridylyltransferase family protein | 0.000000 | 2.7 |
| SAUSA300_2131 | . | conserved hypothetical protein | 0.000000 | 4.0 |
| SAUSA300_2133 | . | transporter gate domain protein | 0.000000 | 2.9 |
| SAUSA300_2135 | . | iron compound ABC transporter, permease protein | 0.000002 | 2.0 |
| SAUSA300_2141 | . | . | 0.119035 | 2.1 |
| SAUSA300_2166 | alsS | alpha-acetolactate synthase | 0.000000 | 2.5 |
| SAUSA300_2171 | rpsI | 30S ribosomal protein S9 | 0.000000 | 2.6 |
| SAUSA300_2172 | rplM | 50S ribosomal protein L13 | 0.001283 | 2.0 |
| SAUSA300_2210 | glcU | probable glucose uptake protein | 0.000000 | 3.5 |
| SAUSA300_2218 | . | staphylococcal accessory regulator | 0.000004 | 2.0 |
| SAUSA300_2232 | . | acetyltransferase, GNAT family | 0.000000 | 2.5 |
| SAUSA300_2233 | . | BioY family protein | 0.000000 | 3.2 |
| SAUSA300_2249 | ssaA | secretory antigen precursor SsaA | 0.000000 | 13.2 |
| SAUSA300_2253 | ssaA | secretory antigen precursor SsaA | 0.000000 | 10.7 |
| SAUSA300_2255 | . | monooxygenase family protein | 0.000000 | 2.4 |
| SAUSA300_2282 | . | putative membrane protein | 0.000000 | 2.5 |
| SAUSA300_2291 | gltS | sodium/glutamate symporter | 0.000000 | 2.6 |
| SAUSA300_2305 | . | transposase, truncation | 0.188131 | 2.1 |
| SAUSA300_2306 | . | ABC transporter, ATP-binding protein | 0.000000 | 14.3 |
| SAUSA300_2307 | . | ABC transporter, permease protein | 0.000000 | 22.7 |
| SAUSA300_2320 | . | conserved hypothetical protein | 0.000000 | 2.0 |
| SAUSA300_2324 | . | PTS system, sucrose-specific IIBC component | 0.000000 | 4.6 |
| SAUSA300_2329 | gltT | proton/sodium-glutamate symport protein | 0.000000 | 2.8 |
| SAUSA300_2330 | . | conserved hypothetical protein | 0.000000 | 2.4 |
| SAUSA300_2343 | . | respiratory nitrate reductase, alpha subunit | 0.000003 | 2.3 |
| SAUSA300_2347 | nirR | nitrite reductase transcriptional regulator NirR | 0.003751 | 2.3 |
| SAUSA300_2348 | . | conserved hypothetical protein | 0.000001 | 2.3 |
| SAUSA300_2349 | . | formate/nitrite transporter family protein | 0.000000 | 3.3 |
| SAUSA300_2357 | . | ABC transporter, ATP-binding protein | 0.000000 | 2.8 |
| SAUSA300_2358 | . | ABC transporter, permease protein | 0.000000 | 2.5 |
| SAUSA300_2359 | . | amino acid ABC transporter, amino acid-binding protein | 0.000000 | 2.3 |
| SAUSA300_2372 | bioA | adenosylmethionine-8-amino-7-oxononanoate transaminase | 0.072100 | 2.2 |
| SAUSA300_2373 | bioD | dethiobiotin synthase | 0.008164 | 3.4 |
| SAUSA300_2385 | . | putative membrane protein | 0.000009 | 2.1 |
| SAUSA300_2387 | . | NAD dependent epimerase/dehydratase family protein | 0.000000 | 2.9 |
| SAUSA300_2395 | . | amino acid permease | 0.000001 | 2.2 |
| SAUSA300_2437 | sarT | staphylococcal accessory regulator T | 0.002378 | 2.4 |
| SAUSA300_2440 | fnbB | fibronectin binding protein B | 0.000000 | 2.3 |
| SAUSA300_2443 | gntK | gluconate kinase | 0.000000 | 5.9 |
| SAUSA300_2444 | gntR | gluconate operon transcriptional repressor | 0.000000 | 8.9 |
| SAUSA300_2453 | . | ABC transporter, ATP-binding protein | 0.000000 | 4.6 |
| SAUSA300_2454 | . | membrane spanning protein | 0.000000 | 4.7 |
| SAUSA300_2476 | ptsG | phosphotransferase system, glucose-specific IIABC component | 0.000000 | 12.9 |
| SAUSA300_2479 | cidA | Holin-like protein cidA | 0.000000 | 7.5 |
| SAUSA300_2482 | . | conserved hypothetical protein | 0.000048 | 2.6 |
| SAUSA300_2489 | . | antibiotic transport-associated protein-like protein | 0.000098 | 2.1 |
| SAUSA300_2503 | . | secretory antigen precursor SsaA | 0.000000 | 5.2 |
| SAUSA300_2504 | . | acyltransferase | 0.000000 | 2.6 |
| SAUSA300_2505 | . | acetyltransferase, GNAT family | 0.000000 | 10.0 |
| SAUSA300_2506 | isaA | immunodominant staphylococcal antigen A precursor | 0.000000 | 3.3 |
| SAUSA300_2507 | . | regulatory protein-like protein | 0.000099 | 2.9 |
| SAUSA300_2508 | . | conserved hypothetical protein | 0.078479 | 4.2 |
| SAUSA300_2511 | . | conserved hypothetical protein | 0.000000 | 2.4 |
| SAUSA300_2512 | . | glyoxalase family protein | 0.000105 | 2.0 |
| SAUSA300_2520 | . | transporter gate domain protein | 0.000000 | 2.6 |
| SAUSA300_2521 | . | conserved hypothetical protein | 0.047883 | 2.1 |
| SAUSA300_2526 | pyrD | dihydroorotate dehydrogenase | 0.000000 | 5.1 |
| SAUSA300_2527 | . | conserved hypothetical protein | 0.000000 | 3.6 |
| SAUSA300_2528 | . | conserved hypothetical protein | 0.000000 | 3.1 |
| SAUSA300_2535 | panE | 2-dehydropantoate 2-reductase | 0.000000 | 2.2 |
| SAUSA300_2537 | . | L-lactate dehydrogenase | 0.000000 | 2.1 |
| SAUSA300_2561 | phoB | alkaline phosphatase | 0.000000 | 9.6 |
| SAUSA300_2564 | estA | tributyrin esterase | 0.000000 | 2.2 |
| SAUSA300_2565 | clfB | clumping factor B | 0.000000 | 4.0 |
| SAUSA300_2575 | . | transcriptional antiterminator, BglG family | 0.000000 | 2.5 |
| SAUSA300_2596 | cap1C | capsular polysaccharide biosynthesis protein Cap1C | 0.000494 | 2.3 |
| SAUSA300_2597 | cap1B | capsular polysaccharide biosynthesis protein Cap1B | 0.000162 | 3.4 |
| SAUSA300_2598 | cap1A | capsular polysaccharide biosynthesis protein Cap1A | 0.000029 | 7.6 |
| SAUSA300_2600 | icaA | intercellular adhesion protein A | 0.013550 | 2.4 |
| SAUSA300_2611 | hisD | histidinol dehydrogenase hisD | 0.008713 | 2.5 |
| SAUSA300_2612 | hisG | ATP phosphoribosyltransferase hisG | 0.052851 | 3.0 |
| SAUSA300_2624 | . | putative membrane protein | 0.000000 | 2.6 |
| SAUSA300_2625 | . | transcriptional regulator, PadR family | 0.000002 | 3.1 |
| SAUSA300_2633 | . | ABC transporter, ATP-binding protein | 0.007556 | 2.3 |
| SAUSA300_2635 | . | conserved hypothetical protein | 0.000217 | 3.1 |
| SAUSA300_2637 | . | conserved hypothetical protein | 0.001986 | 2.3 |
| SAUSA300_2638 | . | conserved hypothetical protein | 0.000000 | 2.9 |
| SAUSA300_2639 | . | cold shock protein | 0.000061 | 2.3 |
| SAUSA300_2640 | . | putative transcriptional regulator | 0.000000 | 2.5 |
| SAUSA300_2641 | . | conserved hypothetical protein | 0.000000 | 2.5 |
| SAUSA300_2642 | . | conserved hypothetical protein | 0.000000 | 3.2 |
| SAUSA300_2644 | gidB | glucose-inhibited division protein B | 0.000014 | 2.3 |
| SAUSA300_2645 | gidA | glucose-inhibited division protein A | 0.000015 | 2.1 |
| SAUSA300_2646 | trmE | tRNA modification GTPase | 0.000000 | 2.3 |
| SAUSA300_2647 | rnpA | ribonuclease P protein component | 0.000036 | 2.5 |
| SAUSA300_2648 | rpmH | 50S ribosomal protein L34 | 0.000000 | 2.9 |

**Supplementary Table 2. Genes down-regulated by streptozotocin** (Green color, the genes down-regulated by floxuridine)

| **Gene ID** | **Name** | **Product** | **p-value** | **Ratio** |
| --- | --- | --- | --- | --- |
| SAUSA300_0063 | . | cyclic nucleotide-binding domain protein | 0.005018 | 0.44 |
| SAUSA300_0064 | arcD | arginine/oirnithine antiporter | 0.000001 | 0.45 |
| SAUSA300_0065 | arcA | arginine deiminase | 0.000000 | 0.46 |
| SAUSA300_0067 | . | universal stress protein family | 0.000000 | 0.17 |
| SAUSA300_0070 | . | putative lysophospholipase | 0.000000 | 0.20 |
| SAUSA300_0087 | . | . | 0.000000 | 0.33 |
| SAUSA300_0105 | . | peptidase, M20/M25/M40 family | 0.000000 | 0.39 |
| SAUSA300_0106 | . | putative drug transporter | 0.000000 | 0.29 |
| SAUSA300_0108 | . | antigen, 67 kDa | 0.000000 | 0.47 |
| SAUSA300_0111 | . | conserved hypothetical protein | 0.000000 | 0.26 |
| SAUSA300_0112 | lctP | L-lactate permease | 0.000000 | 0.42 |
| SAUSA300_0115 | sirC | iron compound ABC transporter, permease protein SirC | 0.000000 | 0.27 |
| SAUSA300_0129 | . | Acetoin(diacetyl) reductase | 0.000000 | 0.49 |
| SAUSA300_0156 | cap5E | capsular polysaccharide biosynthesis protein Cap5E | 0.000002 | 0.37 |
| SAUSA300_0157 | cap5F | capsular polysaccharide biosynthesis protein Cap5F | 0.000000 | 0.34 |
| SAUSA300_0158 | cap5G | capsular polysaccharide biosynthesis protein Cap5G | 0.000000 | 0.33 |
| SAUSA300_0159 | cap5H | capsular polysaccharide biosynthesis protein Cap5H | 0.000000 | 0.22 |
| SAUSA300_0160 | cap5I | capsular polysaccharide biosynthesis protein Cap5I | 0.000000 | 0.31 |
| SAUSA300_0161 | cap5J | capsular polysaccharide biosynthesis protein Cap5J | 0.000000 | 0.28 |
| SAUSA300_0162 | cap5K | capsular polysaccharide biosynthesis protein Cap5K | 0.000086 | 0.39 |
| SAUSA300_0163 | cap5L | capsular polysaccharide biosynthesis protein Cap5L | 0.000000 | 0.35 |
| SAUSA300_0164 | cap5M | capsular polysaccharide biosynthesis protein Cap5M | 0.000000 | 0.32 |
| SAUSA300_0165 | cap5N | capsular polysaccharide biosynthesis protein Cap5N | 0.000000 | 0.40 |
| SAUSA300_0166 | cap5O | capsular polysaccharide biosynthesis protein Cap5O | 0.000000 | 0.42 |
| SAUSA300_0170 | . | aldehyde dehydrogenase | 0.000000 | 0.42 |
| SAUSA300_0171 | . | cation efflux family protein | 0.000000 | 0.46 |
| SAUSA300_0172 | . | conserved hypothetical protein | 0.000000 | 0.18 |
| SAUSA300_0181 | . | non-ribosomal peptide synthetase | 0.000000 | 0.42 |
| SAUSA300_0182 | . | 4-phosphopantetheinyl transferase superfamily protein | 0.000000 | 0.35 |
| SAUSA300_0189 | entB | isochorismatase | 0.000000 | 0.43 |
| SAUSA300_0190 | ipdC | indole-3-pyruvate decarboxylase | 0.000000 | 0.37 |
| SAUSA300_0192 | . | conserved hypothetical protein | 0.000000 | 0.17 |
| SAUSA300_0193 | . | conserved hypothetical protein | 0.000000 | 0.13 |
| SAUSA300_0194 | . | sucrose-specific PTS tranporter protein | 0.000000 | 0.13 |
| SAUSA300_0195 | . | RpiR family transcriptional regulator | 0.000000 | 0.13 |
| SAUSA300_0210 | . | maltose ABC transporter, permease protein | 0.000000 | 0.42 |
| SAUSA300_0211 | . | maltose ABC transporter, permease protein | 0.000000 | 0.40 |
| SAUSA300_0212 | . | oxidoreductase, Gfo/Idh/MocA family | 0.000000 | 0.27 |
| SAUSA300_0213 | . | oxidoreductase, Gfo/Idh/MocA family | 0.000000 | 0.27 |
| SAUSA300_0214 | . | conserved hypothetical protein | 0.000000 | 0.27 |
| SAUSA300_0220 | pflB | formate acetyltransferase | 0.000000 | 0.36 |
| SAUSA300_0221 | pflA | pyruvate formate-lyase activating enzyme | 0.000000 | 0.29 |
| SAUSA300_0223 | . | conserved hypothetical protein | 0.003300 | 0.45 |
| SAUSA300_0225 | . | putative acyl-CoA acetyltransferase FadA | 0.000016 | 0.36 |
| SAUSA300_0226 | . | 3-hydroxyacyl-CoA dehydrogenase | 0.000000 | 0.18 |
| SAUSA300_0227 | fadD | acyl-CoA dehydrogenase FadD | 0.000000 | 0.10 |
| SAUSA300_0228 | fadE | acyl-CoA synthetase FadE | 0.000000 | 0.13 |
| SAUSA300_0229 | . | putative acyl-CoA transferase FadX | 0.000000 | 0.14 |
| SAUSA300_0235 | . | L-lactate dehydrogenase | 0.000000 | 0.42 |
| SAUSA300_0259 | . | PTS system, IIA component | 0.000469 | 0.49 |
| SAUSA300_0260 | bglA | 6-phospho-beta-glucosidase | 0.000000 | 0.22 |
| SAUSA300_0265 | . | putative ribose operon repressor | 0.000000 | 0.37 |
| SAUSA300_0278 | . | conserved hypothetical protein | 0.000000 | 0.22 |
| SAUSA300_0281 | . | conserved hypothetical protein | 0.025299 | 0.40 |
| SAUSA300_0282 | . | conserved hypothetical protein | 0.000003 | 0.50 |
| SAUSA300_0283 | . | essC protein | 0.000000 | 0.34 |
| SAUSA300_0284 | . | conserved hypothetical protein | 0.000022 | 0.27 |
| SAUSA300_0285 | . | conserved hypothetical protein | 0.000039 | 0.31 |
| SAUSA300_0286 | . | conserved hypothetical protein | 0.000000 | 0.28 |
| SAUSA300_0287 | . | conserved hypothetical protein | 0.000096 | 0.30 |
| SAUSA300_0308 | . | ABC transporter, permease protein | 0.000000 | 0.19 |
| SAUSA300_0309 | . | ABC transporter ATP-binding protein | 0.000000 | 0.20 |
| SAUSA300_0312 | . | indigoidine synthase family protein | 0.004294 | 0.46 |
| SAUSA300_0313 | . | putative nucleoside permease NupC | 0.000331 | 0.49 |
| SAUSA300_0320 | . | triacylglycerol lipase precursor | 0.000000 | 0.18 |
| SAUSA300_0329 | . | putative oxidoreductase | 0.000000 | 0.20 |
| SAUSA300_0330 | . | putative transport protein SgaT | 0.000000 | 0.09 |
| SAUSA300_0331 | . | conserved hypothetical protein | 0.000000 | 0.11 |
| SAUSA300_0332 | . | PTS system, IIA component | 0.000000 | 0.12 |
| SAUSA300_0333 | . | transcriptional antiterminator, BglG family | 0.000000 | 0.28 |
| SAUSA300_0338 | . | glyoxalase family protein | 0.000000 | 0.17 |
| SAUSA300_0339 | . | conserved hypothetical protein | 0.000000 | 0.18 |
| SAUSA300_0340 | . | NADH-dependent FMN reductase | 0.000000 | 0.22 |
| SAUSA300_0342 | . | conserved hypothetical protein | 0.021690 | 0.48 |
| SAUSA300_0372 | . | putative lipoprotein | 0.000000 | 0.27 |
| SAUSA300_0374 | . | putative membrane protein | 0.000000 | 0.20 |
| SAUSA300_0385 | . | conserved hypothetical protein | 0.000000 | 0.17 |
| SAUSA300_0391 | . | conserved hypothetical protein | 0.000015 | 0.43 |
| SAUSA300_0395 | . | exotoxin | 0.000000 | 0.25 |
| SAUSA300_0418 | . | staphylococcal tandem lipoprotein | 0.000015 | 0.44 |
| SAUSA300_0419 | . | staphylococcal tandem lipoprotein | 0.000000 | 0.44 |
| SAUSA300_0420 | . | conserved hypothetical protein | 0.000000 | 0.48 |
| SAUSA300_0421 | . | conserved hypothetical protein | 0.000000 | 0.44 |
| SAUSA300_0422 | . | conserved hypothetical protein | 0.000000 | 0.45 |
| SAUSA300_0471 | . | veg protein | 0.000000 | 0.48 |
| SAUSA300_0474 | . | putative endoribonuclease L-PSP | 0.000000 | 0.28 |
| SAUSA300_0475 | . | SpoVG protein | 0.000000 | 0.29 |
| SAUSA300_0476 | . | hypothetical protein | 0.025268 | 0.32 |
| SAUSA300_0491 | cysK | cysteine synthase A | 0.000000 | 0.45 |
| SAUSA300_0498 | . | tRNA-Ala | 0.023170 | 0.42 |
| SAUSA300_0537 | . | L-ribulokinase | 0.000000 | 0.41 |
| SAUSA300_0555 | . | putative hexulose-6-phosphate synthase | 0.000000 | 0.50 |
| SAUSA300_0556 | . | SIS domain protein | 0.000000 | 0.50 |
| SAUSA300_0573 | mvaD | diphosphomevalonate decarboxylase | 0.000000 | 0.33 |
| SAUSA300_0574 | . | phosphomevalonate kinase | 0.000000 | 0.43 |
| SAUSA300_0594 | adh | alcohol dehydrogenase | 0.000000 | 0.42 |
| SAUSA300_0602 | . | conserved hypothetical protein | 0.000000 | 0.25 |
| SAUSA300_0604 | . | hydrolase, alpha/beta hydrolase fold family | 0.000000 | 0.25 |
| SAUSA300_0605 | sarA | staphylococcal accessory regulator A | 0.000000 | 0.23 |
| SAUSA300_0609 | . | phage integrase family protein | 0.000000 | 0.19 |
| SAUSA300_0610 | . | putative Na+/H+ antiporter, MnhA component | 0.000000 | 0.29 |
| SAUSA300_0611 | . | putative Na+/H+ antiporter, MnhB component | 0.000000 | 0.31 |
| SAUSA300_0612 | . | putative Na+/H+ antiporter, MnhC component | 0.000014 | 0.44 |
| SAUSA300_0613 | . | putative Na+/H+ antiporter, MnhD component | 0.000000 | 0.37 |
| SAUSA300_0614 | . | putative Na+/H+ antiporter, MnhE component | 0.000421 | 0.38 |
| SAUSA300_0615 | . | putative Na+/H+ antiporter, MnhF component | 0.000153 | 0.43 |
| SAUSA300_0616 | . | putative Na+/H+ antiporter, MnhG component | 0.000072 | 0.47 |
| SAUSA300_0630 | . | ABC transporter, ATP-binding protein | 0.000000 | 0.24 |
| SAUSA300_0662 | . | acetyltransferase, GNAT family | 0.000010 | 0.32 |
| SAUSA300_0664 | . | conserved hypothetical protein | 0.000000 | 0.24 |
| SAUSA300_0665 | . | acetyltransferase, GNAT family | 0.000000 | 0.47 |
| SAUSA300_0666 | . | decarboxylase family protein | 0.000000 | 0.36 |
| SAUSA300_0667 | . | YaiI/YqxD family protein | 0.000000 | 0.30 |
| SAUSA300_0668 | . | conserved hypothetical protein | 0.000000 | 0.20 |
| SAUSA300_0688 | . | oxidoreductase, aldo/keto reductase family | 0.000000 | 0.26 |
| SAUSA300_0689 | . | glycosyl transferase, group 2 family protein | 0.000000 | 0.22 |
| SAUSA300_0711 | . | conserved hypothetical protein | 0.000000 | 0.23 |
| SAUSA300_0749 | . | conserved hypothetical protein | 0.000000 | 0.21 |
| SAUSA300_0750 | . | conserved hypothetical protein | 0.000000 | 0.25 |
| SAUSA300_0753 | . | conserved hypothetical protein | 0.000000 | 0.26 |
| SAUSA300_0770 | . | conserved hypothetical protein | 0.000000 | 0.29 |
| SAUSA300_0771 | . | acetyltransferase, GNAT family | 0.000000 | 0.48 |
| SAUSA300_0772 | clfA | clumping factor A | 0.000000 | 0.16 |
| SAUSA300_0778 | . | conserved hypothetical protein | 0.000000 | 0.40 |
| SAUSA300_0779 | . | conserved hypothetical protein | 0.000000 | 0.45 |
| SAUSA300_0781 | . | conserved hypothetical protein | 0.000000 | 0.28 |
| SAUSA300_0786 | . | OsmC/Ohr family protein | 0.000000 | 0.26 |
| SAUSA300_0794 | . | Toprim domain protein | 0.000000 | 0.31 |
| SAUSA300_0795 | . | putative thioredoxin | 0.000000 | 0.31 |
| SAUSA300_0816 | . | CsbD-like superfamily | 0.000000 | 0.23 |
| SAUSA300_0824 | . | conserved hypothetical protein | 0.000000 | 0.39 |
| SAUSA300_0893 | oppF | oligopeptide ABC transporter, ATP-binding protein | 0.030759 | 0.46 |
| SAUSA300_0935 | . | conserved hypothetical protein | 0.090836 | 0.45 |
| SAUSA300_0949 | sspC | cysteine protease | 0.000001 | 0.38 |
| SAUSA300_0950 | sspB | cysteine protease precursor | 0.000001 | 0.41 |
| SAUSA300_0951 | sspA | V8 protease | 0.000003 | 0.37 |
| SAUSA300_0960 | qoxD | quinol oxidase, subunit IV | 0.000000 | 0.43 |
| SAUSA300_0961 | qoxC | quinol oxidase, subunit III | 0.000000 | 0.45 |
| SAUSA300_0962 | qoxB | quinol oxidase, subunit I | 0.000000 | 0.46 |
| SAUSA300_0963 | qoxA | quinol oxidase, subunit II | 0.000000 | 0.44 |
| SAUSA300_0965 | folD | methylenetetrahydrofolate dehydrogenase/methenyltetrahydrofolate cyclohydrolase | 0.000000 | 0.41 |
| SAUSA300_0982 | . | conserved hypothetical protein | 0.000000 | 0.25 |
| SAUSA300_0998 | . | conserved hypothetical protein | 0.018871 | 0.40 |
| SAUSA300_0999 | potA | spermidine/putrescine ABC transporter, ATP-binding protein | 0.036839 | 0.45 |
| SAUSA300_1002 | potD | spermidine/putrescine ABC transporter, spermidine/putrescine-binding protein | 0.000000 | 0.23 |
| SAUSA300_1045 | uvrC | excinuclease ABC, C subunit | 0.000000 | 0.39 |
| SAUSA300_1047 | sdhA | succinate dehydrogenase, flavoprotein subunit | 0.000000 | 0.40 |
| SAUSA300_1048 | sdhB | succinate dehydrogenase, iron-sulfur protein | 0.000000 | 0.32 |
| SAUSA300_1052 | . | fibrinogen-binding protein | 0.000013 | 0.33 |
| SAUSA300_1053 | . | conserved hypothetical protein | 0.008115 | 0.33 |
| SAUSA300_1055 | efb | fibrinogen-binding protein | 0.000000 | 0.25 |
| SAUSA300_1056 | . | conserved hypothetical protein | 0.000002 | 0.30 |
| SAUSA300_1058 | . | alpha-hemolysin precursor | 0.000000 | 0.05 |
| SAUSA300_1064 | . | transporter, TRAP family | 0.000000 | 0.36 |
| SAUSA300_1107 | . | conserved hypothetical protein | 0.000000 | 0.19 |
| SAUSA300_1117 | rpmB | 50S ribosomal protein L28 | 0.000000 | 0.26 |
| SAUSA300_1121 | . | conserved hypothetical protein | 0.000000 | 0.30 |
| SAUSA300_1122 | plsX | fatty acid/phospholipid synthesis protein PlsX | 0.000000 | 0.37 |
| SAUSA300_1123 | fabD | malonyl CoA-acyl carrier protein transacylase | 0.000000 | 0.49 |
| SAUSA300_1138 | sucC | succinyl-CoA synthetase, beta subunit | 0.000000 | 0.48 |
| SAUSA300_1139 | sucD | succinyl-CoA synthetase, alpha subunit | 0.000000 | 0.40 |
| SAUSA300_1145 | xerC | tyrosine recombinase xerC | 0.000000 | 0.41 |
| SAUSA300_1146 | hslV | ATP-dependent protease hslV | 0.000000 | 0.43 |
| SAUSA300_1147 | hslU | heat shock protein HslVU, ATPase subunit HslU | 0.000000 | 0.46 |
| SAUSA300_1164 | truB | tRNA pseudouridine synthase B | 0.000000 | 0.37 |
| SAUSA300_1165 | ribF | riboflavin biosynthesis protein ribF | 0.000000 | 0.49 |
| SAUSA300_1179 | . | conserved hypothetical protein | 0.000000 | 0.45 |
| SAUSA300_1180 | . | conserved hypothetical protein | 0.000000 | 0.41 |
| SAUSA300_1182 | . | pyruvate ferredoxin oxidoreductase, alpha subunit | 0.000000 | 0.39 |
| SAUSA300_1191 | glpF | glycerol uptake facilitator | 0.000000 | 0.27 |
| SAUSA300_1192 | glpK | glycerol kinase | 0.000000 | 0.43 |
| SAUSA300_1193 | glpD | glycerol-3-phosphate dehydrogenase | 0.000026 | 0.49 |
| SAUSA300_1205 | . | conserved hypothetical protein | 0.046958 | 0.33 |
| SAUSA300_1225 | . | aspartate kinase | 0.000001 | 0.39 |
| SAUSA300_1226 | . | homoserine dehydrogenase | 0.000000 | 0.10 |
| SAUSA300_1227 | thrC | threonine synthase | 0.000000 | 0.13 |
| SAUSA300_1228 | thrB | homoserine kinase | 0.000000 | 0.13 |
| SAUSA300_1229 | . | hydrolase, haloacid dehalogenase-like family | 0.000000 | 0.34 |
| SAUSA300_1232 | . | catalase | 0.000000 | 0.27 |
| SAUSA300_1235 | guaC | guanosine monophosphate reductase | 0.052307 | 0.43 |
| SAUSA300_1236 | . | conserved hypothetical protein | 0.000000 | 0.31 |
| SAUSA300_1260 | . | prephenate dehydrogenase | 0.000000 | 0.15 |
| SAUSA300_1269 | femA | methicillin resistance protein FemA | 0.000000 | 0.36 |
| SAUSA300_1272 | . | conserved hypothetical protein | 0.000000 | 0.34 |
| SAUSA300_1273 | opp-2F | oligopeptide permease, ATP-binding protein | 0.000000 | 0.27 |
| SAUSA300_1290 | dapD | tetrahydrodipicolinate acetyltransferase | 0.000033 | 0.45 |
| SAUSA300_1297 | . | conserved hypothetical protein | 0.000000 | 0.45 |
| SAUSA300_1304 | . | conserved hypothetical protein | 0.000000 | 0.44 |
| SAUSA300_1305 | sucB | 2-oxoglutarate dehydrogenase, E2 component, dihydrolipoamide succinyltransferase | 0.000000 | 0.10 |
| SAUSA300_1306 | sucA | 2-oxoglutarate dehydrogenase, E1 component | 0.000000 | 0.12 |
| SAUSA300_1307 | arlS | sensor histidine kinase protein | 0.000000 | 0.29 |
| SAUSA300_1308 | arlR | DNA-binding response regulator | 0.000000 | 0.24 |
| SAUSA300_1312 | . | acetyltransferase, GNAT family | 0.000000 | 0.41 |
| SAUSA300_1317 | msrA | methionine-S-sulfoxide reductase | 0.000000 | 0.44 |
| SAUSA300_1319 | folA | dihydrofolate reductase | 0.000000 | 0.45 |
| SAUSA300_1320 | thyA | thymidylate synthase | 0.000000 | 0.36 |
| SAUSA300_1325 | . | conserved hypothetical protein | 0.027342 | 0.46 |
| SAUSA300_1327 | . | cell surface protein | 0.000000 | 0.15 |
| SAUSA300_1337 | . | conserved hypothetical protein | 0.000000 | 0.43 |
| SAUSA300_1338 | . | conserved hypothetical protein | 0.000000 | 0.32 |
| SAUSA300_1339 | . | conserved hypothetical protein | 0.000000 | 0.40 |
| SAUSA300_1340 | recU | recombination protein U | 0.000000 | 0.32 |
| SAUSA300_1362 | hup | DNA-binding protein HU | 0.000000 | 0.30 |
| SAUSA300_1365 | rpsA | 30S ribosomal protein S1 | 0.000000 | 0.32 |
| SAUSA300_1370 | ebpS | cell surface elastin binding protein | 0.000000 | 0.27 |
| SAUSA300_1371 | recQ | ATP-dependent DNA helicase RecQ | 0.000000 | 0.29 |
| SAUSA300_1372 | . | conserved hypothetical protein | 0.000000 | 0.49 |
| SAUSA300_1374 | . | conserved hypothetical protein | 0.000000 | 0.46 |
| SAUSA300_1377 | . | conserved hypothetical protein | 0.024208 | 0.31 |
| SAUSA300_1381 | lukF-PV | Panton-Valentine leukocidin, LukF-PV | 0.000000 | 0.28 |
| SAUSA300_1382 | lukS-PV | Panton-Valentine leukocidin, LukS-PV | 0.000000 | 0.28 |
| SAUSA300_1390 | . | phiSLT ORF96-like protein | 0.059243 | 0.43 |
| SAUSA300_1391 | . | phiSLT ORF527-like protein | 0.000003 | 0.35 |
| SAUSA300_1392 | . | phiSLT ORF191-like protein | 0.000000 | 0.19 |
| SAUSA300_1393 | . | phiSLT ORF2067-like protein, phage tail tape measure protein | 0.000000 | 0.24 |
| SAUSA300_1394 | . | conserved hypothetical phage protein | 0.371593 | 0.38 |
| SAUSA300_1395 | . | phiSLT ORF116b-like protein | 0.350926 | 0.42 |
| SAUSA300_1396 | . | phiSLT ORF151-like protein, major tail protein | 0.000000 | 0.32 |
| SAUSA300_1397 | . | phiSLT ORF213-like protein, major tail protein | 0.003158 | 0.49 |
| SAUSA300_1407 | . | phi SLT ORF 145-like protein, phage transcriptional regulator | 0.000000 | 0.24 |
| SAUSA300_1408 | . | phage helicase | 0.000000 | 0.23 |
| SAUSA300_1409 | . | conserved hypothetical phage protein | 0.000004 | 0.24 |
| SAUSA300_1410 | . | virulence-associated protein E | 0.000000 | 0.39 |
| SAUSA300_1415 | . | phiSLT ORF 77-like protein | 0.039490 | 0.20 |
| SAUSA300_1417 | . | phiSLT ORF 175-like protein | 0.208689 | 0.23 |
| SAUSA300_1432 | . | phiSLT ORF78-like protein | 0.000000 | 0.38 |
| SAUSA300_1436 | . | phiSLT ORF144-like protein, putative lipoprotein | 0.000003 | 0.47 |
| SAUSA300_1437 | . | phiSLT ORF204-like protein | 0.000000 | 0.43 |
| SAUSA300_1439 | . | . | 0.000012 | 0.49 |
| SAUSA300_1450 | . | oxidoreductase, aldo/keto reductase family | 0.000000 | 0.45 |
| SAUSA300_1456 | . | alpha glucosidase | 0.000000 | 0.33 |
| SAUSA300_1460 | . | peptidase, M20/M25/M40 family | 0.000000 | 0.48 |
| SAUSA300_1464 | . | 2-oxoisovalerate dehydrogenase, E2 component, dihydrolipoamide acetyltransferase | 0.000000 | 0.35 |
| SAUSA300_1465 | . | 2-oxoisovalerate dehydrogenase, E1 component, beta subunit | 0.000000 | 0.33 |
| SAUSA300_1466 | . | 2-oxoisovalerate dehydrogenase, E1 component, alpha subunit | 0.000000 | 0.36 |
| SAUSA300_1467 | lpdA | 2-oxoisovalerate dehydrogenase, E3 component, lipoamide dehydrogenase | 0.000000 | 0.38 |
| SAUSA300_1479 | . | conserved hypothetical protein | 0.000000 | 0.18 |
| SAUSA300_1480 | . | putative traG membrane protein | 0.000000 | 0.15 |
| SAUSA300_1481 | . | putative membrane protein | 0.000000 | 0.41 |
| SAUSA300_1492 | . | putative lipoprotein | 0.000000 | 0.45 |
| SAUSA300_1498 | gcvT | aminomethyltransferase (glycine cleavage system T protein) | 0.000000 | 0.41 |
| SAUSA300_1499 | aroK | shikimate kinase | 0.000003 | 0.36 |
| SAUSA300_1502 | . | putative competence protein ComGC | 0.285816 | 0.46 |
| SAUSA300_1505 | . | conserved hypothetical protein | 0.000000 | 0.37 |
| SAUSA300_1506 | . | conserved hypothetical protein | 0.000000 | 0.38 |
| SAUSA300_1507 | glk | glucokinase | 0.000000 | 0.38 |
| SAUSA300_1508 | . | conserved hypothetical protein | 0.000000 | 0.39 |
| SAUSA300_1509 | . | peptidase, rhomboid family | 0.000000 | 0.45 |
| SAUSA300_1513 | . | superoxide dismutase (Mn/Fe family) | 0.000000 | 0.24 |
| SAUSA300_1527 | era | GTP-binding protein Era | 0.000000 | 0.43 |
| SAUSA300_1528 | cdd | cytidine deaminase | 0.000000 | 0.44 |
| SAUSA300_1529 | dgkA | diacylglycerol kinase | 0.000000 | 0.44 |
| SAUSA300_1530 | . | conserved hypothetical protein | 0.000000 | 0.42 |
| SAUSA300_1531 | phoH | phosphate starvation-induced protein, PhoH family | 0.000000 | 0.46 |
| SAUSA300_1533 | . | conserved hypothetical protein | 0.000000 | 0.49 |
| SAUSA300_1534 | . | conserved hypothetical protein | 0.000000 | 0.42 |
| SAUSA300_1561 | . | putative membrane protein | 0.000000 | 0.28 |
| SAUSA300_1562 | . | LamB/YcsF family protein | 0.000000 | 0.31 |
| SAUSA300_1563 | accC | acetyl-CoA carboxylase, biotin carboxylase | 0.000000 | 0.33 |
| SAUSA300_1564 | accB | acetyl-CoA carboxylase, biotin carboxyl carrier protein | 0.001284 | 0.37 |
| SAUSA300_1565 | . | putative urea amidolyase | 0.000000 | 0.32 |
| SAUSA300_1566 | . | conserved hypothetical protein | 0.000000 | 0.33 |
| SAUSA300_1581 | . | conserved hypothetical protein | 0.000000 | 0.17 |
| SAUSA300_1582 | . | conserved hypothetical protein | 0.000000 | 0.17 |
| SAUSA300_1610 | folC | folylpolyglutamate synthase | 0.000000 | 0.32 |
| SAUSA300_1611 | valS | valyl-tRNA synthetase | 0.000000 | 0.31 |
| SAUSA300_1613 | . | putative abrB protein | 0.000000 | 0.45 |
| SAUSA300_1628 | lysP | lysine-specific permease | 0.000000 | 0.28 |
| SAUSA300_1629 | thrS | threonyl-tRNA synthetase | 0.000000 | 0.18 |
| SAUSA300_1633 | gap | glyceraldehyde-3-phosphate dehydrogenase, type I | 0.000000 | 0.06 |
| SAUSA300_1638 | phoR | sensory box histidine kinase PhoR | 0.000000 | 0.41 |
| SAUSA300_1640 | icd | isocitrate dehydrogenase, NADP-dependent | 0.000000 | 0.39 |
| SAUSA300_1641 | gltA | citrate synthase II | 0.000000 | 0.44 |
| SAUSA300_1653 | . | conserved hypothetical protein | 0.000000 | 0.17 |
| SAUSA300_1655 | ald | alanine dehydrogenase | 0.000000 | 0.21 |
| SAUSA300_1656 | . | universal stress protein family | 0.000000 | 0.42 |
| SAUSA300_1667 | . | putative glycerophosphoryl diester phosphodiesterase | 0.000000 | 0.50 |
| SAUSA300_1679 | acsA | acetyl-coenzyme A synthetase | 0.000000 | 0.21 |
| SAUSA300_1680 | acuA | acetoin utilization protein AcuA | 0.000001 | 0.36 |
| SAUSA300_1681 | acuC | acetoin utilization protein AcuC | 0.000000 | 0.29 |
| SAUSA300_1684 | . | conserved hypothetical protein | 0.000000 | 0.21 |
| SAUSA300_1685 | . | conserved hypothetical protein | 0.000000 | 0.23 |
| SAUSA300_1687 | . | FtsK/SpoIIIE family protein | 0.000000 | 0.42 |
| SAUSA300_1688 | . | phenylalanyl-tRNA synthetase (beta subunit) | 0.000000 | 0.46 |
| SAUSA300_1690 | . | putative thioredoxin | 0.000000 | 0.31 |
| SAUSA300_1698 | . | conserved hypothetical protein | 0.000000 | 0.23 |
| SAUSA300_1708 | rot | staphylococcal accessory regulator Rot | 0.000000 | 0.48 |
| SAUSA300_1711 | putA | proline dehydrogenase | 0.000000 | 0.35 |
| SAUSA300_1712 | ribH | riboflavin synthase, beta subunit | 0.000119 | 0.14 |
| SAUSA300_1713 | ribBA | riboflavin biosynthesis protein | 0.000003 | 0.11 |
| SAUSA300_1714 | ribE | riboflavin synthase, alpha subunit | 0.000001 | 0.10 |
| SAUSA300_1715 | ribD | riboflavin biosynthesis protein | 0.000000 | 0.09 |
| SAUSA300_1716 | . | conserved hypothetical protein | 0.000000 | 0.30 |
| SAUSA300_1728 | . | oxidoreductase, aldo/keto reductase family | 0.000000 | 0.45 |
| SAUSA300_1731 | pckA | phosphoenolpyruvate carboxykinase (ATP) | 0.000000 | 0.09 |
| SAUSA300_1735 | menC | O-succinylbenzoic acid synthetase | 0.000000 | 0.49 |
| SAUSA300_1739 | . | conserved hypothetical protein | 0.000000 | 0.12 |
| SAUSA300_1740 | . | conserved hypothetical protein | 0.000000 | 0.11 |
| SAUSA300_1753 | splF | serine protease SplF | 0.000000 | 0.04 |
| SAUSA300_1754 | splE | serine protease SplE | 0.000000 | 0.04 |
| SAUSA300_1755 | splD | serine protease SplD | 0.000000 | 0.03 |
| SAUSA300_1756 | splC | serine protease SplC | 0.000000 | 0.04 |
| SAUSA300_1757 | splB | serine protease SplB | 0.000000 | 0.04 |
| SAUSA300_1758 | splA | serine protease SplA | 0.000000 | 0.04 |
| SAUSA300_1759 | . | conserved hypothetical protein | 0.008062 | 0.20 |
| SAUSA300_1760 | epiG | lantibiotic epidermin immunity protein F | 0.000000 | 0.05 |
| SAUSA300_1761 | epiE | lantibiotic epidermin immunity protein F | 0.000000 | 0.05 |
| SAUSA300_1762 | epiF | lantibiotic epidermin immunity protein F | 0.000000 | 0.05 |
| SAUSA300_1763 | epiP | lantibiotic epidermin leader peptide processing serine protease EpiP | 0.000000 | 0.06 |
| SAUSA300_1770 | . | conserved hypothetical protein | 0.000000 | 0.24 |
| SAUSA300_1788 | . | conserved hypothetical protein | 0.000000 | 0.20 |
| SAUSA300_1801 | fumC | fumarate hydratase, class II | 0.000000 | 0.46 |
| SAUSA300_1803 | . | conserved hypothetical protein | 0.000000 | 0.20 |
| SAUSA300_1804 | . | conserved hypothetical protein | 0.000000 | 0.19 |
| SAUSA300_1854 | . | regulatory protein RecX | 0.000000 | 0.49 |
| SAUSA300_1856 | . | conserved hypothetical protein | 0.000000 | 0.23 |
| SAUSA300_1861 | . | conserved hypothetical protein | 0.000007 | 0.47 |
| SAUSA300_1862 | . | conserved hypothetical protein | 0.000000 | 0.27 |
| SAUSA300_1863 | . | conserved hypothetical protein | 0.000000 | 0.34 |
| SAUSA300_1864 | . | putative membrane protein | 0.000000 | 0.26 |
| SAUSA300_1897 | . | sodium-dependent transporter | 0.000000 | 0.42 |
| SAUSA300_1898 | . | conserved hypothetical protein | 0.000000 | 0.23 |
| SAUSA300_1901 | aldA2 | aldehyde dehydrogenase | 0.000000 | 0.48 |
| SAUSA300_1904 | . | conserved hypothetical protein | 0.000001 | 0.30 |
| SAUSA300_1918 | . | truncated beta-hemolysin | 0.002105 | 0.44 |
| SAUSA300_1965 | . | conserved hypothetical phage protein | 0.000000 | 0.38 |
| SAUSA300_1976 | . | probable succinyl-diaminopimelate desuccinylase | 0.000000 | 0.10 |
| SAUSA300_1980 | . | acetyltransferase, GNAT family | 0.000000 | 0.30 |
| SAUSA300_1986 | . | nitroreductase family protein | 0.000000 | 0.47 |
| SAUSA300_1988 | . | delta-hemolysin precursor | 0.000000 | 0.30 |
| SAUSA300_1989 | agrB | accessory gene regulator protein B | 0.000000 | 0.09 |
| SAUSA300_1990 | agrD | accessory gene regulator protein D | 0.000000 | 0.10 |
| SAUSA300_1991 | agrC | accessory gene regulator protein C | 0.000000 | 0.10 |
| SAUSA300_1992 | agrA | accessory gene regulator protein A | 0.000000 | 0.09 |
| SAUSA300_2006 | ilvD | dihydroxy-acid dehydratase | 0.001339 | 0.46 |
| SAUSA300_2007 | ilvB | acetolactate synthase, large subunit | 0.000000 | 0.39 |
| SAUSA300_2008 | ilvN | acetolactate synthase, small subunit | 0.004132 | 0.34 |
| SAUSA300_2009 | ilvC | ketol-acid reductoisomerase | 0.000000 | 0.28 |
| SAUSA300_2010 | leuA | 2-isopropylmalate synthase | 0.000000 | 0.26 |
| SAUSA300_2011 | leuB | 3-isopropylmalate dehydrogenase | 0.000000 | 0.20 |
| SAUSA300_2012 | leuC | 3-isopropylmalate dehydratase, large subunit | 0.000000 | 0.26 |
| SAUSA300_2013 | leuD | 3-isopropylmalate dehydratase, small subunit | 0.000000 | 0.23 |
| SAUSA300_2014 | ilvA | threonine dehydratase | 0.000000 | 0.25 |
| SAUSA300_2022 | rpoF | RNA polymerase sigma-37 factor | 0.000000 | 0.45 |
| SAUSA300_2023 | rsbW | anti-sigma-B factor, serine-protein kinase | 0.000000 | 0.38 |
| SAUSA300_2024 | rsbV | anti-sigma-B factor, antagonist | 0.000000 | 0.33 |
| SAUSA300_2036 | kdpE | DNA-binding response regulator, KdpE | 0.000004 | 0.48 |
| SAUSA300_2041 | . | conserved hypothetical protein | 0.000000 | 0.19 |
| SAUSA300_2052 | . | single-stranded DNA- binding protein family | 0.000000 | 0.43 |
| SAUSA300_2055 | murA | UDP-N-acetylglucosamine 1-carboxyvinyltransferase 1 | 0.000000 | 0.48 |
| SAUSA300_2056 | . | conserved hypothetical protein | 0.000000 | 0.24 |
| SAUSA300_2065 | . | UDP-N-acetylglucosamine 2-epimerase | 0.000000 | 0.36 |
| SAUSA300_2066 | upp | uracil phosphoribosyltransferase | 0.000000 | 0.40 |
| SAUSA300_2067 | glyA | serine hydroxymethyltransferase | 0.000000 | 0.39 |
| SAUSA300_2068 | . | conserved hypothetical protein | 0.000000 | 0.43 |
| SAUSA300_2076 | . | aldehyde dehydrogenase family protein | 0.000000 | 0.33 |
| SAUSA300_2089 | pdp | pyrimidine nucleoside phosphorylase | 0.000000 | 0.42 |
| SAUSA300_2097 | . | conserved hypothetical protein | 0.000000 | 0.25 |
| SAUSA300_2104 | glmS | glucosamine--fructose-6-phosphate aminotransferase (isomerizing) | 0.000000 | 0.15 |
| SAUSA300_2106 | . | putative transcriptional regulator | 0.000000 | 0.24 |
| SAUSA300_2107 | mtlA | PTS system, mannitol specific IIA component | 0.000000 | 0.19 |
| SAUSA300_2108 | mtlD | Mannitol-1-phosphate 5-dehydrogenase | 0.000000 | 0.18 |
| SAUSA300_2114 | rocF | arginase | 0.000000 | 0.35 |
| SAUSA300_2126 | . | drug resistance transporter, EmrB/QacA subfamily | 0.000000 | 0.36 |
| SAUSA300_2132 | . | conserved hypothetical protein | 0.000000 | 0.23 |
| SAUSA300_2137 | . | conserved hypothetical protein | 0.000000 | 0.28 |
| SAUSA300_2138 | . | conserved hypothetical protein | 0.000000 | 0.29 |
| SAUSA300_2142 | asp23 | alkaline shock protein 23 | 0.000000 | 0.18 |
| SAUSA300_2143 | . | conserved hypothetical protein | 0.000000 | 0.18 |
| SAUSA300_2144 | . | conserved hypothetical protein | 0.000000 | 0.18 |
| SAUSA300_2145 | . | glycine betaine transporter | 0.000000 | 0.11 |
| SAUSA300_2150 | lacE | PTS system, lactose-specific IIBC component | 0.000065 | 0.39 |
| SAUSA300_2151 | lacF | PTS system, lactose-specific IIA component | 0.140601 | 0.44 |
| SAUSA300_2152 | lacD | tagatose 1,6-diphosphate aldolase | 0.000045 | 0.20 |
| SAUSA300_2153 | lacC | tagatose-6-phosphate kinase | 0.014168 | 0.44 |
| SAUSA300_2160 | . | transcriptional regulator, MerR family | 0.000046 | 0.49 |
| SAUSA300_2164 | . | conserved hypothetical protein | 0.000000 | 0.03 |
| SAUSA300_2168 | . | conserved hypothetical protein | 0.000004 | 0.46 |
| SAUSA300_2169 | . | conserved hypothetical protein | 0.000000 | 0.43 |
| SAUSA300_2236 | . | conserved hypothetical protein | 0.000000 | 0.40 |
| SAUSA300_2245 | . | staphylococcal accessory regulator R | 0.000000 | 0.34 |
| SAUSA300_2251 | . | dehydrogenase family protein | 0.000000 | 0.45 |
| SAUSA300_2257 | . | conserved hypothetical protein | 0.000000 | 0.27 |
| SAUSA300_2258 | . | formate dehydrogenase, alpha subunit | 0.000000 | 0.26 |
| SAUSA300_2260 | . | inositol monophosphatase family protein | 0.000000 | 0.31 |
| SAUSA300_2270 | glvC | PTS system, arbutin-like IIBC component | 0.000000 | 0.10 |
| SAUSA300_2275 | . | oxidoreductase, short chain dehydrogenase/reductase family | 0.000000 | 0.11 |
| SAUSA300_2277 | hutI | imidazolonepropionase | 0.000000 | 0.25 |
| SAUSA300_2278 | hutU | urocanate hydratase | 0.000000 | 0.27 |
| SAUSA300_2281 | hutG | formimidoylglutamase | 0.000000 | 0.18 |
| SAUSA300_2315 | . | putative lipoprotein | 0.000000 | 0.25 |
| SAUSA300_2327 | . | conserved hypothetical protein | 0.000000 | 0.24 |
| SAUSA300_2337 | . | transcriptional regulator, DegU family | 0.000000 | 0.44 |
| SAUSA300_2338 | . | sensor histidine kinase | 0.000000 | 0.49 |
| SAUSA300_2365 | hlgA | gamma-hemolysin component A | 0.000458 | 0.27 |
| SAUSA300_2366 | hlgC | gamma-hemolysin component C | 0.002813 | 0.46 |
| SAUSA300_2376 | . | conserved hypothetical protein | 0.000000 | 0.35 |
| SAUSA300_2383 | . | amino acid permease | 0.000000 | 0.38 |
| SAUSA300_2396 | pnbA | para-nitrobenzyl esterase | 0.000000 | 0.25 |
| SAUSA300_2398 | . | putative membrane protein | 0.000000 | 0.20 |
| SAUSA300_2399 | . | ABC transporter, ATP-binding protein | 0.000000 | 0.41 |
| SAUSA300_2415 | . | conserved hypothetical protein | 0.000000 | 0.19 |
| SAUSA300_2416 | . | glucose 1-dehydrogenase-like protein | 0.000000 | 0.14 |
| SAUSA300_2418 | . | conserved hypothetical protein | 0.000000 | 0.15 |
| SAUSA300_2447 | . | conserved hypothetical protein | 0.000000 | 0.32 |
| SAUSA300_2457 | . | phospholipase/carboxylesterase family protein | 0.000000 | 0.45 |
| SAUSA300_2458 | . | glyoxylase family protein | 0.000259 | 0.47 |
| SAUSA300_2464 | . | hydrolase, haloacid dehalogenase-like family | 0.000000 | 0.39 |
| SAUSA300_2477 | cidC | pyruvate oxidase | 0.000000 | 0.24 |
| SAUSA300_2478 | cidB | Holin-like protein cidB | 0.000000 | 0.19 |
| SAUSA300_2486 | . | putative ATP-dependent Clp proteinase | 0.000000 | 0.13 |
| SAUSA300_2498 | crtN | squalene synthase | 0.000000 | 0.14 |
| SAUSA300_2499 | crtM | squalene desaturase | 0.000000 | 0.11 |
| SAUSA300_2500 | . | glycosyl transferase | 0.000000 | 0.11 |
| SAUSA300_2501 | . | phytoene dehydrogenase | 0.000000 | 0.16 |
| SAUSA300_2502 | . | conserved hypothetical protein | 0.000000 | 0.20 |
| SAUSA300_2517 | . | amidohydrolase family protein | 0.000000 | 0.28 |
| SAUSA300_2518 | . | hydrolase family protein | 0.000000 | 0.17 |
| SAUSA300_2525 | . | conserved hypothetical protein | 0.000000 | 0.26 |
| SAUSA300_2543 | . | conserved hypothetical protein | 0.000000 | 0.22 |
| SAUSA300_2548 | . | conserved hypothetical protein | 0.000000 | 0.44 |
| SAUSA300_2567 | arcC | carbamate kinase | 0.000003 | 0.47 |
| SAUSA300_2568 | arcD | arginine/ornithine antiporter | 0.000008 | 0.35 |
| SAUSA300_2569 | arcB | ornithine carbamoyltransferase | 0.000180 | 0.41 |
| SAUSA300_2570 | arcA | arginine deiminase | 0.000120 | 0.35 |
| SAUSA300_2573 | isaB | immunodominant antigen B | 0.000000 | 0.42 |
| SAUSA300_2581 | . | putative surface anchored protein | 0.000000 | 0.33 |
| SAUSA300_2582 | . | conserved hypothetical protein | 0.000000 | 0.14 |
| SAUSA300_2583 | . | putative glycosyl transferase | 0.000000 | 0.13 |
| SAUSA300_2584 | . | preprotein translocase, secA protein | 0.000000 | 0.12 |
| SAUSA300_2585 | . | accessory secretory protein Asp3 | 0.000000 | 0.36 |
| SAUSA300_2586 | . | accessory secretory protein Asp2 | 0.000000 | 0.39 |
| SAUSA300_2592 | . | conserved hypothetical protein | 0.000000 | 0.34 |
| SAUSA300_2593 | . | conserved hypothetical protein | 0.000000 | 0.41 |
| SAUSA300_2603 | lip | triacylglycerol lipase precursor | 0.000000 | 0.16 |
| SAUSA300_2605 | hisIE | histidine biosynthesis bifunctional protein hisIE | 0.000151 | 0.43 |
| SAUSA300_2606 | hisF | imidazole glycerol phosphate synthase subunit hisF | 0.003655 | 0.47 |
| SAUSA300_2607 | hisA | phosphoribosylformimino-5-aminoimidazole carboxamide ribotide isomerase hisA | 0.042514 | 0.41 |
| SAUSA300_2620 | . | conserved hypothetical protein | 0.000000 | 0.30 |
| SAUSA300_2626 | . | conserved hypothetical protein | 0.000000 | 0.46 |
| SAUSA300_2629 | . | conserved hypothetical protein | 0.000000 | 0.28 |
| SAUSA300_2632 | . | putative membrane protein | 0.000000 | 0.20 |

**Supplementary Table 3. Genes up-regulated by floxuridine** (Pink color, the genes up-regulated by streptozotocin)

| **Gene ID** | **Name** | **Product** | **p-value** | **Ratio** |
| --- | --- | --- | --- | --- |
| SAUSA300_0001 | dnaA | chromosomal replication initiator protein DnaA | 0.000000 | 2.7 |
| SAUSA300_0002 | dnaN | DNA polymerase III, beta subunit | 0.000000 | 3.4 |
| SAUSA300_0005 | gyrB | DNA gyrase, B subunit | 0.000000 | 2.0 |
| SAUSA300_0006 | gyrA | DNA gyrase, A subunit | 0.000000 | 2.3 |
| SAUSA300_0007 | . | conserved hypothetical protein | 0.000000 | 3.3 |
| SAUSA300_0010 | . | putative membrane protein | 0.000000 | 2.3 |
| SAUSA300_0011 | . | conserved hypothetical protein | 0.000000 | 2.8 |
| SAUSA300_0017 | purA | adenylosuccinate synthetase | 0.000007 | 3.2 |
| SAUSA300_0019 | . | tRNA-Asp | 0.000026 | 3.6 |
| SAUSA300_0025 | . | 5-nucleotidase family protein | 0.000000 | 2.5 |
| SAUSA300_0034 | . | IS1272, transposase | 0.000001 | 3.8 |
| SAUSA300_0036 | . | conserved hypothetical protein | 0.000356 | 15.2 |
| SAUSA300_0037 | ccrB | cassette chromosome recombinase B | 0.000000 | 7.9 |
| SAUSA300_0038 | ccrA | cassette chromosome recombinase A | 0.000000 | 4.4 |
| SAUSA300_0039 | . | conserved hypothetical protein | 0.000000 | 3.1 |
| SAUSA300_0040 | . | conserved hypothetical protein | 0.010891 | 2.5 |
| SAUSA300_0043 | . | conserved hypothetical protein | 0.000916 | 4.5 |
| SAUSA300_0044 | . | metallo-beta-lactamase family protein | 0.001065 | 2.5 |
| SAUSA300_0045 | . | HNH endonuclease family protein | 0.000000 | 4.4 |
| SAUSA300_0047 | . | conserved hypothetical protein | 0.000000 | 2.7 |
| SAUSA300_0048 | . | hypothetical protein | 0.000136 | 2.7 |
| SAUSA300_0049 | . | hypothetical protein | 0.000008 | 2.3 |
| SAUSA300_0051 | . | . | 0.000971 | 2.8 |
| SAUSA300_0052 | . | hypothetical protein | 0.032315 | 3.2 |
| SAUSA300_0056 | . | conserved hypothetical protein | 0.084350 | 3.1 |
| SAUSA300_0057 | . | conserved hypothetical protein | 0.000196 | 4.0 |
| SAUSA300_0058 | . | conserved hypothetical protein | 0.054932 | 4.9 |
| SAUSA300_0059 | . | conserved hypothetical protein | 0.011885 | 4.4 |
| SAUSA300_0060 | . | putative transposase | 0.000000 | 3.4 |
| SAUSA300_0072 | . | hypothetical protein | 0.000000 | 2.2 |
| SAUSA300_0073 | . | peptide ABC transporter, peptide-binding protein | 0.000000 | 2.2 |
| SAUSA300_0074 | opp-3B | oligopeptide permease, channel-forming protein | 0.000000 | 2.8 |
| SAUSA300_0075 | opp-3C | oligopeptide permease, channel-forming protein | 0.000000 | 2.9 |
| SAUSA300_0076 | . | ABC transporter, ATP-binding protein | 0.000000 | 5.1 |
| SAUSA300_0077 | . | ABC transporter, ATP-binding protein | 0.000000 | 6.4 |
| SAUSA300_0082 | . | conserved hypothetical protein | 0.000000 | 3.4 |
| SAUSA300_0083 | . | putative membrane protein | 0.000000 | 3.8 |
| SAUSA300_0084 | . | conserved hypothetical protein | 0.000000 | 5.4 |
| SAUSA300_0085 | . | conserved hypothetical protein | 0.000000 | 6.4 |
| SAUSA300_0086 | . | conserved hypothetical protein | 0.000000 | 3.8 |
| SAUSA300_0088 | . | hypothetical protein | 0.000000 | 3.0 |
| SAUSA300_0091 | . | putative permease | 0.000000 | 2.9 |
| SAUSA300_0092 | . | conserved hypothetical protein | 0.000000 | 5.5 |
| SAUSA300_0093 | . | transcriptional regulator, LysR family domain protein | 0.000000 | 3.2 |
| SAUSA300_0095 | . | transcriptional regulator, LysR family domain protein | 0.000000 | 3.1 |
| SAUSA300_0096 | . | conserved hypothetical protein | 0.000000 | 3.3 |
| SAUSA300_0109 | . | integral membrane domain protein | 0.000022 | 4.2 |
| SAUSA300_0110 | . | transcriptional regulator, GntR family/aminotransferase, class I | 0.000000 | 3.0 |
| SAUSA300_0113 | . | immunoglobulin G binding protein A precursor | 0.000003 | 5.0 |
| SAUSA300_0114 | . | staphylococcal accessory regulator | 0.000076 | 2.0 |
| SAUSA300_0116 | sirB | iron compound ABC transporter, permease protein SirB | 0.000000 | 3.3 |
| SAUSA300_0132 | . | glycosyl transferase, group 1 family protein | 0.000070 | 3.2 |
| SAUSA300_0133 | . | putative membrane protein | 0.000066 | 3.3 |
| SAUSA300_0142 | phnE | phosphonate ABC transporter, permease protein | 0.000000 | 2.7 |
| SAUSA300_0143 | phnE | phosphonate ABC transporter, permease protein | 0.000076 | 2.3 |
| SAUSA300_0144 | phnC | phosphonate ABC transporter, ATP-binding protein | 0.002043 | 2.1 |
| SAUSA300_0146 | . | conserved hypothetical protein | 0.000000 | 2.4 |
| SAUSA300_0148 | . | conserved hypothetical protein | 0.069412 | 4.5 |
| SAUSA300_0173 | . | conserved hypothetical protein | 0.000000 | 17.8 |
| SAUSA300_0174 | . | conserved hypothetical protein | 0.000000 | 21.4 |
| SAUSA300_0175 | . | putative lipoprotein | 0.000000 | 24.9 |
| SAUSA300_0176 | . | ABC transporter, permease protein | 0.000000 | 28.2 |
| SAUSA300_0177 | . | conserved hypothetical protein | 0.000000 | 15.1 |
| SAUSA300_0178 | . | conserved hypothetical protein | 0.000000 | 2.1 |
| SAUSA300_0179 | . | putative D-isomer specific 2-hydroxyacid dehydrogenase | 0.000000 | 6.7 |
| SAUSA300_0184 | argB | acetylglutamate kinase | 0.000000 | 3.2 |
| SAUSA300_0185 | argJ | arginine biosynthesis bifunctional protein ArgJ | 0.000000 | 3.2 |
| SAUSA300_0186 | argC | N-acetyl-gamma-glutamyl-phosphate reductase | 0.000000 | 2.6 |
| SAUSA300_0187 | rocD | ornithine--oxo-acid transaminase | 0.000000 | 4.6 |
| SAUSA300_0191 | ptsG | PTS system, glucose-specific IIBC component domain protein | 0.000000 | 2.3 |
| SAUSA300_0200 | . | peptide ABC transporter, ATP-binding protein | 0.000000 | 4.1 |
| SAUSA300_0201 | . | peptide ABC transporter, permease protein | 0.000345 | 2.2 |
| SAUSA300_0207 | . | conserved hypothetical protein | 0.000000 | 2.5 |
| SAUSA300_0215 | . | conserved hypothetical protein | 0.000000 | 6.1 |
| SAUSA300_0216 | uhpT | hexose phosphate transport protein | 0.025470 | 2.2 |
| SAUSA300_0222 | . | putative membrane protein | 0.000000 | 2.2 |
| SAUSA300_0223 | . | conserved hypothetical protein | 0.000636 | 2.2 |
| SAUSA300_0224 | coa | staphylocoagulase precursor | 0.000000 | 22.6 |
| SAUSA300_0230 | . | putative membrane protein | 0.000000 | 2.8 |
| SAUSA300_0237 | . | inosine-uridine preferring nucleoside hydrolase | 0.000000 | 3.1 |
| SAUSA300_0240 | . | PTS system, galactitol-specific enzyme II, B component | 0.087529 | 5.4 |
| SAUSA300_0248 | . | putative teichoic acid biosynthesis protein F | 0.000000 | 2.0 |
| SAUSA300_0254 | . | sensor histidine kinase | 0.000000 | 3.5 |
| SAUSA300_0255 | . | sensory transduction protein LytR | 0.000000 | 6.7 |
| SAUSA300_0261 | . | conserved hypothetical protein | 0.000000 | 2.4 |
| SAUSA300_0267 | . | transposase | 0.000000 | 8.0 |
| SAUSA300_0270 | lytM | peptidoglycan hydrolase | 0.000000 | 2.9 |
| SAUSA300_0275 | . | putative membrane protein | 0.007261 | 2.2 |
| SAUSA300_0276 | . | putative membrane protein | 0.000000 | 2.7 |
| SAUSA300_0302 | . | conserved hypothetical protein | 0.000000 | 2.9 |
| SAUSA300_0307 | . | 5-nucleotidase, lipoprotein e(P4) family | 0.000000 | 4.1 |
| SAUSA300_0323 | . | conserved hypothetical protein | 0.000000 | 7.6 |
| SAUSA300_0336 | . | conserved hypothetical protein | 0.000006 | 2.0 |
| SAUSA300_0347 | tatC | Sec-independent protein translocase TatC | 0.000000 | 2.8 |
| SAUSA300_0348 | . | twin-arginine translocation protein, TatA/E family | 0.000000 | 2.3 |
| SAUSA300_0349 | . | conserved hypothetical protein | 0.000000 | 2.3 |
| SAUSA300_0354 | ltrA | low temperature requirement protein LtrA | 0.000000 | 2.9 |
| SAUSA300_0359 | . | trans-sulfuration enzyme family protein | 0.000000 | 2.6 |
| SAUSA300_0360 | . | Cys/Met metabolism PLP-dependent enzyme | 0.000000 | 4.3 |
| SAUSA300_0365 | . | conserved hypothetical protein | 0.000000 | 147.2 |
| SAUSA300_0366 | rpsF | ribosomal protein S6 | 0.000000 | 2.2 |
| SAUSA300_0367 | ssb | single-strand binding protein | 0.000000 | 2.1 |
| SAUSA300_0368 | rpsR | ribosomal protein S18 | 0.000000 | 2.1 |
| SAUSA300_0371 | . | conserved hypothetical protein | 0.000002 | 5.1 |
| SAUSA300_0382 | . | sodium:dicarboxylate symporter family protein | 0.000000 | 3.1 |
| SAUSA300_0386 | xpt | xanthine phosphoribosyltransferase | 0.000000 | 5.6 |
| SAUSA300_0387 | pbuX | xanthine permease | 0.000000 | 4.2 |
| SAUSA300_0388 | guaB | inosine-5-monophosphate dehydrogenase | 0.000000 | 3.4 |
| SAUSA300_0389 | guaA | GMP synthase | 0.000000 | 3.3 |
| SAUSA300_0397 | . | exotoxin | 0.000004 | 3.3 |
| SAUSA300_0398 | . | exotoxin | 0.005076 | 2.6 |
| SAUSA300_0403 | . | exotoxin | 0.000459 | 2.1 |
| SAUSA300_0405 | hsdM | type I restriction-modification system, M subunit | 0.000000 | 2.1 |
| SAUSA300_0406 | . | putative restriction/modification system specificity protein | 0.000000 | 2.6 |
| SAUSA300_0407 | . | exotoxin | 0.000000 | 7.4 |
| SAUSA300_0408 | . | putative surface protein | 0.000000 | 4.6 |
| SAUSA300_0423 | . | conserved hypothetical protein | 0.000000 | 6.1 |
| SAUSA300_0424 | . | putative cobalamin synthesis protein | 0.000000 | 2.3 |
| SAUSA300_0428 | . | conserved hypothetical protein | 0.010435 | 2.3 |
| SAUSA300_0431 | . | conserved hypothetical protein | 0.001756 | 5.4 |
| SAUSA300_0432 | . | sodium dependent transporter | 0.000000 | 27.2 |
| SAUSA300_0433 | cysM | cysteine synthase/cystathionine beta-synthase | 0.000000 | 12.8 |
| SAUSA300_0434 | metB | cystathionine gamma-synthase | 0.000000 | 8.5 |
| SAUSA300_0435 | . | ABC transporter, ATP-binding protein | 0.000001 | 2.5 |
| SAUSA300_0436 | . | ABC transporter, permease protein | 0.000000 | 3.2 |
| SAUSA300_0443 | . | YibE/F-like protein | 0.000000 | 3.3 |
| SAUSA300_0445 | gltB | glutamate synthase, large subunit | 0.000000 | 2.7 |
| SAUSA300_0446 | gltD | glutamate synthase, small subunit | 0.000000 | 2.9 |
| SAUSA300_0447 | . | tRNA-Ser | 0.000000 | 5.3 |
| SAUSA300_0448 | treP | PTS system, trehalose-specific IIBC component | 0.000000 | 2.6 |
| SAUSA300_0457 | rrfA | 5S ribosomal RNA | 0.116888 | 2.5 |
| SAUSA300_0461 | holB | DNA polymerase III delta subunit | 0.000000 | 2.2 |
| SAUSA300_0464 | . | Methyltransferase | 0.000000 | 2.1 |
| SAUSA300_0465 | . | conserved hypothetical protein | 0.000003 | 2.6 |
| SAUSA300_0466 | . | conserved hypothetical protein | 0.000000 | 2.4 |
| SAUSA300_0479 | . | ribosomal protein L25, Ctc-form | 0.000002 | 2.5 |
| SAUSA300_0486 | . | polyribonucleotide nucleotidyltransferase | 0.000000 | 2.1 |
| SAUSA300_0487 | . | MesJ/Ycf62 family protein | 0.000000 | 2.5 |
| SAUSA300_0490 | . | 33 kDa chaperonin (Heat shock protein 33-like protein) | 0.000000 | 2.2 |
| SAUSA300_0494 | folK | 2-amino-4-hydroxy-6- hydroxymethyldihydropteridine pyrophosphokinase | 0.000000 | 2.7 |
| SAUSA300_0497 | rrfG | 5S ribosomal RNA | 0.000035 | 5.2 |
| SAUSA300_0502 | rrfB | 5S ribosomal RNA | 0.116888 | 2.5 |
| SAUSA300_0506 | nupC | pyrimidine nucleoside transport protein | 0.000000 | 2.2 |
| SAUSA300_0522 | rplK | ribosomal protein L11 | 0.000000 | 2.8 |
| SAUSA300_0523 | rplA | ribosomal protein L1 | 0.000000 | 3.5 |
| SAUSA300_0524 | rplJ | ribosomal protein L10 | 0.000000 | 3.1 |
| SAUSA300_0525 | rplL | ribosomal protein L7/L12 | 0.000003 | 2.7 |
| SAUSA300_0526 | . | Methyltransferase small domain | 0.000000 | 4.2 |
| SAUSA300_0542 | . | deoxynucleoside kinase family protein | 0.000000 | 3.3 |
| SAUSA300_0548 | sdrE | sdrE protein | 0.000000 | 2.8 |
| SAUSA300_0553 | . | conserved hypothetical protein | 0.000000 | 2.1 |
| SAUSA300_0559 | . | putative substrate--CoA ligase | 0.000002 | 2.2 |
| SAUSA300_0561 | . | hypothetical protein | 0.003622 | 2.4 |
| SAUSA300_0563 | ung | uracil-DNA glycosylase | 0.000000 | 2.5 |
| SAUSA300_0564 | . | conserved hypothetical protein | 0.000000 | 2.8 |
| SAUSA300_0576 | . | putative Pyridine nucleotide-disulphide oxidoreductase | 0.000000 | 5.9 |
| SAUSA300_0577 | . | putative transcriptional regulator | 0.000000 | 19.1 |
| SAUSA300_0578 | . | conserved hypothetical protein | 0.000672 | 8.1 |
| SAUSA300_0586 | . | conserved hypothetical protein | 0.000168 | 2.0 |
| SAUSA300_0607 | . | conserved hypothetical protein | 0.000357 | 2.8 |
| SAUSA300_0608 | . | conserved hypothetical protein | 0.010219 | 2.0 |
| SAUSA300_0629 | pbp4 | penicillin-binding protein 4 | 0.000000 | 2.4 |
| SAUSA300_0640 | . | putative membrane protein | 0.000000 | 3.3 |
| SAUSA300_0641 | . | putative lipase/esterase | 0.000000 | 3.3 |
| SAUSA300_0650 | . | phosphate transporter family protein | 0.000000 | 2.3 |
| SAUSA300_0653 | . | transcriptional regulator, AraC family | 0.000000 | 2.5 |
| SAUSA300_0654 | sarX | staphylococcal accessory protein X | 0.000000 | 2.9 |
| SAUSA300_0681 | . | conserved hypothetical protein | 0.000000 | 2.1 |
| SAUSA300_0683 | . | transcriptional regulator, DeoR family | 0.000000 | 22.9 |
| SAUSA300_0684 | fruB | fructose 1-phosphate kinase | 0.000000 | 11.9 |
| SAUSA300_0685 | fruA | fructose specific permease | 0.000000 | 6.7 |
| SAUSA300_0690 | saeS | sensor histidine kinase SaeS | 0.000005 | 2.2 |
| SAUSA300_0694 | . | putative membrane protein | 0.000034 | 2.6 |
| SAUSA300_0695 | . | radical activating enzyme family protein | 0.000000 | 2.5 |
| SAUSA300_0696 | . | 6-pyruvoyl tetrahydrobiopterin synthase-like protein | 0.000000 | 2.5 |
| SAUSA300_0697 | . | exsB protein | 0.000000 | 11.7 |
| SAUSA300_0713 | folE | GTP cyclohydrolase I | 0.000000 | 3.2 |
| SAUSA300_0714 | . | Integral membrane protein | 0.000000 | 2.8 |
| SAUSA300_0715 | nrdI | nrdI protein | 0.000000 | 2.5 |
| SAUSA300_0716 | . | ribonucleoside-diphosphate reductase, alpha subunit | 0.000000 | 2.6 |
| SAUSA300_0717 | . | ribonucleoside-diphosphate reductase, beta subunit | 0.000000 | 4.4 |
| SAUSA300_0718 | . | iron compound ABC transporter, permease | 0.000000 | 2.6 |
| SAUSA300_0719 | . | iron compound ABC transporter, permease protein | 0.000000 | 3.4 |
| SAUSA300_0720 | . | putative iron compound ABC transporter, ATP-binding protein | 0.000000 | 3.9 |
| SAUSA300_0721 | . | transferrin receptor | 0.000000 | 3.2 |
| SAUSA300_0730 | . | GGDEF domain protein | 0.000000 | 2.0 |
| SAUSA300_0734 | . | putative comf operon protein 1 | 0.000000 | 4.4 |
| SAUSA300_0739 | . | LysM domain protein | 0.000000 | 4.5 |
| SAUSA300_0741 | uvrB | excinuclease ABC, B subunit | 0.000000 | 3.0 |
| SAUSA300_0742 | uvrA | excinuclease ABC, A subunit | 0.000000 | 2.4 |
| SAUSA300_0755 | . | glycolytic operon regulator | 0.000000 | 3.2 |
| SAUSA300_0773 | . | putative staphylocoagulase | 0.000000 | 3.7 |
| SAUSA300_0774 | empbp | secretory extracellular matrix and plasma binding protein | 0.000027 | 2.8 |
| SAUSA300_0775 | . | conserved hypothetical protein | 0.075320 | 2.4 |
| SAUSA300_0776 | nuc | thermonuclease precursor | 0.000000 | 2.3 |
| SAUSA300_0782 | . | conserved hypothetical protein | 0.000021 | 2.6 |
| SAUSA300_0783 | . | phosphoglycerate mutase family protein | 0.000000 | 2.7 |
| SAUSA300_0799 | int | integrase | 0.000000 | 18.4 |
| SAUSA300_0800 | sek | staphylococcal enterotoxin K | 0.000000 | 4.0 |
| SAUSA300_0801 | seq | staphylococcal enterotoxin Q | 0.000000 | 4.3 |
| SAUSA300_0802 | . | conserved hypothetical protein | 0.000000 | 4.5 |
| SAUSA300_0803 | . | transcriptional regulator, Cro/CI family | 0.000000 | 5.7 |
| SAUSA300_0804 | . | putative transcriptional regulator | 0.000000 | 27.3 |
| SAUSA300_0805 | . | pathogenicity island protein | 0.000000 | 20.9 |
| SAUSA300_0806 | . | conserved hypothetical protein | 0.000000 | 22.0 |
| SAUSA300_0807 | . | conserved hypothetical protein | 0.000000 | 22.8 |
| SAUSA300_0808 | . | conserved hypothetical protein | 0.000000 | 20.8 |
| SAUSA300_0809 | . | putative DNA primase | 0.000000 | 19.6 |
| SAUSA300_0810 | . | conserved hypothetical protein | 0.000000 | 12.8 |
| SAUSA300_0811 | . | conserved hypothetical protein | 0.000000 | 16.3 |
| SAUSA300_0812 | . | conserved hypothetical protein | 0.000000 | 13.7 |
| SAUSA300_0813 | . | conserved hypothetical protein | 0.000000 | 12.8 |
| SAUSA300_0846 | . | Na+/H+ antiporter family protein | 0.000000 | 13.2 |
| SAUSA300_0847 | . | conserved hypothetical protein | 0.000000 | 2.1 |
| SAUSA300_0863 | argH | argininosuccinate lyase | 0.000000 | 5.1 |
| SAUSA300_0864 | argG | argininosuccinate synthase | 0.000000 | 5.4 |
| SAUSA300_0900 | . | putative competence protein | 0.000000 | 5.2 |
| SAUSA300_0901 | . | putative competence protein | 0.000005 | 3.0 |
| SAUSA300_0922 | . | membrane protein, TerC family | 0.000000 | 2.8 |
| SAUSA300_0926 | . | tRNA-Ser | 0.114198 | 3.5 |
| SAUSA300_0986 | . | cytochrome D ubiquinol oxidase, subunit I | 0.000000 | 3.3 |
| SAUSA300_0987 | . | cytochrome D ubiquinol oxidase, subunit II | 0.000000 | 6.2 |
| SAUSA300_0991 | def | peptide deformylase | 0.000000 | 2.1 |
| SAUSA300_1009 | typA | GTP-binding protein | 0.000000 | 2.9 |
| SAUSA300_1026 | . | conserved hypothetical protein | 0.000000 | 3.7 |
| SAUSA300_1027 | rpmF | 50S ribosomal protein L32 | 0.000000 | 2.5 |
| SAUSA300_1028 | . | iron transport associated domain protein | 0.000003 | 2.1 |
| SAUSA300_1065 | . | exfoliative toxin A | 0.000000 | 2.1 |
| SAUSA300_1066 | . | tRNA-Arg | 0.004084 | 2.6 |
| SAUSA300_1091 | pyrR | PyrR bifunctional protein | 0.000000 | 3.7 |
| SAUSA300_1092 | pyrP | uracil permease | 0.000000 | 12.8 |
| SAUSA300_1093 | pyrB | aspartate carbamoyltransferase | 0.000000 | 9.5 |
| SAUSA300_1094 | pyrC | dihydroorotase | 0.000000 | 12.8 |
| SAUSA300_1095 | carA | carbamoyl-phosphate synthase, small subunit | 0.000000 | 8.6 |
| SAUSA300_1096 | carB | carbamoyl-phosphate synthase, large subunit | 0.000000 | 6.8 |
| SAUSA300_1097 | pyrF | orotidine 5-phosphate decarboxylase | 0.000000 | 6.0 |
| SAUSA300_1098 | pyrE | orotate phosphoribosyltransferase | 0.000000 | 5.7 |
| SAUSA300_1113 | pknB | protein kinase | 0.000000 | 2.1 |
| SAUSA300_1131 | rpsP | 30S ribosomal protein S16 | 0.000000 | 2.1 |
| SAUSA300_1132 | rimM | 16S rRNA processing protein RimM | 0.000000 | 3.1 |
| SAUSA300_1133 | trmD | tRNA (guanine-N1)-methyltransferase | 0.000000 | 4.1 |
| SAUSA300_1151 | pyrH | uridylate kinase | 0.000000 | 2.7 |
| SAUSA300_1153 | uppS | undecaprenyl diphosphate synthase | 0.000000 | 2.6 |
| SAUSA300_1154 | cdsA | phosphatidate cytidylyltransferase | 0.000000 | 3.5 |
| SAUSA300_1178 | recA | recombinase A protein | 0.000000 | 4.5 |
| SAUSA300_1238 | . | conserved hypothetical protein | 0.000000 | 5.9 |
| SAUSA300_1242 | sbcD | exonuclease SbcD | 0.000000 | 6.1 |
| SAUSA300_1243 | sbcC | exonuclease SbcC | 0.000000 | 4.9 |
| SAUSA300_1245 | opuD | glycine betaine transporter opuD | 0.000000 | 2.7 |
| SAUSA300_1250 | parE | DNA topoisomerase IV, subunit B | 0.000000 | 2.8 |
| SAUSA300_1251 | parC | DNA topoisomerase IV, subunit A | 0.000000 | 2.9 |
| SAUSA300_1252 | . | amino acid carrier protein | 0.000000 | 2.8 |
| SAUSA300_1259 | . | ImpB/MucB/SamB family protein | 0.000000 | 231.9 |
| SAUSA300_1261 | . | putative glutamyl aminopeptidase | 0.000000 | 13.4 |
| SAUSA300_1262 | trpE | anthranilate synthase component I | 0.000002 | 3.1 |
| SAUSA300_1300 | brnQ | branched-chain amino acid transport system II carrier protein | 0.000000 | 5.4 |
| SAUSA300_1324 | . | putative membrane protein | 0.000000 | 2.8 |
| SAUSA300_1328 | . | putative drug transporter | 0.000000 | 2.6 |
| SAUSA300_1331 | ald | alanine dehydrogenase | 0.000000 | 2.2 |
| SAUSA300_1334 | . | putative membrane protein | 0.000000 | 5.6 |
| SAUSA300_1336 | . | conserved hypothetical protein | 0.000000 | 2.0 |
| SAUSA300_1352 | . | putative membrane protein | 0.000000 | 2.4 |
| SAUSA300_1377 | . | conserved hypothetical protein | 0.019473 | 2.2 |
| SAUSA300_1378 | . | conserved hypothetical protein | 0.000000 | 4.2 |
| SAUSA300_1379 | . | putative lipoprotein | 0.000000 | 12.8 |
| SAUSA300_1380 | . | conserved hypothetical protein | 0.000000 | 7.5 |
| SAUSA300_1424 | . | conserved hypothetical phage protein | 0.006791 | 2.5 |
| SAUSA300_1425 | . | conserved hypothetical phage protein | 0.002401 | 3.0 |
| SAUSA300_1426 | . | conserved hypothetical phage protein | 0.279288 | 2.2 |
| SAUSA300_1427 | . | phiSLT ORF86-like protein | 0.121835 | 3.6 |
| SAUSA300_1428 | . | conserved hypothetical phage protein | 0.000999 | 21.5 |
| SAUSA300_1429 | . | phiSLT ORF53-like protein | 0.019657 | 5.5 |
| SAUSA300_1430 | . | phiSLT ORF 87-like protein, putative DNA-binding protein | 0.000919 | 8.1 |
| SAUSA300_1431 | . | phiSLT ORF71-like protein | 0.000027 | 25.4 |
| SAUSA300_1433 | . | putative phage regulatory protein | 0.211592 | 2.6 |
| SAUSA300_1438 | . | phiSLT ORF401-like protein, integrase | 0.000000 | 2.9 |
| SAUSA300_1517 | . | endonuclease IV | 0.000000 | 2.2 |
| SAUSA300_1543 | . | oxygen-independent coproporphyrinogen III oxidase | 0.000000 | 2.9 |
| SAUSA300_1546 | holA | DNA polymerase III, delta subunit | 0.000000 | 2.4 |
| SAUSA300_1568 | udk | uridine kinase | 0.000000 | 2.0 |
| SAUSA300_1569 | . | peptidase, U32 family | 0.000000 | 2.8 |
| SAUSA300_1570 | . | peptidase, U32 family | 0.000000 | 2.5 |
| SAUSA300_1571 | . | O-methyltransferase family protein | 0.000000 | 3.1 |
| SAUSA300_1576 | . | helicase, RecD/TraA family | 0.000000 | 2.2 |
| SAUSA300_1577 | . | TPR domain protein | 0.000000 | 3.4 |
| SAUSA300_1591 | apt | adenine phosphoribosyltransferase | 0.000000 | 2.3 |
| SAUSA300_1592 | recJ | single-stranded-DNA-specific exonuclease RecJ | 0.000000 | 2.4 |
| SAUSA300_1595 | tgt | queuine tRNA-ribosyltransferase | 0.000000 | 2.6 |
| SAUSA300_1596 | queA | S-adenosylmethionine:tRNA ribosyltransferase-isomerase | 0.000000 | 3.3 |
| SAUSA300_1597 | ruvB | holliday junction DNA helicase RuvB | 0.000000 | 2.6 |
| SAUSA300_1598 | ruvA | holliday junction DNA helicase RuvA | 0.000000 | 2.1 |
| SAUSA300_1623 | . | conserved hypothetical protein | 0.000000 | 2.4 |
| SAUSA300_1624 | . | MutT/nudix family protein | 0.000000 | 2.9 |
| SAUSA300_1625 | rplT | 50S ribosomal protein L20 | 0.000000 | 2.6 |
| SAUSA300_1626 | rpmI | 50S ribosomal protein L35 | 0.000000 | 2.5 |
| SAUSA300_1627 | infC | translation initiation factor IF-3 | 0.000000 | 2.8 |
| SAUSA300_1643 | . | . | 0.000000 | 2.7 |
| SAUSA300_1646 | accA | acetyl-CoA carboxylase, carboxyl transferase, alpha subunit | 0.000000 | 2.0 |
| SAUSA300_1660 | . | putative membrane protein | 0.000000 | 3.3 |
| SAUSA300_1661 | thiI | thiamine biosynthesis protein ThiI | 0.000000 | 3.1 |
| SAUSA300_1662 | . | aminotransferase, class V | 0.000000 | 2.6 |
| SAUSA300_1694 | trmB | tRNA (guanine-N(7)-)-methyltransferase | 0.000000 | 2.3 |
| SAUSA300_1705 | . | putative drug transporter | 0.000000 | 2.1 |
| SAUSA300_1706 | . | conserved hypothetical protein | 0.000000 | 2.6 |
| SAUSA300_1707 | . | conserved hypothetical protein | 0.000000 | 3.4 |
| SAUSA300_1709 | . | . | 0.000000 | 7.8 |
| SAUSA300_1724 | . | abortive infection protein family | 0.000000 | 3.3 |
| SAUSA300_1726 | . | crcB family protein | 0.000000 | 2.4 |
| SAUSA300_1730 | metK | S-adenosylmethionine synthetase | 0.000000 | 2.5 |
| SAUSA300_1738 | . | putative lipoprotein | 0.000000 | 3.1 |
| SAUSA300_1772 | . | tRNA-Ser | 0.000000 | 3.2 |
| SAUSA300_1778 | . | tRNA-Asp | 0.000000 | 6.2 |
| SAUSA300_1779 | . | tRNA-Met | 0.000000 | 7.3 |
| SAUSA300_1780 | . | conserved hypothetical protein | 0.000000 | 2.0 |
| SAUSA300_1794 | . | conserved hypothetical protein | 0.000000 | 15.7 |
| SAUSA300_1797 | . | conserved hypothetical protein | 0.000000 | 3.1 |
| SAUSA300_1800 | . | ribosomal large subunit pseudouridine synthase, RluD subfamily | 0.000000 | 2.4 |
| SAUSA300_1809 | . | putative membrane protein | 0.000000 | 2.1 |
| SAUSA300_1811 | . | tRNA-Leu | 0.000000 | 6.1 |
| SAUSA300_1812 | . | tRNA-OTHER | 0.000000 | 4.3 |
| SAUSA300_1813 | . | tRNA-Gly | 0.000000 | 6.5 |
| SAUSA300_1814 | . | tRNA-Cys | 0.000000 | 14.2 |
| SAUSA300_1815 | . | tRNA-Gln | 0.000000 | 9.4 |
| SAUSA300_1816 | . | tRNA-His | 0.000000 | 10.8 |
| SAUSA300_1817 | . | tRNA-Trp | 0.000000 | 13.8 |
| SAUSA300_1818 | . | tRNA-Tyr | 0.000000 | 8.7 |
| SAUSA300_1819 | . | tRNA-Thr | 0.000000 | 5.9 |
| SAUSA300_1820 | . | tRNA-Phe | 0.000000 | 4.6 |
| SAUSA300_1821 | . | tRNA-Asp | 0.000000 | 7.2 |
| SAUSA300_1822 | . | tRNA-Met | 0.000000 | 7.8 |
| SAUSA300_1823 | . | tRNA-Ser | 0.000000 | 5.7 |
| SAUSA300_1824 | . | tRNA-Asp | 0.000000 | 6.4 |
| SAUSA300_1825 | . | tRNA-Ser | 0.000000 | 6.2 |
| SAUSA300_1826 | . | tRNA-Met | 0.000000 | 6.0 |
| SAUSA300_1827 | . | tRNA-Met | 0.000000 | 9.8 |
| SAUSA300_1828 | . | tRNA-Ala | 0.000009 | 2.8 |
| SAUSA300_1829 | . | tRNA-Pro | 0.000000 | 2.2 |
| SAUSA300_1830 | . | tRNA-Arg | 0.000000 | 3.9 |
| SAUSA300_1831 | . | tRNA-Leu | 0.000000 | 2.4 |
| SAUSA300_1832 | . | tRNA-Gly | 0.000919 | 2.4 |
| SAUSA300_1833 | . | tRNA-Leu | 0.000004 | 3.4 |
| SAUSA300_1834 | . | tRNA-Lys | 0.000035 | 4.1 |
| SAUSA300_1835 | . | tRNA-Thr | 0.000000 | 5.1 |
| SAUSA300_1836 | . | tRNA-Val | 0.036702 | 6.0 |
| SAUSA300_1840 | . | tRNA-Ile | 0.007161 | 9.0 |
| SAUSA300_1847 | . | conserved hypothetical protein | 0.000000 | 3.1 |
| SAUSA300_1858 | . | conserved hypothetical protein | 0.000000 | 2.1 |
| SAUSA300_1870 | . | conserved hypothetical protein | 0.000000 | 2.4 |
| SAUSA300_1872 | . | conserved hypothetical protein | 0.000000 | 2.7 |
| SAUSA300_1881 | gatA | Aspartyl/glutamyl-tRNA amidotransferase subunit A | 0.000000 | 2.3 |
| SAUSA300_1882 | gatC | aspartyl/glutamyl-tRNA amidotransferase subunit C | 0.000000 | 2.7 |
| SAUSA300_1903 | . | conserved hypothetical protein | 0.000000 | 161.0 |
| SAUSA300_1920 | chs | chemotaxis-inhibiting protein CHIPS | 0.005877 | 3.9 |
| SAUSA300_1921 | . | truncated amidase | 0.000000 | 2.5 |
| SAUSA300_1927 | . | phi77 ORF109-like protein | 0.086866 | 2.3 |
| SAUSA300_1943 | . | phi77 ORF040-like protein | 0.000008 | 2.4 |
| SAUSA300_1944 | . | phi77 ORF026-like protein, putative phage transcriptional activator | 0.000000 | 2.6 |
| SAUSA300_1945 | . | phi77 ORF071-like protein | 0.000000 | 2.7 |
| SAUSA300_1946 | . | phiPVL ORF057-like protein, transcriptional activator RinB | 0.000007 | 2.8 |
| SAUSA300_1947 | . | phi77 ORF031-like protein | 0.016279 | 2.2 |
| SAUSA300_1948 | . | phi77 ORF069-like protein | 0.000021 | 2.7 |
| SAUSA300_1949 | dut | dUTP diphosphatase | 0.000000 | 3.9 |
| SAUSA300_1951 | . | phiPVL ORF052-like protein | 0.000005 | 3.9 |
| SAUSA300_1952 | . | phiPV083 ORF027-like protein | 0.000507 | 5.9 |
| SAUSA300_1953 | . | phiPVL ORF051-like protein | 0.000000 | 3.8 |
| SAUSA300_1954 | . | phiPVL ORF050-like protein | 0.000000 | 4.8 |
| SAUSA300_1955 | . | putative endodeoxyribonuclease RusA | 0.000000 | 5.2 |
| SAUSA300_1956 | . | conserved hypothetical phage protein | 0.000000 | 6.2 |
| SAUSA300_1957 | . | phiPVL ORF046-like protein | 0.000000 | 4.6 |
| SAUSA300_1958 | . | Single-strand binding protein | 0.000000 | 5.5 |
| SAUSA300_1959 | . | phiPVL ORF044-like protein | 0.000000 | 3.1 |
| SAUSA300_1960 | . | putative phage-related DNA recombination protein | 0.000000 | 3.9 |
| SAUSA300_1961 | . | phiPVL ORF41-like protein | 0.000000 | 3.6 |
| SAUSA300_1962 | . | phiPVL ORF39-like protein | 0.000000 | 4.1 |
| SAUSA300_1963 | . | conserved hypothetical phage protein | 0.000000 | 6.1 |
| SAUSA300_1964 | . | conserved hypothetical phage protein | 0.000000 | 6.4 |
| SAUSA300_1966 | . | phi77 ORF014-like protein, phage anti-repressor protein | 0.000000 | 4.8 |
| SAUSA300_1967 | . | conserved hypothetical phage protein | 0.000000 | 3.5 |
| SAUSA300_1968 | . | putative phage transcriptional regulator | 0.000000 | 6.1 |
| SAUSA300_1969 | . | phi77 ORF011-like protein, phage transcriptional repressor | 0.000000 | 13.9 |
| SAUSA300_1970 | . | putative exonuclease | 0.000000 | 5.0 |
| SAUSA300_1971 | . | phi77 ORF017-like protein | 0.000040 | 2.2 |
| SAUSA300_1972 | int | integrase | 0.000000 | 4.0 |
| SAUSA300_1973 | . | truncated beta-hemolysin | 0.000000 | 8.0 |
| SAUSA300_1981 | . | phage terminase family protein | 0.000076 | 3.3 |
| SAUSA300_1985 | sdrH | serine-aspartate repeat family protein, SdrH | 0.000000 | 2.9 |
| SAUSA300_1996 | amt | ammonium transporter | 0.096680 | 2.2 |
| SAUSA300_1997 | . | conserved hypothetical protein | 0.000000 | 9.8 |
| SAUSA300_1998 | . | putative membrane protein | 0.000000 | 10.0 |
| SAUSA300_2019 | . | tRNA-Leu | 0.000000 | 15.4 |
| SAUSA300_2034 | kdpA | K+-transporting ATPase, A subunit | 0.000000 | 3.0 |
| SAUSA300_2037 | . | ATP-dependent RNA helicase | 0.000000 | 3.8 |
| SAUSA300_2046 | oxaA | membrane protein oxaA precursor | 0.000000 | 2.1 |
| SAUSA300_2051 | . | conserved hypothetical protein | 0.000000 | 8.1 |
| SAUSA300_2069 | . | conserved hypothetical protein | 0.000000 | 4.3 |
| SAUSA300_2070 | . | conserved hypothetical protein | 0.000000 | 4.1 |
| SAUSA300_2071 | . | modification methylase, HemK family | 0.000000 | 3.6 |
| SAUSA300_2072 | prfA | peptide chain release factor 1 | 0.000000 | 3.4 |
| SAUSA300_2073 | tdk | thymidine kinase | 0.000000 | 3.1 |
| SAUSA300_2074 | rpmE | 50S ribosomal protein L31 type B | 0.000000 | 2.9 |
| SAUSA300_2080 | . | conserved hypothetical protein | 0.000000 | 2.7 |
| SAUSA300_2081 | pyrG | CTP synthase | 0.000000 | 3.2 |
| SAUSA300_2085 | . | conserved hypothetical protein | 0.000000 | 2.2 |
| SAUSA300_2092 | dps | general stress protein 20U | 0.000000 | 2.3 |
| SAUSA300_2098 | arsR | transcriptional repressor, ArsR family | 0.000000 | 4.1 |
| SAUSA300_2099 | . | cation efflux family protein | 0.000000 | 4.2 |
| SAUSA300_2109 | fmtB | truncated FmtB protein | 0.000000 | 2.4 |
| SAUSA300_2110 | fmtB | truncated FmtB protein | 0.000000 | 2.6 |
| SAUSA300_2116 | . | tRNA-Lys | 0.000000 | 3.3 |
| SAUSA300_2117 | . | tRNA-Gln | 0.000000 | 5.0 |
| SAUSA300_2118 | . | tRNA-Tyr | 0.000076 | 3.3 |
| SAUSA300_2119 | . | tRNA-Val | 0.003188 | 2.5 |
| SAUSA300_2121 | . | tRNA-Asn | 0.024335 | 2.4 |
| SAUSA300_2128 | . | putative drug transporter | 0.000000 | 4.8 |
| SAUSA300_2129 | . | putative hemolysin III | 0.000000 | 3.5 |
| SAUSA300_2130 | . | UTP-glucose-1-phosphate uridylyltransferase family protein | 0.000000 | 2.6 |
| SAUSA300_2131 | . | conserved hypothetical protein | 0.000000 | 3.2 |
| SAUSA300_2133 | . | transporter gate domain protein | 0.000000 | 6.0 |
| SAUSA300_2134 | . | iron compound ABC transporter, permease protein | 0.000000 | 2.9 |
| SAUSA300_2135 | . | iron compound ABC transporter, permease protein | 0.000000 | 3.1 |
| SAUSA300_2139 | . | putative transporter | 0.000000 | 2.8 |
| SAUSA300_2140 | . | conserved hypothetical protein | 0.000000 | 2.2 |
| SAUSA300_2141 | . | . | 0.000000 | 7.9 |
| SAUSA300_2171 | rpsI | 30S ribosomal protein S9 | 0.000000 | 4.2 |
| SAUSA300_2172 | rplM | 50S ribosomal protein L13 | 0.000000 | 4.3 |
| SAUSA300_2173 | truA | tRNA pseudouridine synthase A | 0.000000 | 3.0 |
| SAUSA300_2174 | . | cobalt transport family protein | 0.000000 | 2.7 |
| SAUSA300_2175 | . | ABC transporter, ATP-binding protein | 0.000000 | 2.5 |
| SAUSA300_2177 | rplQ | 50S ribosomal protein L17 | 0.000000 | 2.2 |
| SAUSA300_2178 | rpoA | DNA-directed RNA polymerase alpha subunit | 0.000000 | 2.0 |
| SAUSA300_2179 | rpsK | 30S ribosomal protein S11 | 0.000000 | 2.4 |
| SAUSA300_2180 | rpsM | 30S ribosomal protein S13 | 0.000000 | 2.1 |
| SAUSA300_2181 | rpmJ | 50S ribosomal protein L36 | 0.000000 | 2.3 |
| SAUSA300_2182 | infA | translation initiation factor IF-1 | 0.000000 | 2.2 |
| SAUSA300_2183 | adk | adenylate kinase | 0.000000 | 2.3 |
| SAUSA300_2184 | . | preprotein translocase, SecY subunit | 0.000000 | 2.1 |
| SAUSA300_2185 | rplO | 50S ribosomal protein L15 | 0.000000 | 2.6 |
| SAUSA300_2186 | rpmD | 50S ribosomal protein L30 | 0.000000 | 3.5 |
| SAUSA300_2187 | rpsE | 30S ribosomal protein S5 | 0.000000 | 3.4 |
| SAUSA300_2188 | rplR | 50S ribosomal protein L18 | 0.000000 | 3.4 |
| SAUSA300_2189 | rplF | 50S ribosomal protein L6 | 0.000000 | 2.9 |
| SAUSA300_2190 | rpsH | 30S ribosomal protein S8 | 0.000000 | 2.9 |
| SAUSA300_2191 | rpsN | 30S ribosomal protein S14 | 0.000000 | 3.2 |
| SAUSA300_2192 | rplE | 50S ribosomal protein L5 | 0.000000 | 2.7 |
| SAUSA300_2193 | rplX | 50S ribosomal protein L24 | 0.000000 | 3.0 |
| SAUSA300_2194 | rplN | 50S ribosomal protein L14 | 0.000000 | 3.1 |
| SAUSA300_2195 | rpsQ | 30S ribosomal protein S17 | 0.000000 | 2.6 |
| SAUSA300_2196 | rpmC | 50S ribosomal protein L29 | 0.000000 | 2.5 |
| SAUSA300_2197 | rplP | 50S ribosomal protein L16 | 0.000000 | 2.6 |
| SAUSA300_2198 | rpsC | 30S ribosomal protein S3 | 0.000000 | 2.3 |
| SAUSA300_2199 | rplV | 50S ribosomal protein L22 | 0.000000 | 2.2 |
| SAUSA300_2200 | rpsS | 30S ribosomal protein S19 | 0.000000 | 2.5 |
| SAUSA300_2201 | rplB | 50S ribosomal protein L2 | 0.000000 | 3.2 |
| SAUSA300_2202 | rplW | 50S ribosomal protein L23 | 0.000000 | 2.5 |
| SAUSA300_2203 | rplD | 50S ribosomal protein L4 | 0.000000 | 2.4 |
| SAUSA300_2205 | rpsJ | 30S ribosomal protein S10 | 0.000000 | 2.4 |
| SAUSA300_2207 | . | xanthine/uracil permease family protein | 0.000000 | 2.2 |
| SAUSA300_2210 | glcU | probable glucose uptake protein | 0.000000 | 6.0 |
| SAUSA300_2212 | . | conserved hypothetical protein | 0.000000 | 2.2 |
| SAUSA300_2216 | . | transcriptional regulator, MarR family | 0.000195 | 2.4 |
| SAUSA300_2217 | . | putative drug transporter | 0.000005 | 2.4 |
| SAUSA300_2218 | . | staphylococcal accessory regulator | 0.000000 | 3.9 |
| SAUSA300_2231 | fdhD | formate dehydrogenase family accessory protein FdhD | 0.000000 | 2.1 |
| SAUSA300_2232 | . | acetyltransferase, GNAT family | 0.000000 | 5.3 |
| SAUSA300_2233 | . | BioY family protein | 0.000000 | 6.4 |
| SAUSA300_2237 | . | putative urea transporter | 0.000000 | 2.6 |
| SAUSA300_2249 | ssaA | secretory antigen precursor SsaA | 0.000000 | 35.4 |
| SAUSA300_2253 | ssaA | secretory antigen precursor SsaA | 0.000000 | 9.1 |
| SAUSA300_2255 | . | monooxygenase family protein | 0.000000 | 3.3 |
| SAUSA300_2256 | . | putative N-acetylmuramoyl-L-alanine amidase | 0.000000 | 3.3 |
| SAUSA300_2263 | . | putative transposase | 0.000267 | 2.1 |
| SAUSA300_2264 | . | phosphosugar-binding transcriptional regulator, RpiR family | 0.000000 | 2.2 |
| SAUSA300_2271 | . | phosphosugar-binding transcriptional regulator | 0.000000 | 3.8 |
| SAUSA300_2273 | . | Na+/H+ antiporter family protein | 0.000000 | 2.7 |
| SAUSA300_2282 | . | putative membrane protein | 0.000000 | 3.1 |
| SAUSA300_2285 | galM | aldose 1-epimerase | 0.000000 | 2.7 |
| SAUSA300_2286 | . | conserved hypothetical protein | 0.000000 | 2.5 |
| SAUSA300_2291 | gltS | sodium/glutamate symporter | 0.000000 | 5.1 |
| SAUSA300_2293 | corA | magnesium and cobalt transport protein | 0.000000 | 2.1 |
| SAUSA300_2301 | tcaB | teicoplanin resistance associated membrane protein TcaB protein | 0.000000 | 2.5 |
| SAUSA300_2305 | . | transposase, truncation | 0.001825 | 3.7 |
| SAUSA300_2306 | . | ABC transporter, ATP-binding protein | 0.000000 | 2.5 |
| SAUSA300_2307 | . | ABC transporter, permease protein | 0.000000 | 3.6 |
| SAUSA300_2321 | . | putative membrane protein | 0.000000 | 3.4 |
| SAUSA300_2324 | . | PTS system, sucrose-specific IIBC component | 0.000000 | 3.3 |
| SAUSA300_2329 | gltT | proton/sodium-glutamate symport protein | 0.000000 | 3.1 |
| SAUSA300_2330 | . | conserved hypothetical protein | 0.000000 | 2.5 |
| SAUSA300_2333 | narK | nitrite extrusion protein | 0.000000 | 2.5 |
| SAUSA300_2334 | . | conserved hypothetical protein | 0.000000 | 4.6 |
| SAUSA300_2335 | . | conserved hypothetical protein | 0.002235 | 3.5 |
| SAUSA300_2336 | . | transcriptional regulator, MerR family | 0.000000 | 2.4 |
| SAUSA300_2341 | narJ | respiratory nitrate reductase, delta subunit | 0.000000 | 2.5 |
| SAUSA300_2342 | narH | respiratory nitrate reductase, beta subunit | 0.000000 | 2.4 |
| SAUSA300_2343 | . | respiratory nitrate reductase, alpha subunit | 0.000000 | 2.8 |
| SAUSA300_2347 | nirR | nitrite reductase transcriptional regulator NirR | 0.000030 | 2.8 |
| SAUSA300_2349 | . | formate/nitrite transporter family protein | 0.000000 | 2.5 |
| SAUSA300_2357 | . | ABC transporter, ATP-binding protein | 0.000000 | 4.3 |
| SAUSA300_2358 | . | ABC transporter, permease protein | 0.000000 | 4.0 |
| SAUSA300_2359 | . | amino acid ABC transporter, amino acid-binding protein | 0.000000 | 4.7 |
| SAUSA300_2363 | . | cation efflux family protein | 0.000000 | 3.0 |
| SAUSA300_2364 | sbi | IgG-binding protein SBI | 0.003248 | 2.5 |
| SAUSA300_2372 | bioA | adenosylmethionine-8-amino-7-oxononanoate transaminase | 0.034567 | 2.2 |
| SAUSA300_2373 | bioD | dethiobiotin synthase | 0.000289 | 4.3 |
| SAUSA300_2384 | . | putative Na+/H+ antiporter | 0.000000 | 2.0 |
| SAUSA300_2385 | . | putative membrane protein | 0.000000 | 3.0 |
| SAUSA300_2387 | . | NAD dependent epimerase/dehydratase family protein | 0.000000 | 2.3 |
| SAUSA300_2388 | panE | 2-dehydropantoate 2-reductase | 0.000000 | 2.5 |
| SAUSA300_2395 | . | amino acid permease | 0.000000 | 4.5 |
| SAUSA300_2397 | . | putative transport protein | 0.000000 | 2.4 |
| SAUSA300_2401 | . | addiction module toxin, Txe/YoeB family | 0.000000 | 2.0 |
| SAUSA300_2402 | . | conserved hypothetical protein | 0.000000 | 2.3 |
| SAUSA300_2420 | . | conserved hypothetical protein | 0.000000 | 2.5 |
| SAUSA300_2437 | sarT | staphylococcal accessory regulator T | 0.001470 | 2.2 |
| SAUSA300_2438 | sarU | staphylococcal accessory regulator U | 0.315026 | 2.1 |
| SAUSA300_2440 | fnbB | fibronectin binding protein B | 0.000000 | 15.8 |
| SAUSA300_2441 | fnbA | fibronectin binding protein A | 0.000000 | 3.0 |
| SAUSA300_2443 | gntK | gluconate kinase | 0.000000 | 4.5 |
| SAUSA300_2444 | gntR | gluconate operon transcriptional repressor | 0.000000 | 5.3 |
| SAUSA300_2448 | . | putative membrane protein | 0.000000 | 2.9 |
| SAUSA300_2452 | . | transcriptional regulator, MarR family | 0.000258 | 2.6 |
| SAUSA300_2453 | . | ABC transporter, ATP-binding protein | 0.000000 | 7.0 |
| SAUSA300_2454 | . | membrane spanning protein | 0.000000 | 5.4 |
| SAUSA300_2476 | ptsG | phosphotransferase system, glucose-specific IIABC component | 0.000000 | 5.7 |
| SAUSA300_2479 | cidA | Holin-like protein cidA | 0.000000 | 8.9 |
| SAUSA300_2482 | . | conserved hypothetical protein | 0.000000 | 5.0 |
| SAUSA300_2489 | . | antibiotic transport-associated protein-like protein | 0.000000 | 3.2 |
| SAUSA300_2503 | . | secretory antigen precursor SsaA | 0.000000 | 4.1 |
| SAUSA300_2504 | . | acyltransferase | 0.000000 | 3.7 |
| SAUSA300_2505 | . | acetyltransferase, GNAT family | 0.000000 | 5.3 |
| SAUSA300_2506 | isaA | immunodominant staphylococcal antigen A precursor | 0.000000 | 2.6 |
| SAUSA300_2507 | . | regulatory protein-like protein | 0.001171 | 2.2 |
| SAUSA300_2508 | . | conserved hypothetical protein | 0.001548 | 7.9 |
| SAUSA300_2509 | . | transcriptional regulator, TetR family | 0.000000 | 2.4 |
| SAUSA300_2511 | . | conserved hypothetical protein | 0.000000 | 2.2 |
| SAUSA300_2512 | . | glyoxalase family protein | 0.000002 | 2.1 |
| SAUSA300_2515 | . | transcriptional regulator, TetR family | 0.000720 | 2.0 |
| SAUSA300_2519 | . | putative cobalamin synthesis protein | 0.000000 | 2.5 |
| SAUSA300_2520 | . | transporter gate domain protein | 0.000000 | 4.0 |
| SAUSA300_2521 | . | conserved hypothetical protein | 0.000068 | 3.5 |
| SAUSA300_2526 | pyrD | dihydroorotate dehydrogenase | 0.000000 | 4.2 |
| SAUSA300_2527 | . | conserved hypothetical protein | 0.000000 | 2.4 |
| SAUSA300_2528 | . | conserved hypothetical protein | 0.000000 | 2.5 |
| SAUSA300_2532 | panD | aspartate 1-decarboxylase | 0.000000 | 2.6 |
| SAUSA300_2533 | panC | pantoate--beta-alanine ligase | 0.000000 | 2.5 |
| SAUSA300_2534 | panB | 3-methyl-2-oxobutanoate hydroxymethyltransferase | 0.000000 | 2.3 |
| SAUSA300_2561 | phoB | alkaline phosphatase | 0.000000 | 7.3 |
| SAUSA300_2564 | estA | tributyrin esterase | 0.000000 | 2.6 |
| SAUSA300_2565 | clfB | clumping factor B | 0.000000 | 5.0 |
| SAUSA300_2572 | aur | zinc metalloproteinase aureolysin | 0.019133 | 2.0 |
| SAUSA300_2588 | . | preprotein translocase, SecY protein | 0.000030 | 2.2 |
| SAUSA300_2591 | . | conserved hypothetical protein | 0.000126 | 2.3 |
| SAUSA300_2595 | . | acetyltransferase, GNAT family | 0.001586 | 2.0 |
| SAUSA300_2596 | cap1C | capsular polysaccharide biosynthesis protein Cap1C | 0.000000 | 3.7 |
| SAUSA300_2597 | cap1B | capsular polysaccharide biosynthesis protein Cap1B | 0.000000 | 5.3 |
| SAUSA300_2598 | cap1A | capsular polysaccharide biosynthesis protein Cap1A | 0.000001 | 8.6 |
| SAUSA300_2600 | icaA | intercellular adhesion protein A | 0.000000 | 5.1 |
| SAUSA300_2601 | icaB | intercellular adhesion protein B | 0.000217 | 3.2 |
| SAUSA300_2602 | icaC | intercellular adhesion protein C | 0.000000 | 3.3 |
| SAUSA300_2609 | hisB | imidazole glycerol phosphate dehydratase hisB | 0.000132 | 3.8 |
| SAUSA300_2610 | hisC | histidinol-phosphate aminotransferase hisC | 0.000210 | 3.4 |
| SAUSA300_2611 | hisD | histidinol dehydrogenase hisD | 0.000000 | 5.2 |
| SAUSA300_2612 | hisG | ATP phosphoribosyltransferase hisG | 0.010141 | 3.5 |
| SAUSA300_2613 | . | conserved hypothetical protein | 0.000054 | 4.7 |
| SAUSA300_2622 | . | conserved hypothetical protein | 0.000000 | 2.5 |
| SAUSA300_2624 | . | putative membrane protein | 0.000000 | 6.1 |
| SAUSA300_2625 | . | transcriptional regulator, PadR family | 0.000000 | 9.7 |
| SAUSA300_2633 | . | ABC transporter, ATP-binding protein | 0.009739 | 2.0 |
| SAUSA300_2635 | . | conserved hypothetical protein | 0.000000 | 4.8 |
| SAUSA300_2637 | . | conserved hypothetical protein | 0.000012 | 2.8 |
| SAUSA300_2638 | . | conserved hypothetical protein | 0.000000 | 5.2 |
| SAUSA300_2639 | . | cold shock protein | 0.000005 | 2.3 |
| SAUSA300_2643 | . | putative chromosome partioning protein, ParB family | 0.000000 | 4.2 |
| SAUSA300_2644 | gidB | glucose-inhibited division protein B | 0.000000 | 7.0 |
| SAUSA300_2645 | gidA | glucose-inhibited division protein A | 0.000000 | 6.7 |
| SAUSA300_2646 | trmE | tRNA modification GTPase | 0.000000 | 7.9 |
| SAUSA300_2647 | rnpA | ribonuclease P protein component | 0.000000 | 10.0 |
| SAUSA300_2648 | rpmH | 50S ribosomal protein L34 | 0.000000 | 4.2 |

**Supplementary Table 4. Genes down-regulated by floxuridine** (Green color, the genes down-regulated by streptozotocin)

| **Gene ID** | **Name** | **Product** | **p-value** | **Ratio** |
| --- | --- | --- | --- | --- |
| SAUSA300_0008 | hutH | histidine ammonia-lyase | 0.000000 | 0.37 |
| SAUSA300_0030 | . | putative glycerophosphoryl diester phosphodiesterase | 0.000000 | 0.48 |
| SAUSA300_0055 | . | alcohol dehydrogenase, zinc-containing | 0.000000 | 0.28 |
| SAUSA300_0065 | arcA | arginine deiminase | 0.000000 | 0.46 |
| SAUSA300_0067 | . | universal stress protein family | 0.000000 | 0.03 |
| SAUSA300_0070 | . | putative lysophospholipase | 0.000000 | 0.06 |
| SAUSA300_0087 | . | . | 0.000000 | 0.23 |
| SAUSA300_0099 | plc | 1-phosphatidylinositol phosphodiesterase | 0.000000 | 0.45 |
| SAUSA300_0100 | . | staphylococcal tandem lipoprotein | 0.000000 | 0.43 |
| SAUSA300_0105 | . | peptidase, M20/M25/M40 family | 0.000000 | 0.32 |
| SAUSA300_0106 | . | putative drug transporter | 0.000000 | 0.36 |
| SAUSA300_0108 | . | antigen, 67 kDa | 0.000000 | 0.05 |
| SAUSA300_0111 | . | conserved hypothetical protein | 0.000000 | 0.12 |
| SAUSA300_0115 | sirC | iron compound ABC transporter, permease protein SirC | 0.000000 | 0.11 |
| SAUSA300_0127 | . | conserved hypothetical protein | 0.000163 | 0.48 |
| SAUSA300_0129 | . | Acetoin(diacetyl) reductase | 0.000000 | 0.12 |
| SAUSA300_0136 | . | cell wall surface anchor family protein | 0.000000 | 0.37 |
| SAUSA300_0138 | deoD | purine nucleoside phosphorylase | 0.215662 | 0.34 |
| SAUSA300_0141 | deoB | phosphopentomutase | 0.000000 | 0.42 |
| SAUSA300_0152 | cap5A | capsular polysaccharide biosynthesis protein Cap5A | 0.000000 | 0.32 |
| SAUSA300_0153 | cap5B | capsular polysaccharide biosynthesis protein Cap5B | 0.000000 | 0.31 |
| SAUSA300_0154 | cap5C | capsular polysaccharide biosynthesis protein Cap5C | 0.000000 | 0.30 |
| SAUSA300_0155 | . | . | 0.000000 | 0.41 |
| SAUSA300_0156 | cap5E | capsular polysaccharide biosynthesis protein Cap5E | 0.000000 | 0.34 |
| SAUSA300_0157 | cap5F | capsular polysaccharide biosynthesis protein Cap5F | 0.000000 | 0.31 |
| SAUSA300_0158 | cap5G | capsular polysaccharide biosynthesis protein Cap5G | 0.000000 | 0.32 |
| SAUSA300_0159 | cap5H | capsular polysaccharide biosynthesis protein Cap5H | 0.000000 | 0.35 |
| SAUSA300_0160 | cap5I | capsular polysaccharide biosynthesis protein Cap5I | 0.000000 | 0.28 |
| SAUSA300_0162 | cap5K | capsular polysaccharide biosynthesis protein Cap5K | 0.000114 | 0.46 |
| SAUSA300_0170 | . | aldehyde dehydrogenase | 0.000000 | 0.12 |
| SAUSA300_0171 | . | cation efflux family protein | 0.000000 | 0.32 |
| SAUSA300_0172 | . | conserved hypothetical protein | 0.000000 | 0.04 |
| SAUSA300_0183 | . | conserved hypothetical protein | 0.000000 | 0.27 |
| SAUSA300_0189 | entB | isochorismatase | 0.000000 | 0.19 |
| SAUSA300_0190 | ipdC | indole-3-pyruvate decarboxylase | 0.000000 | 0.19 |
| SAUSA300_0192 | . | conserved hypothetical protein | 0.000000 | 0.08 |
| SAUSA300_0193 | . | conserved hypothetical protein | 0.000000 | 0.09 |
| SAUSA300_0194 | . | sucrose-specific PTS tranporter protein | 0.000000 | 0.10 |
| SAUSA300_0195 | . | RpiR family transcriptional regulator | 0.000000 | 0.09 |
| SAUSA300_0210 | . | maltose ABC transporter, permease protein | 0.000000 | 0.23 |
| SAUSA300_0211 | . | maltose ABC transporter, permease protein | 0.000000 | 0.23 |
| SAUSA300_0212 | . | oxidoreductase, Gfo/Idh/MocA family | 0.000000 | 0.16 |
| SAUSA300_0213 | . | oxidoreductase, Gfo/Idh/MocA family | 0.000000 | 0.17 |
| SAUSA300_0214 | . | conserved hypothetical protein | 0.000000 | 0.14 |
| SAUSA300_0220 | pflB | formate acetyltransferase | 0.000000 | 0.13 |
| SAUSA300_0221 | pflA | pyruvate formate-lyase activating enzyme | 0.000000 | 0.11 |
| SAUSA300_0226 | . | 3-hydroxyacyl-CoA dehydrogenase | 0.000007 | 0.30 |
| SAUSA300_0227 | fadD | acyl-CoA dehydrogenase FadD | 0.000000 | 0.08 |
| SAUSA300_0228 | fadE | acyl-CoA synthetase FadE | 0.000000 | 0.23 |
| SAUSA300_0229 | . | putative acyl-CoA transferase FadX | 0.000000 | 0.05 |
| SAUSA300_0235 | . | L-lactate dehydrogenase | 0.000000 | 0.49 |
| SAUSA300_0245 | . | 2-C-methyl-D-erythritol 4-phosphate cytidylyltransferase | 0.000000 | 0.50 |
| SAUSA300_0253 | scdA | ScdA protein | 0.000000 | 0.44 |
| SAUSA300_0256 | . | holin-like protein lrgA | 0.004045 | 0.35 |
| SAUSA300_0257 | . | Antiholin-like protein lrgB | 0.000000 | 0.22 |
| SAUSA300_0258 | . | transcriptional regulator, GntR family | 0.000001 | 0.43 |
| SAUSA300_0259 | . | PTS system, IIA component | 0.000000 | 0.25 |
| SAUSA300_0260 | bglA | 6-phospho-beta-glucosidase | 0.000000 | 0.19 |
| SAUSA300_0262 | rbsK | ribokinase | 0.000000 | 0.45 |
| SAUSA300_0264 | . | ribose transporter RbsU | 0.000000 | 0.42 |
| SAUSA300_0265 | . | putative ribose operon repressor | 0.000000 | 0.23 |
| SAUSA300_0278 | . | conserved hypothetical protein | 0.000000 | 0.14 |
| SAUSA300_0308 | . | ABC transporter, permease protein | 0.000000 | 0.06 |
| SAUSA300_0309 | . | ABC transporter ATP-binding protein | 0.000000 | 0.07 |
| SAUSA300_0311 | . | PfkB family carbohydrate kinase | 0.000173 | 0.40 |
| SAUSA300_0314 | . | sodium:solute symporter family protein | 0.000000 | 0.42 |
| SAUSA300_0315 | nanA | N-acetylneuraminate lyase subunit | 0.000000 | 0.30 |
| SAUSA300_0316 | . | ROK family protein | 0.000000 | 0.48 |
| SAUSA300_0320 | . | triacylglycerol lipase precursor | 0.000000 | 0.25 |
| SAUSA300_0329 | . | putative oxidoreductase | 0.000000 | 0.09 |
| SAUSA300_0330 | . | putative transport protein SgaT | 0.000000 | 0.07 |
| SAUSA300_0331 | . | conserved hypothetical protein | 0.000000 | 0.08 |
| SAUSA300_0332 | . | PTS system, IIA component | 0.000000 | 0.10 |
| SAUSA300_0333 | . | transcriptional antiterminator, BglG family | 0.000000 | 0.17 |
| SAUSA300_0338 | . | glyoxalase family protein | 0.000000 | 0.09 |
| SAUSA300_0339 | . | conserved hypothetical protein | 0.000000 | 0.10 |
| SAUSA300_0340 | . | NADH-dependent FMN reductase | 0.000000 | 0.11 |
| SAUSA300_0342 | . | conserved hypothetical protein | 0.000274 | 0.38 |
| SAUSA300_0372 | . | putative lipoprotein | 0.000000 | 0.05 |
| SAUSA300_0374 | . | putative membrane protein | 0.000000 | 0.03 |
| SAUSA300_0385 | . | conserved hypothetical protein | 0.000000 | 0.04 |
| SAUSA300_0391 | . | conserved hypothetical protein | 0.000000 | 0.41 |
| SAUSA300_0393 | . | conserved hypothetical protein | 0.000000 | 0.25 |
| SAUSA300_0394 | . | FAD/NAD(P)-binding Rossmann fold Superfamily | 0.000000 | 0.26 |
| SAUSA300_0395 | . | exotoxin | 0.000000 | 0.29 |
| SAUSA300_0409 | . | conserved hypothetical protein | 0.000000 | 0.26 |
| SAUSA300_0410 | . | staphylococcal tandem lipoprotein | 0.003958 | 0.44 |
| SAUSA300_0411 | . | staphylococcal tandem lipoprotein | 0.003494 | 0.49 |
| SAUSA300_0413 | . | staphylococcal tandem lipoprotein | 0.001297 | 0.40 |
| SAUSA300_0415 | lpl3 | staphylococcal tandem lipoprotein | 0.014324 | 0.50 |
| SAUSA300_0416 | . | staphylococcal tandem lipoprotein | 0.024368 | 0.44 |
| SAUSA300_0417 | . | staphylococcal tandem lipoprotein | 0.000000 | 0.38 |
| SAUSA300_0418 | . | staphylococcal tandem lipoprotein | 0.000000 | 0.43 |
| SAUSA300_0419 | . | staphylococcal tandem lipoprotein | 0.000000 | 0.18 |
| SAUSA300_0420 | . | conserved hypothetical protein | 0.000000 | 0.22 |
| SAUSA300_0421 | . | conserved hypothetical protein | 0.000000 | 0.29 |
| SAUSA300_0422 | . | conserved hypothetical protein | 0.000000 | 0.25 |
| SAUSA300_0426 | . | conserved hypothetical protein | 0.000000 | 0.42 |
| SAUSA300_0427 | . | conserved hypothetical protein | 0.000000 | 0.46 |
| SAUSA300_0455 | rrsA | 16S ribosomal RNA | 0.001111 | 0.44 |
| SAUSA300_0471 | . | veg protein | 0.000000 | 0.34 |
| SAUSA300_0474 | . | putative endoribonuclease L-PSP | 0.000000 | 0.13 |
| SAUSA300_0475 | . | SpoVG protein | 0.000000 | 0.12 |
| SAUSA300_0476 | . | hypothetical protein | 0.000001 | 0.08 |
| SAUSA300_0498 | . | tRNA-Ala | 0.003176 | 0.38 |
| SAUSA300_0499 | rrsB | 16S ribosomal RNA | 0.001094 | 0.44 |
| SAUSA300_0514 | cysE | serine acetyltransferase | 0.000000 | 0.38 |
| SAUSA300_0515 | cysS | cysteinyl-tRNA synthetase | 0.000000 | 0.34 |
| SAUSA300_0516 | . | conserved hypothetical protein | 0.000000 | 0.41 |
| SAUSA300_0517 | . | RNA methyltransferase, TrmH family, group 3 | 0.000000 | 0.36 |
| SAUSA300_0518 | . | conserved hypothetical protein | 0.000000 | 0.37 |
| SAUSA300_0519 | . | conserved hypothetical protein | 0.000000 | 0.49 |
| SAUSA300_0535 | . | putative pyridoxal phosphate-dependent acyltransferase | 0.000000 | 0.32 |
| SAUSA300_0536 | . | DJ-1/PfpI family protein | 0.000000 | 0.29 |
| SAUSA300_0537 | . | L-ribulokinase | 0.000000 | 0.41 |
| SAUSA300_0555 | . | putative hexulose-6-phosphate synthase | 0.000000 | 0.36 |
| SAUSA300_0556 | . | SIS domain protein | 0.000000 | 0.32 |
| SAUSA300_0566 | . | amino acid permease | 0.000000 | 0.24 |
| SAUSA300_0573 | mvaD | diphosphomevalonate decarboxylase | 0.000000 | 0.32 |
| SAUSA300_0574 | . | phosphomevalonate kinase | 0.000000 | 0.38 |
| SAUSA300_0590 | . | conserved hypothetical protein | 0.000000 | 0.48 |
| SAUSA300_0594 | adh | alcohol dehydrogenase | 0.000000 | 0.12 |
| SAUSA300_0602 | . | conserved hypothetical protein | 0.000000 | 0.17 |
| SAUSA300_0604 | . | hydrolase, alpha/beta hydrolase fold family | 0.000000 | 0.06 |
| SAUSA300_0605 | sarA | staphylococcal accessory regulator A | 0.000000 | 0.10 |
| SAUSA300_0609 | . | phage integrase family protein | 0.000000 | 0.11 |
| SAUSA300_0610 | . | putative Na+/H+ antiporter, MnhA component | 0.000000 | 0.17 |
| SAUSA300_0611 | . | putative Na+/H+ antiporter, MnhB component | 0.000000 | 0.20 |
| SAUSA300_0612 | . | putative Na+/H+ antiporter, MnhC component | 0.000000 | 0.29 |
| SAUSA300_0613 | . | putative Na+/H+ antiporter, MnhD component | 0.000000 | 0.31 |
| SAUSA300_0614 | . | putative Na+/H+ antiporter, MnhE component | 0.000022 | 0.37 |
| SAUSA300_0615 | . | putative Na+/H+ antiporter, MnhF component | 0.000000 | 0.34 |
| SAUSA300_0616 | . | putative Na+/H+ antiporter, MnhG component | 0.000000 | 0.33 |
| SAUSA300_0630 | . | ABC transporter, ATP-binding protein | 0.000000 | 0.20 |
| SAUSA300_0636 | . | dihydroxyacetone kinase, DhaK subunit | 0.000000 | 0.21 |
| SAUSA300_0637 | . | dihydroxyacetone kinase, DhaL subunit | 0.000000 | 0.21 |
| SAUSA300_0638 | . | dihydroxyacetone kinase, phosphotransfer subunit | 0.000000 | 0.24 |
| SAUSA300_0643 | . | acetyltransferase, GNAT family | 0.000000 | 0.49 |
| SAUSA300_0662 | . | acetyltransferase, GNAT family | 0.000003 | 0.37 |
| SAUSA300_0664 | . | conserved hypothetical protein | 0.000000 | 0.13 |
| SAUSA300_0665 | . | acetyltransferase, GNAT family | 0.000000 | 0.47 |
| SAUSA300_0666 | . | decarboxylase family protein | 0.000000 | 0.15 |
| SAUSA300_0667 | . | YaiI/YqxD family protein | 0.000000 | 0.18 |
| SAUSA300_0668 | . | conserved hypothetical protein | 0.000000 | 0.07 |
| SAUSA300_0677 | . | putative deoxyribodipyrimidine photolyase | 0.000000 | 0.32 |
| SAUSA300_0688 | . | oxidoreductase, aldo/keto reductase family | 0.000000 | 0.07 |
| SAUSA300_0689 | . | glycosyl transferase, group 2 family protein | 0.000000 | 0.07 |
| SAUSA300_0706 | . | putative osmoprotectant ABC transporter, ATP-binding protein | 0.000000 | 0.35 |
| SAUSA300_0707 | . | osmoprotectant ABC transporter, permease | 0.000000 | 0.46 |
| SAUSA300_0711 | . | conserved hypothetical protein | 0.000000 | 0.05 |
| SAUSA300_0736 | yfiA | ribosomal subunit interface protein | 0.000000 | 0.10 |
| SAUSA300_0749 | . | conserved hypothetical protein | 0.000000 | 0.09 |
| SAUSA300_0750 | . | conserved hypothetical protein | 0.000000 | 0.13 |
| SAUSA300_0751 | . | tRNA-Arg | 0.000294 | 0.37 |
| SAUSA300_0753 | . | conserved hypothetical protein | 0.000000 | 0.08 |
| SAUSA300_0754 | . | conserved hypothetical protein | 0.000000 | 0.32 |
| SAUSA300_0760 | eno | phosphopyruvate hydratase | 0.000000 | 0.47 |
| SAUSA300_0766 | . | . | 0.000000 | 0.36 |
| SAUSA300_0767 | . | conserved hypothetical protein | 0.000000 | 0.22 |
| SAUSA300_0768 | . | conserved hypothetical protein | 0.000000 | 0.23 |
| SAUSA300_0769 | . | putative lipoprotein | 0.000000 | 0.16 |
| SAUSA300_0770 | . | conserved hypothetical protein | 0.000000 | 0.08 |
| SAUSA300_0771 | . | acetyltransferase, GNAT family | 0.000000 | 0.43 |
| SAUSA300_0772 | clfA | clumping factor A | 0.000000 | 0.03 |
| SAUSA300_0778 | . | conserved hypothetical protein | 0.000000 | 0.23 |
| SAUSA300_0779 | . | conserved hypothetical protein | 0.000000 | 0.28 |
| SAUSA300_0781 | . | conserved hypothetical protein | 0.000000 | 0.05 |
| SAUSA300_0784 | . | LysE/YggA family protein | 0.000000 | 0.44 |
| SAUSA300_0785 | . | acetyltransferase, GNAT family | 0.000000 | 0.48 |
| SAUSA300_0786 | . | OsmC/Ohr family protein | 0.000000 | 0.03 |
| SAUSA300_0791 | gcvH | glycine cleavage system H protein | 0.000000 | 0.44 |
| SAUSA300_0794 | . | Toprim domain protein | 0.000000 | 0.10 |
| SAUSA300_0795 | . | putative thioredoxin | 0.000000 | 0.08 |
| SAUSA300_0816 | . | CsbD-like superfamily | 0.000000 | 0.02 |
| SAUSA300_0818 | sufC | FeS assembly ATPase SufC | 0.000000 | 0.37 |
| SAUSA300_0819 | sufD | FeS assembly protein SufD | 0.000000 | 0.47 |
| SAUSA300_0824 | . | conserved hypothetical protein | 0.000000 | 0.22 |
| SAUSA300_0825 | . | oxidoreductase, 2-nitropropane dioxygenase family | 0.000000 | 0.49 |
| SAUSA300_0840 | . | conserved hypothetical protein | 0.000000 | 0.42 |
| SAUSA300_0844 | . | conserved hypothetical protein | 0.000000 | 0.36 |
| SAUSA300_0858 | . | conserved hypothetical protein | 0.000000 | 0.43 |
| SAUSA300_0860 | rocD | Ornithine aminotransferase | 0.000000 | 0.15 |
| SAUSA300_0861 | gudB | NAD-specific glutamate dehydrogenase | 0.000000 | 0.15 |
| SAUSA300_0862 | glpQ | glycerophosphoryl diester phosphodiesterase | 0.000000 | 0.21 |
| SAUSA300_0871 | . | conserved hypothetical protein | 0.000000 | 0.49 |
| SAUSA300_0877 | clpB | Chaperone clpB | 0.000000 | 0.30 |
| SAUSA300_0883 | . | putative surface protein | 0.000000 | 0.04 |
| SAUSA300_0885 | fabH | 3-oxoacyl-(acyl-carrier-protein) synthase III | 0.000000 | 0.25 |
| SAUSA300_0887 | oppB | oligopeptide ABC transporter, permease protein | 0.000000 | 0.31 |
| SAUSA300_0888 | oppC | oligopeptide ABC transporter, permease protein | 0.000000 | 0.31 |
| SAUSA300_0889 | oppD | oligopeptide ABC transporter, ATP-binding protein | 0.000000 | 0.34 |
| SAUSA300_0890 | oppF | oligopeptide ABC transporter, ATP-binding protein | 0.000000 | 0.27 |
| SAUSA300_0891 | oppA | oligopeptide ABC transporter, substrate-binding protein | 0.000000 | 0.26 |
| SAUSA300_0893 | oppF | oligopeptide ABC transporter, ATP-binding protein | 0.000129 | 0.30 |
| SAUSA300_0894 | oppD | oligopeptide ABC transporter, ATP-binding protein | 0.012834 | 0.47 |
| SAUSA300_0898 | spxA | Regulatory protein spx | 0.000000 | 0.28 |
| SAUSA300_0903 | . | conserved hypothetical protein | 0.000000 | 0.45 |
| SAUSA300_0904 | . | protozoan/cyanobacterial globin family protein | 0.000000 | 0.40 |
| SAUSA300_0916 | . | conserved hypothetical protein | 0.000000 | 0.28 |
| SAUSA300_0929 | . | conserved hypothetical protein | 0.000000 | 0.17 |
| SAUSA300_0933 | . | conserved hypothetical protein | 0.000000 | 0.09 |
| SAUSA300_0949 | sspC | cysteine protease | 0.000000 | 0.18 |
| SAUSA300_0950 | sspB | cysteine protease precursor | 0.000000 | 0.21 |
| SAUSA300_0951 | sspA | V8 protease | 0.000000 | 0.18 |
| SAUSA300_0955 | atl | autolysin | 0.000000 | 0.28 |
| SAUSA300_0960 | qoxD | quinol oxidase, subunit IV | 0.000000 | 0.21 |
| SAUSA300_0961 | qoxC | quinol oxidase, subunit III | 0.000000 | 0.23 |
| SAUSA300_0962 | qoxB | quinol oxidase, subunit I | 0.000000 | 0.25 |
| SAUSA300_0963 | qoxA | quinol oxidase, subunit II | 0.000000 | 0.24 |
| SAUSA300_0965 | folD | methylenetetrahydrofolate dehydrogenase/methenyltetrahydrofolate cyclohydrolase | 0.000000 | 0.18 |
| SAUSA300_0966 | purE | phosphoribosylaminoimidazole carboxylase, catalytic subunit | 0.000038 | 0.46 |
| SAUSA300_0969 | purS | phosphoribosylformylglycinamidine synthase | 0.003148 | 0.48 |
| SAUSA300_0970 | purQ | phosphoribosylformylglycinamidine synthase I | 0.000013 | 0.43 |
| SAUSA300_0971 | purL | phosphoribosylformylglycinamidine synthase II | 0.000000 | 0.43 |
| SAUSA300_0972 | purF | amidophosphoribosyltransferase | 0.000000 | 0.49 |
| SAUSA300_0973 | purM | phosphoribosylformylglycinamidine cyclo-ligase | 0.000000 | 0.47 |
| SAUSA300_0980 | . | putative membrane protein | 0.000000 | 0.29 |
| SAUSA300_0982 | . | conserved hypothetical protein | 0.000000 | 0.08 |
| SAUSA300_0983 | ptsH | phosphocarrier protein HPr | 0.000000 | 0.36 |
| SAUSA300_0984 | ptsI | phosphoenolpyruvate-protein phosphotransferase | 0.000000 | 0.37 |
| SAUSA300_1002 | potD | spermidine/putrescine ABC transporter, spermidine/putrescine-binding protein | 0.000000 | 0.35 |
| SAUSA300_1003 | . | conserved hypothetical protein | 0.000000 | 0.50 |
| SAUSA300_1007 | . | inositol monophosphatase family protein | 0.000000 | 0.40 |
| SAUSA300_1008 | . | conserved hypothetical protein | 0.000000 | 0.39 |
| SAUSA300_1012 | . | conserved hypothetical protein | 0.000000 | 0.42 |
| SAUSA300_1044 | trx | thioredoxin | 0.000000 | 0.34 |
| SAUSA300_1045 | uvrC | excinuclease ABC, C subunit | 0.000000 | 0.47 |
| SAUSA300_1046 | sdhC | succinate dehydrogenase, cytochrome b-558 subunit | 0.000000 | 0.45 |
| SAUSA300_1047 | sdhA | succinate dehydrogenase, flavoprotein subunit | 0.000000 | 0.33 |
| SAUSA300_1048 | sdhB | succinate dehydrogenase, iron-sulfur protein | 0.000000 | 0.26 |
| SAUSA300_1052 | . | fibrinogen-binding protein | 0.000000 | 0.31 |
| SAUSA300_1053 | . | conserved hypothetical protein | 0.003010 | 0.35 |
| SAUSA300_1055 | efb | fibrinogen-binding protein | 0.000137 | 0.43 |
| SAUSA300_1056 | . | conserved hypothetical protein | 0.000706 | 0.47 |
| SAUSA300_1058 | . | alpha-hemolysin precursor | 0.000000 | 0.05 |
| SAUSA300_1064 | . | transporter, TRAP family | 0.000000 | 0.35 |
| SAUSA300_1070 | . | acetyltransferase, GNAT family | 0.000000 | 0.30 |
| SAUSA300_1072 | mraZ | protein mraZ | 0.000000 | 0.08 |
| SAUSA300_1073 | mraW | S-adenosyl-methyltransferase MraW | 0.000000 | 0.08 |
| SAUSA300_1074 | ftsL | cell division protein | 0.000000 | 0.17 |
| SAUSA300_1075 | pbpA | penicillin-binding protein 1 | 0.000000 | 0.36 |
| SAUSA300_1076 | mraY | phospho-N-acetylmuramoyl-pentapeptide- transferase | 0.000000 | 0.36 |
| SAUSA300_1077 | murD | UDP-N-acetylmuramoylalanine--D-glutamate ligase | 0.000000 | 0.31 |
| SAUSA300_1078 | divIB | cell division protein | 0.000000 | 0.36 |
| SAUSA300_1079 | ftsA | cell division protein ftsA | 0.000000 | 0.47 |
| SAUSA300_1100 | . | conserved hypothetical protein | 0.000000 | 0.34 |
| SAUSA300_1106 | . | putative lipoprotein | 0.000003 | 0.48 |
| SAUSA300_1107 | . | conserved hypothetical protein | 0.000000 | 0.06 |
| SAUSA300_1117 | rpmB | 50S ribosomal protein L28 | 0.000000 | 0.16 |
| SAUSA300_1121 | . | conserved hypothetical protein | 0.000000 | 0.33 |
| SAUSA300_1122 | plsX | fatty acid/phospholipid synthesis protein PlsX | 0.000000 | 0.41 |
| SAUSA300_1123 | fabD | malonyl CoA-acyl carrier protein transacylase | 0.000000 | 0.49 |
| SAUSA300_1138 | sucC | succinyl-CoA synthetase, beta subunit | 0.000000 | 0.30 |
| SAUSA300_1139 | sucD | succinyl-CoA synthetase, alpha subunit | 0.000000 | 0.20 |
| SAUSA300_1145 | xerC | tyrosine recombinase xerC | 0.000000 | 0.32 |
| SAUSA300_1146 | hslV | ATP-dependent protease hslV | 0.000000 | 0.39 |
| SAUSA300_1147 | hslU | heat shock protein HslVU, ATPase subunit HslU | 0.000000 | 0.40 |
| SAUSA300_1148 | codY | GTP-sensing transcriptional pleiotropic repressor CodY | 0.000000 | 0.36 |
| SAUSA300_1164 | truB | tRNA pseudouridine synthase B | 0.000000 | 0.44 |
| SAUSA300_1165 | ribF | riboflavin biosynthesis protein ribF | 0.000000 | 0.50 |
| SAUSA300_1179 | . | conserved hypothetical protein | 0.000000 | 0.35 |
| SAUSA300_1180 | . | conserved hypothetical protein | 0.000000 | 0.11 |
| SAUSA300_1191 | glpF | glycerol uptake facilitator | 0.000000 | 0.10 |
| SAUSA300_1192 | glpK | glycerol kinase | 0.000000 | 0.31 |
| SAUSA300_1193 | glpD | glycerol-3-phosphate dehydrogenase | 0.000000 | 0.27 |
| SAUSA300_1197 | . | glutathione peroxidase | 0.000000 | 0.35 |
| SAUSA300_1211 | . | conserved hypothetical protein | 0.000000 | 0.40 |
| SAUSA300_1212 | . | conserved hypothetical protein | 0.000179 | 0.47 |
| SAUSA300_1213 | . | conserved hypothetical protein | 0.000362 | 0.45 |
| SAUSA300_1215 | . | conserved hypothetical protein | 0.146578 | 0.41 |
| SAUSA300_1221 | . | conserved hypothetical protein | 0.045834 | 0.33 |
| SAUSA300_1224 | . | conserved hypothetical protein | 0.000000 | 0.47 |
| SAUSA300_1225 | . | aspartate kinase | 0.000000 | 0.09 |
| SAUSA300_1226 | . | homoserine dehydrogenase | 0.000000 | 0.03 |
| SAUSA300_1227 | thrC | threonine synthase | 0.000000 | 0.04 |
| SAUSA300_1228 | thrB | homoserine kinase | 0.000000 | 0.04 |
| SAUSA300_1229 | . | hydrolase, haloacid dehalogenase-like family | 0.000000 | 0.21 |
| SAUSA300_1232 | . | catalase | 0.000000 | 0.10 |
| SAUSA300_1233 | rpmG | 50S ribosomal protein L33 | 0.000000 | 0.28 |
| SAUSA300_1235 | guaC | guanosine monophosphate reductase | 0.024097 | 0.44 |
| SAUSA300_1236 | . | conserved hypothetical protein | 0.000000 | 0.24 |
| SAUSA300_1246 | acnA | aconitate hydratase | 0.000000 | 0.42 |
| SAUSA300_1248 | . | conserved hypothetical protein | 0.000000 | 0.48 |
| SAUSA300_1255 | fmtC | oxacillin resistance-related FmtC protein | 0.000000 | 0.48 |
| SAUSA300_1258 | . | 4-oxalocrotonate tautomerase | 0.000000 | 0.37 |
| SAUSA300_1260 | . | prephenate dehydrogenase | 0.000000 | 0.09 |
| SAUSA300_1267 | trpB | tryptophan synthase, beta subunit | 0.004757 | 0.48 |
| SAUSA300_1269 | femA | methicillin resistance protein FemA | 0.000000 | 0.36 |
| SAUSA300_1270 | femB | methicillin resistance protein FemB | 0.000000 | 0.45 |
| SAUSA300_1272 | . | conserved hypothetical protein | 0.000000 | 0.39 |
| SAUSA300_1273 | opp-2F | oligopeptide permease, ATP-binding protein | 0.000000 | 0.37 |
| SAUSA300_1277 | . | conserved hypothetical protein | 0.000000 | 0.39 |
| SAUSA300_1279 | phoU | phosphate transport system regulatory protein PhoU | 0.000000 | 0.44 |
| SAUSA300_1280 | pstB | phosphate ABC transporter, ATP-binding protein | 0.000000 | 0.23 |
| SAUSA300_1281 | pstA | phosphate ABC transporter, permease protein PstA | 0.000000 | 0.21 |
| SAUSA300_1283 | pstS | phosphate ABC transporter, phosphate-binding protein PstS | 0.039846 | 0.46 |
| SAUSA300_1287 | asd | aspartate semialdehyde dehydrogenase | 0.000000 | 0.24 |
| SAUSA300_1288 | dapA | dihydrodipicolinate synthase | 0.000000 | 0.26 |
| SAUSA300_1289 | dapB | dihydrodipicolinate reductase | 0.000000 | 0.26 |
| SAUSA300_1290 | dapD | tetrahydrodipicolinate acetyltransferase | 0.000000 | 0.18 |
| SAUSA300_1297 | . | conserved hypothetical protein | 0.000000 | 0.40 |
| SAUSA300_1298 | . | putative XpaC protein | 0.000000 | 0.44 |
| SAUSA300_1299 | . | putative tellurite resistance protein | 0.000000 | 0.47 |
| SAUSA300_1303 | . | conserved hypothetical protein | 0.000000 | 0.26 |
| SAUSA300_1304 | . | conserved hypothetical protein | 0.000000 | 0.23 |
| SAUSA300_1305 | sucB | 2-oxoglutarate dehydrogenase, E2 component, dihydrolipoamide succinyltransferase | 0.000000 | 0.05 |
| SAUSA300_1306 | sucA | 2-oxoglutarate dehydrogenase, E1 component | 0.000000 | 0.05 |
| SAUSA300_1307 | arlS | sensor histidine kinase protein | 0.000000 | 0.27 |
| SAUSA300_1308 | arlR | DNA-binding response regulator | 0.000000 | 0.18 |
| SAUSA300_1314 | . | conserved hypothetical protein | 0.000000 | 0.39 |
| SAUSA300_1315 | crr | PTS system, glucose-specific IIA component | 0.000000 | 0.40 |
| SAUSA300_1316 | msrB | methionine-R-sulfoxide reductase | 0.000000 | 0.44 |
| SAUSA300_1317 | msrA | methionine-S-sulfoxide reductase | 0.000000 | 0.33 |
| SAUSA300_1318 | . | DegV family protein | 0.000000 | 0.38 |
| SAUSA300_1319 | folA | dihydrofolate reductase | 0.000000 | 0.35 |
| SAUSA300_1320 | thyA | thymidylate synthase | 0.000000 | 0.30 |
| SAUSA300_1325 | . | conserved hypothetical protein | 0.000279 | 0.33 |
| SAUSA300_1327 | . | cell surface protein | 0.000000 | 0.36 |
| SAUSA300_1337 | . | conserved hypothetical protein | 0.000000 | 0.33 |
| SAUSA300_1338 | . | conserved hypothetical protein | 0.000000 | 0.20 |
| SAUSA300_1339 | . | conserved hypothetical protein | 0.000000 | 0.31 |
| SAUSA300_1340 | recU | recombination protein U | 0.000075 | 0.48 |
| SAUSA300_1362 | hup | DNA-binding protein HU | 0.000000 | 0.08 |
| SAUSA300_1365 | rpsA | 30S ribosomal protein S1 | 0.000000 | 0.10 |
| SAUSA300_1370 | ebpS | cell surface elastin binding protein | 0.000000 | 0.13 |
| SAUSA300_1371 | recQ | ATP-dependent DNA helicase RecQ | 0.000000 | 0.30 |
| SAUSA300_1392 | . | phiSLT ORF191-like protein | 0.000000 | 0.24 |
| SAUSA300_1393 | . | phiSLT ORF2067-like protein, phage tail tape measure protein | 0.000000 | 0.25 |
| SAUSA300_1394 | . | conserved hypothetical phage protein | 0.060213 | 0.16 |
| SAUSA300_1395 | . | phiSLT ORF116b-like protein | 0.007479 | 0.06 |
| SAUSA300_1396 | . | phiSLT ORF151-like protein, major tail protein | 0.000000 | 0.17 |
| SAUSA300_1397 | . | phiSLT ORF213-like protein, major tail protein | 0.000000 | 0.17 |
| SAUSA300_1407 | . | phi SLT ORF 145-like protein, phage transcriptional regulator | 0.000002 | 0.36 |
| SAUSA300_1408 | . | phage helicase | 0.000000 | 0.32 |
| SAUSA300_1409 | . | conserved hypothetical phage protein | 0.000000 | 0.25 |
| SAUSA300_1411 | . | phiSLT ORF66-like protein | 0.025923 | 0.37 |
| SAUSA300_1412 | . | phiSLT ORF 50-like protein | 0.205859 | 0.46 |
| SAUSA300_1415 | . | phiSLT ORF 77-like protein | 0.126732 | 0.22 |
| SAUSA300_1418 | . | phiSLT ORF 82-like protein | 0.239861 | 0.30 |
| SAUSA300_1432 | . | phiSLT ORF78-like protein | 0.000000 | 0.13 |
| SAUSA300_1437 | . | phiSLT ORF204-like protein | 0.000000 | 0.28 |
| SAUSA300_1440 | . | conserved hypothetical protein | 0.000017 | 0.43 |
| SAUSA300_1450 | . | oxidoreductase, aldo/keto reductase family | 0.000000 | 0.28 |
| SAUSA300_1456 | . | alpha glucosidase | 0.000000 | 0.21 |
| SAUSA300_1457 | malR | maltose operon transcriptional repressor | 0.000000 | 0.47 |
| SAUSA300_1460 | . | peptidase, M20/M25/M40 family | 0.000000 | 0.30 |
| SAUSA300_1465 | . | 2-oxoisovalerate dehydrogenase, E1 component, beta subunit | 0.000000 | 0.47 |
| SAUSA300_1466 | . | 2-oxoisovalerate dehydrogenase, E1 component, alpha subunit | 0.000000 | 0.49 |
| SAUSA300_1468 | recN | DNA repair protein RecN | 0.000000 | 0.45 |
| SAUSA300_1478 | . | putative lipoprotein | 0.000000 | 0.46 |
| SAUSA300_1479 | . | conserved hypothetical protein | 0.000000 | 0.18 |
| SAUSA300_1480 | . | putative traG membrane protein | 0.000000 | 0.09 |
| SAUSA300_1492 | . | putative lipoprotein | 0.000000 | 0.38 |
| SAUSA300_1499 | aroK | shikimate kinase | 0.000000 | 0.32 |
| SAUSA300_1505 | . | conserved hypothetical protein | 0.000000 | 0.40 |
| SAUSA300_1506 | . | conserved hypothetical protein | 0.000000 | 0.48 |
| SAUSA300_1507 | glk | glucokinase | 0.000000 | 0.47 |
| SAUSA300_1508 | . | conserved hypothetical protein | 0.000000 | 0.42 |
| SAUSA300_1509 | . | peptidase, rhomboid family | 0.000000 | 0.41 |
| SAUSA300_1510 | . | 5-formyltetrahydrofolate cyclo-ligase subfamily | 0.000000 | 0.38 |
| SAUSA300_1513 | . | superoxide dismutase (Mn/Fe family) | 0.000000 | 0.19 |
| SAUSA300_1523 | . | conserved hypothetical protein | 0.000000 | 0.47 |
| SAUSA300_1524 | . | CBS domain pair protein | 0.000000 | 0.46 |
| SAUSA300_1525 | glyS | glycyl-tRNA synthetase | 0.000000 | 0.36 |
| SAUSA300_1527 | era | GTP-binding protein Era | 0.000000 | 0.46 |
| SAUSA300_1528 | cdd | cytidine deaminase | 0.000000 | 0.43 |
| SAUSA300_1529 | dgkA | diacylglycerol kinase | 0.000000 | 0.38 |
| SAUSA300_1530 | . | conserved hypothetical protein | 0.000000 | 0.34 |
| SAUSA300_1531 | phoH | phosphate starvation-induced protein, PhoH family | 0.000000 | 0.25 |
| SAUSA300_1533 | . | conserved hypothetical protein | 0.000000 | 0.46 |
| SAUSA300_1534 | . | conserved hypothetical protein | 0.000000 | 0.41 |
| SAUSA300_1561 | . | putative membrane protein | 0.000000 | 0.48 |
| SAUSA300_1562 | . | LamB/YcsF family protein | 0.000000 | 0.42 |
| SAUSA300_1563 | accC | acetyl-CoA carboxylase, biotin carboxylase | 0.000000 | 0.38 |
| SAUSA300_1564 | accB | acetyl-CoA carboxylase, biotin carboxyl carrier protein | 0.000029 | 0.33 |
| SAUSA300_1565 | . | putative urea amidolyase | 0.000000 | 0.32 |
| SAUSA300_1566 | . | conserved hypothetical protein | 0.000000 | 0.26 |
| SAUSA300_1581 | . | conserved hypothetical protein | 0.000000 | 0.05 |
| SAUSA300_1582 | . | conserved hypothetical protein | 0.000000 | 0.05 |
| SAUSA300_1610 | folC | folylpolyglutamate synthase | 0.000000 | 0.45 |
| SAUSA300_1611 | valS | valyl-tRNA synthetase | 0.000000 | 0.45 |
| SAUSA300_1613 | . | putative abrB protein | 0.000000 | 0.40 |
| SAUSA300_1628 | lysP | lysine-specific permease | 0.000000 | 0.30 |
| SAUSA300_1629 | thrS | threonyl-tRNA synthetase | 0.000000 | 0.13 |
| SAUSA300_1633 | gap | glyceraldehyde-3-phosphate dehydrogenase, type I | 0.000000 | 0.02 |
| SAUSA300_1638 | phoR | sensory box histidine kinase PhoR | 0.000000 | 0.38 |
| SAUSA300_1639 | phoP | alkaline phosphatase synthesis transcriptional regulatory protein PhoP | 0.000000 | 0.41 |
| SAUSA300_1640 | icd | isocitrate dehydrogenase, NADP-dependent | 0.000000 | 0.20 |
| SAUSA300_1641 | gltA | citrate synthase II | 0.000000 | 0.28 |
| SAUSA300_1652 | . | conserved hypothetical protein | 0.000000 | 0.37 |
| SAUSA300_1653 | . | conserved hypothetical protein | 0.000000 | 0.05 |
| SAUSA300_1654 | . | proline dipeptidase | 0.000000 | 0.42 |
| SAUSA300_1655 | ald | alanine dehydrogenase | 0.000000 | 0.13 |
| SAUSA300_1656 | . | universal stress protein family | 0.000000 | 0.06 |
| SAUSA300_1668 | . | OsmC/Ohr family protein | 0.000000 | 0.40 |
| SAUSA300_1678 | fhs | formate-tetrahydrofolate ligase | 0.000000 | 0.25 |
| SAUSA300_1679 | acsA | acetyl-coenzyme A synthetase | 0.000000 | 0.16 |
| SAUSA300_1680 | acuA | acetoin utilization protein AcuA | 0.000000 | 0.11 |
| SAUSA300_1681 | acuC | acetoin utilization protein AcuC | 0.000000 | 0.10 |
| SAUSA300_1682 | ccpA | catabolite control protein A | 0.000000 | 0.41 |
| SAUSA300_1684 | . | conserved hypothetical protein | 0.000000 | 0.10 |
| SAUSA300_1685 | . | conserved hypothetical protein | 0.000000 | 0.10 |
| SAUSA300_1690 | . | putative thioredoxin | 0.000000 | 0.18 |
| SAUSA300_1691 | . | glutamyl-aminopeptidase | 0.000000 | 0.48 |
| SAUSA300_1698 | . | conserved hypothetical protein | 0.000000 | 0.12 |
| SAUSA300_1708 | rot | staphylococcal accessory regulator Rot | 0.000000 | 0.29 |
| SAUSA300_1711 | putA | proline dehydrogenase | 0.000000 | 0.16 |
| SAUSA300_1712 | ribH | riboflavin synthase, beta subunit | 0.000018 | 0.15 |
| SAUSA300_1713 | ribBA | riboflavin biosynthesis protein | 0.000004 | 0.14 |
| SAUSA300_1714 | ribE | riboflavin synthase, alpha subunit | 0.000000 | 0.12 |
| SAUSA300_1715 | ribD | riboflavin biosynthesis protein | 0.000000 | 0.11 |
| SAUSA300_1716 | . | conserved hypothetical protein | 0.000000 | 0.23 |
| SAUSA300_1725 | . | transaldolase | 0.000000 | 0.39 |
| SAUSA300_1728 | . | oxidoreductase, aldo/keto reductase family | 0.000000 | 0.30 |
| SAUSA300_1731 | pckA | phosphoenolpyruvate carboxykinase (ATP) | 0.000000 | 0.04 |
| SAUSA300_1739 | . | conserved hypothetical protein | 0.000000 | 0.07 |
| SAUSA300_1740 | . | conserved hypothetical protein | 0.000000 | 0.07 |
| SAUSA300_1753 | splF | serine protease SplF | 0.000000 | 0.03 |
| SAUSA300_1754 | splE | serine protease SplE | 0.000000 | 0.03 |
| SAUSA300_1755 | splD | serine protease SplD | 0.000000 | 0.03 |
| SAUSA300_1756 | splC | serine protease SplC | 0.000000 | 0.03 |
| SAUSA300_1757 | splB | serine protease SplB | 0.000000 | 0.03 |
| SAUSA300_1758 | splA | serine protease SplA | 0.000000 | 0.03 |
| SAUSA300_1759 | . | conserved hypothetical protein | 0.022894 | 0.31 |
| SAUSA300_1760 | epiG | lantibiotic epidermin immunity protein F | 0.000000 | 0.03 |
| SAUSA300_1761 | epiE | lantibiotic epidermin immunity protein F | 0.000000 | 0.02 |
| SAUSA300_1762 | epiF | lantibiotic epidermin immunity protein F | 0.000000 | 0.01 |
| SAUSA300_1763 | epiP | lantibiotic epidermin leader peptide processing serine protease EpiP | 0.000000 | 0.04 |
| SAUSA300_1764 | epiD | lantibiotic epidermin biosynthesis protein EpiD | 0.030156 | 0.46 |
| SAUSA300_1765 | epiC | lantibiotic epidermin biosynthesis protein EpiC | 0.000000 | 0.38 |
| SAUSA300_1767 | epiA | lantibiotic epidermin biosynthesis protein EpiA | 0.000000 | 0.11 |
| SAUSA300_1770 | . | conserved hypothetical protein | 0.000001 | 0.48 |
| SAUSA300_1788 | . | conserved hypothetical protein | 0.000000 | 0.09 |
| SAUSA300_1790 | prsA | foldase protein PrsA precursor | 0.000000 | 0.50 |
| SAUSA300_1801 | fumC | fumarate hydratase, class II | 0.000000 | 0.21 |
| SAUSA300_1803 | . | conserved hypothetical protein | 0.000000 | 0.10 |
| SAUSA300_1804 | . | conserved hypothetical protein | 0.000000 | 0.09 |
| SAUSA300_1841 | rrsC | 16S ribosomal RNA | 0.001088 | 0.44 |
| SAUSA300_1843 | . | D-isomer specific 2-hydroxyacid dehydrogenase family protein | 0.000000 | 0.44 |
| SAUSA300_1844 | . | bacterioferritin comigratory protein | 0.000000 | 0.43 |
| SAUSA300_1854 | . | regulatory protein RecX | 0.000000 | 0.49 |
| SAUSA300_1856 | . | conserved hypothetical protein | 0.000000 | 0.08 |
| SAUSA300_1862 | . | conserved hypothetical protein | 0.000000 | 0.12 |
| SAUSA300_1863 | . | conserved hypothetical protein | 0.000000 | 0.16 |
| SAUSA300_1864 | . | putative membrane protein | 0.000000 | 0.09 |
| SAUSA300_1897 | . | sodium-dependent transporter | 0.000000 | 0.38 |
| SAUSA300_1898 | . | conserved hypothetical protein | 0.000000 | 0.17 |
| SAUSA300_1901 | aldA2 | aldehyde dehydrogenase | 0.000000 | 0.35 |
| SAUSA300_1904 | . | conserved hypothetical protein | 0.000000 | 0.08 |
| SAUSA300_1909 | . | conserved hypothetical protein | 0.000000 | 0.37 |
| SAUSA300_1925 | . | phiPVL ORF17-like protein | 0.000000 | 0.33 |
| SAUSA300_1926 | . | phi77 ORF044-like protein | 0.000000 | 0.24 |
| SAUSA300_1930 | . | phi77 ORF001-like protein, phage tail tape measure protein | 0.000000 | 0.43 |
| SAUSA300_1932 | . | conserved hypothetical phage protein | 0.000000 | 0.46 |
| SAUSA300_1934 | . | phi77 ORF020-like protein, phage major tail protein | 0.000002 | 0.47 |
| SAUSA300_1935 | . | phi77 ORF029-like protein | 0.000000 | 0.38 |
| SAUSA300_1936 | . | conserved hypothetical phage protein | 0.000003 | 0.36 |
| SAUSA300_1937 | . | phi77 ORF045-like protein | 0.000000 | 0.37 |
| SAUSA300_1965 | . | conserved hypothetical phage protein | 0.000000 | 0.12 |
| SAUSA300_1976 | . | probable succinyl-diaminopimelate desuccinylase | 0.000000 | 0.04 |
| SAUSA300_1980 | . | acetyltransferase, GNAT family | 0.000000 | 0.25 |
| SAUSA300_1987 | . | hydrolase, carbon-nitrogen family | 0.000000 | 0.47 |
| SAUSA300_1988 | . | delta-hemolysin precursor | 0.000000 | 0.25 |
| SAUSA300_1989 | agrB | accessory gene regulator protein B | 0.000000 | 0.07 |
| SAUSA300_1990 | agrD | accessory gene regulator protein D | 0.000000 | 0.09 |
| SAUSA300_1991 | agrC | accessory gene regulator protein C | 0.000000 | 0.08 |
| SAUSA300_1992 | agrA | accessory gene regulator protein A | 0.000000 | 0.09 |
| SAUSA300_2006 | ilvD | dihydroxy-acid dehydratase | 0.000000 | 0.27 |
| SAUSA300_2007 | ilvB | acetolactate synthase, large subunit | 0.000000 | 0.37 |
| SAUSA300_2009 | ilvC | ketol-acid reductoisomerase | 0.000000 | 0.25 |
| SAUSA300_2010 | leuA | 2-isopropylmalate synthase | 0.000000 | 0.25 |
| SAUSA300_2011 | leuB | 3-isopropylmalate dehydrogenase | 0.000000 | 0.22 |
| SAUSA300_2012 | leuC | 3-isopropylmalate dehydratase, large subunit | 0.000000 | 0.31 |
| SAUSA300_2013 | leuD | 3-isopropylmalate dehydratase, small subunit | 0.000000 | 0.31 |
| SAUSA300_2014 | ilvA | threonine dehydratase | 0.000000 | 0.29 |
| SAUSA300_2017 | rrsD | 16S ribosomal RNA | 0.001089 | 0.44 |
| SAUSA300_2022 | rpoF | RNA polymerase sigma-37 factor | 0.000000 | 0.40 |
| SAUSA300_2023 | rsbW | anti-sigma-B factor, serine-protein kinase | 0.000000 | 0.30 |
| SAUSA300_2024 | rsbV | anti-sigma-B factor, antagonist | 0.000000 | 0.23 |
| SAUSA300_2041 | . | conserved hypothetical protein | 0.000000 | 0.08 |
| SAUSA300_2052 | . | single-stranded DNA- binding protein family | 0.000000 | 0.27 |
| SAUSA300_2053 | . | conserved hypothetical protein | 0.000000 | 0.46 |
| SAUSA300_2056 | . | conserved hypothetical protein | 0.000000 | 0.17 |
| SAUSA300_2065 | . | UDP-N-acetylglucosamine 2-epimerase | 0.000000 | 0.38 |
| SAUSA300_2066 | upp | uracil phosphoribosyltransferase | 0.000000 | 0.35 |
| SAUSA300_2067 | glyA | serine hydroxymethyltransferase | 0.000000 | 0.36 |
| SAUSA300_2068 | . | conserved hypothetical protein | 0.000000 | 0.42 |
| SAUSA300_2076 | . | aldehyde dehydrogenase family protein | 0.000000 | 0.24 |
| SAUSA300_2089 | pdp | pyrimidine nucleoside phosphorylase | 0.000000 | 0.31 |
| SAUSA300_2090 | deoC | deoxyribose-phosphate aldolase | 0.000000 | 0.42 |
| SAUSA300_2091 | deoD | purine nucleoside phosphorylase | 0.000000 | 0.48 |
| SAUSA300_2097 | . | conserved hypothetical protein | 0.000000 | 0.05 |
| SAUSA300_2104 | glmS | glucosamine--fructose-6-phosphate aminotransferase (isomerizing) | 0.000000 | 0.10 |
| SAUSA300_2105 | mtlF | PTS system, mannitol specific IIBC component | 0.000000 | 0.33 |
| SAUSA300_2106 | . | putative transcriptional regulator | 0.000000 | 0.09 |
| SAUSA300_2107 | mtlA | PTS system, mannitol specific IIA component | 0.000000 | 0.05 |
| SAUSA300_2108 | mtlD | Mannitol-1-phosphate 5-dehydrogenase | 0.000000 | 0.06 |
| SAUSA300_2114 | rocF | arginase | 0.000000 | 0.21 |
| SAUSA300_2124 | rrsE | 16S ribosomal RNA | 0.001110 | 0.44 |
| SAUSA300_2132 | . | conserved hypothetical protein | 0.000000 | 0.11 |
| SAUSA300_2137 | . | conserved hypothetical protein | 0.000000 | 0.31 |
| SAUSA300_2138 | . | conserved hypothetical protein | 0.000000 | 0.25 |
| SAUSA300_2142 | asp23 | alkaline shock protein 23 | 0.000000 | 0.04 |
| SAUSA300_2143 | . | conserved hypothetical protein | 0.000000 | 0.03 |
| SAUSA300_2144 | . | conserved hypothetical protein | 0.000000 | 0.03 |
| SAUSA300_2145 | . | glycine betaine transporter | 0.000000 | 0.02 |
| SAUSA300_2147 | . | alcohol dehydrogenase, zinc-containing | 0.000000 | 0.49 |
| SAUSA300_2150 | lacE | PTS system, lactose-specific IIBC component | 0.000059 | 0.45 |
| SAUSA300_2151 | lacF | PTS system, lactose-specific IIA component | 0.017151 | 0.34 |
| SAUSA300_2152 | lacD | tagatose 1,6-diphosphate aldolase | 0.000000 | 0.10 |
| SAUSA300_2153 | lacC | tagatose-6-phosphate kinase | 0.000000 | 0.15 |
| SAUSA300_2154 | lacB | galactose-6-phosphate isomerase | 0.000000 | 0.21 |
| SAUSA300_2155 | lacA | galactose-6-phosphate isomerase | 0.000000 | 0.24 |
| SAUSA300_2159 | . | aldo/keto reductase family protein | 0.000000 | 0.35 |
| SAUSA300_2160 | . | transcriptional regulator, MerR family | 0.000000 | 0.40 |
| SAUSA300_2164 | . | conserved hypothetical protein | 0.000000 | 0.00 |
| SAUSA300_2169 | . | conserved hypothetical protein | 0.000000 | 0.47 |
| SAUSA300_2236 | . | conserved hypothetical protein | 0.000000 | 0.36 |
| SAUSA300_2252 | . | conserved hypothetical protein | 0.000000 | 0.48 |
| SAUSA300_2254 | . | glycerate dehydrogenase-like protein | 0.000000 | 0.31 |
| SAUSA300_2257 | . | conserved hypothetical protein | 0.000000 | 0.18 |
| SAUSA300_2258 | . | formate dehydrogenase, alpha subunit | 0.000000 | 0.18 |
| SAUSA300_2259 | . | putative transcriptional regulator | 0.000000 | 0.45 |
| SAUSA300_2260 | . | inositol monophosphatase family protein | 0.000000 | 0.19 |
| SAUSA300_2270 | glvC | PTS system, arbutin-like IIBC component | 0.000000 | 0.06 |
| SAUSA300_2274 | . | putative membrane protein | 0.000000 | 0.39 |
| SAUSA300_2275 | . | oxidoreductase, short chain dehydrogenase/reductase family | 0.000000 | 0.02 |
| SAUSA300_2277 | hutI | imidazolonepropionase | 0.000000 | 0.29 |
| SAUSA300_2278 | hutU | urocanate hydratase | 0.000000 | 0.28 |
| SAUSA300_2279 | . | LysR family regulatory protein | 0.000000 | 0.39 |
| SAUSA300_2281 | hutG | formimidoylglutamase | 0.000000 | 0.06 |
| SAUSA300_2310 | . | conserved hypothetical protein | 0.001961 | 0.49 |
| SAUSA300_2312 | mqo | malate:quinone-oxidoreductase | 0.000000 | 0.42 |
| SAUSA300_2315 | . | putative lipoprotein | 0.000000 | 0.07 |
| SAUSA300_2325 | . | conserved hypothetical protein | 0.000000 | 0.31 |
| SAUSA300_2327 | . | conserved hypothetical protein | 0.000000 | 0.08 |
| SAUSA300_2365 | hlgA | gamma-hemolysin component A | 0.000000 | 0.15 |
| SAUSA300_2376 | . | conserved hypothetical protein | 0.000000 | 0.33 |
| SAUSA300_2383 | . | amino acid permease | 0.000000 | 0.22 |
| SAUSA300_2390 | opuCd | glycine betaine/carnitine/choline transport system permease | 0.000000 | 0.28 |
| SAUSA300_2391 | opuCc | glycine betaine/carnitine/choline ABC transporter | 0.000000 | 0.31 |
| SAUSA300_2392 | opuCb | glycine betaine/carnitine/choline ABC transporter | 0.000000 | 0.45 |
| SAUSA300_2393 | opuCa | glycine betaine/carnitine/choline ABC transporter ATP-binding protein | 0.000000 | 0.47 |
| SAUSA300_2396 | pnbA | para-nitrobenzyl esterase | 0.000000 | 0.14 |
| SAUSA300_2398 | . | putative membrane protein | 0.000000 | 0.07 |
| SAUSA300_2399 | . | ABC transporter, ATP-binding protein | 0.000000 | 0.21 |
| SAUSA300_2415 | . | conserved hypothetical protein | 0.000000 | 0.05 |
| SAUSA300_2416 | . | glucose 1-dehydrogenase-like protein | 0.000000 | 0.04 |
| SAUSA300_2417 | . | putative transporter | 0.000000 | 0.37 |
| SAUSA300_2418 | . | conserved hypothetical protein | 0.000000 | 0.02 |
| SAUSA300_2423 | . | conserved hypothetical protein | 0.000000 | 0.26 |
| SAUSA300_2447 | . | conserved hypothetical protein | 0.000000 | 0.13 |
| SAUSA300_2449 | . | putative transporter | 0.000000 | 0.31 |
| SAUSA300_2455 | . | putative fructose-1,6-bisphosphatase | 0.000000 | 0.28 |
| SAUSA300_2457 | . | phospholipase/carboxylesterase family protein | 0.000000 | 0.22 |
| SAUSA300_2458 | . | glyoxylase family protein | 0.000000 | 0.25 |
| SAUSA300_2460 | . | acetyltransferase family protein | 0.000000 | 0.37 |
| SAUSA300_2463 | ddh | D-lactate dehydrogenase | 0.000000 | 0.31 |
| SAUSA300_2464 | . | hydrolase, haloacid dehalogenase-like family | 0.000000 | 0.49 |
| SAUSA300_2477 | cidC | pyruvate oxidase | 0.000000 | 0.13 |
| SAUSA300_2478 | cidB | Holin-like protein cidB | 0.000000 | 0.10 |
| SAUSA300_2486 | . | putative ATP-dependent Clp proteinase | 0.000000 | 0.02 |
| SAUSA300_2491 | . | 1-pyrroline-5-carboxylate dehydrogenase | 0.000000 | 0.22 |
| SAUSA300_2498 | crtN | squalene synthase | 0.000000 | 0.04 |
| SAUSA300_2499 | crtM | squalene desaturase | 0.000000 | 0.03 |
| SAUSA300_2500 | . | glycosyl transferase | 0.000000 | 0.02 |
| SAUSA300_2501 | . | phytoene dehydrogenase | 0.000000 | 0.03 |
| SAUSA300_2502 | . | conserved hypothetical protein | 0.000000 | 0.03 |
| SAUSA300_2517 | . | amidohydrolase family protein | 0.000000 | 0.10 |
| SAUSA300_2518 | . | hydrolase family protein | 0.000000 | 0.05 |
| SAUSA300_2523 | . | conserved hypothetical protein | 0.000075 | 0.44 |
| SAUSA300_2524 | . | conserved hypothetical protein | 0.000000 | 0.33 |
| SAUSA300_2525 | . | conserved hypothetical protein | 0.000000 | 0.04 |
| SAUSA300_2529 | . | conserved hypothetical protein | 0.000000 | 0.44 |
| SAUSA300_2540 | . | fructose-bisphosphate aldolase class-I | 0.000000 | 0.39 |
| SAUSA300_2542 | . | putative AMP-binding enzyme | 0.000000 | 0.25 |
| SAUSA300_2543 | . | conserved hypothetical protein | 0.000000 | 0.06 |
| SAUSA300_2544 | . | conserved hypothetical protein | 0.000000 | 0.44 |
| SAUSA300_2546 | betB | glycine betaine aldehyde dehydrogenase | 0.000002 | 0.39 |
| SAUSA300_2548 | . | conserved hypothetical protein | 0.000000 | 0.20 |
| SAUSA300_2550 | nrdG | anaerobic ribonucleotide reductase, small subunit | 0.000000 | 0.13 |
| SAUSA300_2551 | nrdD | anaerobic ribonucleotide reductase, large subunit | 0.000000 | 0.16 |
| SAUSA300_2568 | arcD | arginine/ornithine antiporter | 0.000000 | 0.20 |
| SAUSA300_2569 | arcB | ornithine carbamoyltransferase | 0.000000 | 0.20 |
| SAUSA300_2570 | arcA | arginine deiminase | 0.000000 | 0.13 |
| SAUSA300_2573 | isaB | immunodominant antigen B | 0.000000 | 0.11 |
| SAUSA300_2576 | . | phosphotransferase system, fructose-specific IIABC component | 0.000000 | 0.47 |
| SAUSA300_2577 | manA | mannose-6-phosphate isomerase, class I | 0.000002 | 0.46 |
| SAUSA300_2580 | . | isochorismatase family protein | 0.000000 | 0.36 |
| SAUSA300_2581 | . | putative surface anchored protein | 0.000000 | 0.19 |
| SAUSA300_2582 | . | conserved hypothetical protein | 0.000000 | 0.24 |
| SAUSA300_2583 | . | putative glycosyl transferase | 0.000000 | 0.19 |
| SAUSA300_2584 | . | preprotein translocase, secA protein | 0.000000 | 0.14 |
| SAUSA300_2590 | . | conserved hypothetical protein | 0.000000 | 0.50 |
| SAUSA300_2592 | . | conserved hypothetical protein | 0.000000 | 0.09 |
| SAUSA300_2593 | . | conserved hypothetical protein | 0.000000 | 0.22 |
| SAUSA300_2603 | lip | triacylglycerol lipase precursor | 0.000000 | 0.03 |
| SAUSA300_2620 | . | conserved hypothetical protein | 0.000000 | 0.11 |
| SAUSA300_2621 | . | conserved hypothetical protein | 0.000001 | 0.39 |
| SAUSA300_2626 | . | conserved hypothetical protein | 0.000000 | 0.10 |
| SAUSA300_2629 | . | conserved hypothetical protein | 0.000000 | 0.11 |
| SAUSA300_2630 | nixA | high-affinity nickel-transporter | 0.000000 | 0.38 |
| SAUSA300_2632 | . | putative membrane protein | 0.000000 | 0.04 |

**Supplementary Table 5. Genes regulated by all three compounds (STZ, FU and CP).**

**Up-regulated (11)**

| **Gene ID** | **Name** | **Product** |
| --- | --- | --- |
| **SAUSA300_0114** | sarS | Staphylococcal accessory regulator A homolog |
| **SAUSA300_0715** | nrdI | Protein involved in ribonucleotide reduction |
| **SAUSA300_0716** |  | Ribonucleotide reductase alpha subunit |
| **SAUSA300_0717** | nrdF | Ribonucleotide reductase beta subunit |
| **SAUSA300_0741** | uvrB | Exinuclease ABC subunit B |
| **SAUSA300_0742** | uvrA | Exinuclease ABC subunit A |
| **SAUSA300_1178** | recA | Recombinase A |
| **SAUSA300_1242** | sbcD | SbcD nuclease |
| **SAUSA300_1259** |  | ImpB/MucB/SamB family protein |
| **SAUSA300_2372** | bioA | Adenosylmethionine-8-amino-7-oxononanoate aminotransferase |
| **SAUSA300_2373** | bioD | Dethiobiotin synthase |

**Down-regulated (11)**

| **Gene ID** | **Name** | **Product** |
| --- | --- | --- |
| **SAUSA300_0605** | sarA | Staphylococcal accessory regulator A |
| **SAUSA300_1058** | hla | Alpha-hemolysin precursor |
| **SAUSA300_1122** | plsX | Fatty acid/phospholipid synthesis protein PlsX |
| **SAUSA300_1123** | fabD | Malonyl CoA-acyl carrier protein transacylase |
| **SAUSA300_1145** | xerC | XerC site-specifc recombinase |
| **SAUSA300_1235** | guaC | GMP oxidoreductase |
| **SAUSA300_1371** | recQ2 | ATP-dependent DNA helicase, RecQ family |
| **SAUSA300_1708** | rot | Repressor of toxins Rot |
| **SAUSA300_1756** | splC | Serine protease SplC |
| **SAUSA300_1854** | recX | RecX regulatory protein |
| **SAUSA300_1989** | agrB | Accessory gene regulator protein B |

**Supplementary Table 6**. Bacterial strains and plasmids used in this study

| **Strain or Plasmid** | **Relevant characteristic** | **Origin** |
| --- | --- | --- |
| *E. coli* |  |  |
| **DH5α** | Plasmid-free, restriction deficient | New England Biolabs |
| *S. aureus* |  |  |
| **RN4220** | Restriction deficient, prophage cured | 1 |
| **USA300-P23** | USA300-0114 without plasmid 2 and 3 | 2 |
|  |  |  |
| **Plasmid** |  |  |
| **pYJ335** | An *E. coli-S. aureus* shuttle vector, Erm^r^ | 3 |
| **pYJ335-*gfp*** | pYJ335 carrying a promoterless *gfp* | 4 |
| **pYJ335-P1-*gfp*** | pYJ335 carrying the P1promoter-*gfp* fusion | This study |
| **pCL-P_hlamin_-*gfp*** | pCL55 carrying P_hlamin-_*gfp* fusion | This study |
| **pCL-PmecA-gfp** | pCL55 carrying PmecA-*gfp* fusion | This study |
| **pCL-PhrtB*gfp*** | pCL55 carrying PhrtB-*gfp* fusion | This study |

**Supplementary Table 7. Primers used in this study**

| **Name** | **Sequence (5′→ 3′)** | **Target** |
| --- | --- | --- |
| P1969 | GGGGTACCATTGGAAGTGGATAACATGTCAAAAGGAGAA  GAATTATTTAC | pYJ-*gfp* |
| P1747 | ATTGGATTGGAAGTACGGTACCGAGCTCGAATTCACTG | pYJ-*gfp* |
| P1971 | TACTTCCAATCCAATGTCATCATTGGTGGTATTATGTTG | P1 |
| P1972 | TTATCCACTTCCAATGGCTAACTCCTCATTTCTTCAATT | P1 |
| P1990 | ATTGGATTGGAAGTAC CGGAGGAGGGATGTAAAATGTGG | pCL55-*gfp* |
| P1991 | ATTGGAAGTGGATAAC CAAAAGGAGAACGCATAATGTC | pCL55-*gfp* |
| P1992 | TTATCCACTTCCAATG attacaatataaaaataca aatatcttag | Phla |
| P1993 | TACTTCCAATCCAATG TTAATATATAGTTAATTTTTATTTAATAG | Phla |
| P3014 | ACATTATGCGTTCTCCTTTTccaaacccgacaactacaactattaaaataagt | PmecA |
| P3015 | TCCCTCCTCCGGGATCCCCGgcattctcactaaaaaaattacacatatcgtgagc | PmecA |
| P3016 | aatttttttagtgagaatgcCGGGGATCCCGGAGG | pCL55-*gfp* |
| P3017 | tgtagttgtcgggtttggAAAAGGAGAACGCATAATGTCAAAAGGAGAAGAATTATTTAC | pCL55-*gfp* |
| P3284 | ATTATGCGTTCTCCTTTTGgtaaaacataatctcttttatcgctaatttcatatcgattc | PhrtB |
| P3285 | CATTTTACATCCCTCCTCCGcgaggatcgtcatcgacaaca | PhrtB |
| P3286 | gttgtcgatgacgatcctcgCGGAGGAGGGATGTAAAATGTGGTTTG | pCL55-*gfp* |
| P3287 | taaaagagattatgttttacCAAAAGGAGAACGCATAATGTCAAAAG | pCL55-*gfp* |
| P3137 | AGATGACATGCCTGGCCTAC | *agrC* |
| P3138 | CGGAAAATTCATTTCTTGTGC | *agrC* |
| P3125 | ACCGTTATTTTGACCGCGTA | *arlS* |
| P3126 | GCCAGCGCAATGATATACAA | *arlS* |
| P3127 | GAGCCGGCTAAATAGTGTCG | *srrB* |
| P3128 | TCATCAACCCACCAGGATTT | *srrB* |
| P1419 | TGTTTGCTTCAGTGATTCGT | *sarA* |
| P1420 | CAACCACAAGTTGTTAAAGCAG | *sarA* |
| P3099 | CAAATGATCACAGCATTTGGTACAG | *gyrB* |
| P3100 | CGGCATCAGTCATAATGACGAT | *gyrB* |
| P2898 | AAACGGTGAAACTGTTGAAGG | *saeP* |
| P2899 | CGTAGTCAACCATTGCGATTT | *saeP* |
| P2900 | GAAAAATTAACGGGCGGATT | *saeQ* |
| P2901 | ATTGCAATCTCTCCGAGTGG | *saeQ* |
| P2904 | ACGCCACTTGAGCGTATTTT | *saeS* |
| P2905 | AATCCAGAACCACCCGTTTT | *saeS* |

**References**

1 Kreiswirth, B. N. *et al.* The toxic shock syndrome exotoxin structural gene is not detectably transmitted by a prophage. *Nature* **305**, 709-712. (1983).

2 Jeong, D. W. *et al.* Identification of P3 promoter and distinct roles of the two promoters of the SaeRS two-component system in *Staphylococcus aureus.* *J Bacteriol* **193**, 4672-4684 (2011).

3 Ji, Y., Marra, A., Rosenberg, M. & Woodnutt, G. Regulated antisense RNA eliminates alpha-toxin virulence in *Staphylococcus aureus* infection. *J Bacteriol* **181**, 6585-6590. (1999).

4 Liu, Q., Cho, H., Yeo, W. S. & Bae, T. The Extracytoplasmic Linker Peptide of the Sensor Protein SaeS Tunes the Kinase Activity Required for Staphylococcal Virulence in Response to Host Signals. *PLoS Pathog* **11**, e1004799 (2015).
